# Image-based consensus molecular subtype (imCMS) classification of colorectal cancer using deep learning

**DOI:** 10.1136/gutjnl-2019-319866

**Published:** 2020-07-20

**Authors:** Korsuk Sirinukunwattana, Enric Domingo, Susan D Richman, Keara L Redmond, Andrew Blake, Clare Verrill, Simon J Leedham, Aikaterini Chatzipli, Claire Hardy, Celina M Whalley, Chieh-hsi Wu, Andrew D Beggs, Ultan McDermott, Philip D Dunne, Angela Meade, Steven M Walker, Graeme I Murray, Leslie Samuel, Matthew Seymour, Ian Tomlinson, Phil Quirke, Timothy Maughan, Jens Rittscher, Viktor H Koelzer, S:CORT consortium

**Affiliations:** 1 Institute of Biomedical Engineering (IBME), Department of Engineering Science, University of Oxford, Oxford, UK; 2 Big Data Institute, University of Oxford, Li Ka Shing Centre for Health Information and Discovery, Oxford, UK; 3 Oxford NIHR Biomedical Research Centre, Oxford University Hospitals Trust, Oxford, UK; 4 Department of Oncology, University of Oxford, Oxford, UK; 5 Department of Pathology and Tumour Biology, Leeds Institute of Cancer and Pathology, Leeds, UK; 6 Centre for Cancer Research and Cell Biology, Faculty of Medicine, Health and Life Sciences, Queen's University Belfast, Belfast, UK; 7 Department of Cellular Pathology, Oxford University Hospitals NHS Foundation Trust, Oxford, UK; 8 Nuffield Department of Surgical Sciences and NIHR Oxford Biomedical Research Centre, University of Oxford, Oxford, UK; 9 Gastrointestinal Stem-cell Biology Laboratory, Oxford Centre for Cancer Gene Research, Wellcome Trust Centre for Human Genetics, Oxford, UK; 10 Translational Gastroenterology Unit, Experimental Medicine Division, Nuffield Department of Clinical Medicine, John Radcliffe Hospital, University of Oxford, Oxford, UK; 11 Wellcome Trust Sanger Institute, Hinxton, UK; 12 Institute of Cancer and Genomic Sciences, University of Birmingham, Birmingham, UK; 13 Department of Statistics, University of Oxford, Oxford, UK; 14 School of Cancer Sciences, University of Birmingham, Birmingham, UK; 15 Centre for Cancer Research and Cell Biology, Queen's University Belfast, Belfast, UK; 16 MRC Clinical Trials Unit at University College London, London, UK; 17 Almac Diagnostics Ltd, Craigavon, UK; 18 Department of Pathology, School of Medicine, Medical Sciences and Nutrition, University of Aberdeen, Aberdeen, UK; 19 Department of Clinical Oncology, Aberdeen Royal Infirmary, Aberdeen, UK; 20 Department of Oncology, Leeds Institute of Cancer and Pathology, Leeds, UK; 21 Edinburgh Cancer Centre, MRC Institute of Genetics and Molecular Medicine, University of Edinburgh, Edinburgh, UK; 22 CRUK/MRC Oxford Institute for Radiation Oncology, University of Oxford, Oxford, UK; 23 Ludwig Institute for Cancer Research, Nuffield Department of Clinical Medicine, University of Oxford, Oxford, UK; 24 Nuffield Department of Medicine, University of Oxford, Oxford, UK; 25 Department of Pathology and Molecular Pathology, University of Zurich, Zurich, Switzerland

**Keywords:** colorectal pathology, computerised image analysis, molecular pathology

## Abstract

**Objective:**

Complex phenotypes captured on histological slides represent the biological processes at play in individual cancers, but the link to underlying molecular classification has not been clarified or systematised. In colorectal cancer (CRC), histological grading is a poor predictor of disease progression, and consensus molecular subtypes (CMSs) cannot be distinguished without gene expression profiling. We hypothesise that image analysis is a cost-effective tool to associate complex features of tissue organisation with molecular and outcome data and to resolve unclassifiable or heterogeneous cases. In this study, we present an image-based approach to predict CRC CMS from standard H&E sections using deep learning.

**Design:**

Training and evaluation of a neural network were performed using a total of n=1206 tissue sections with comprehensive multi-omic data from three independent datasets (training on FOCUS trial, n=278 patients; test on rectal cancer biopsies, GRAMPIAN cohort, n=144 patients; and The Cancer Genome Atlas (TCGA), n=430 patients). Ground truth CMS calls were ascertained by matching random forest and single sample predictions from CMS classifier.

**Results:**

Image-based CMS (imCMS) accurately classified slides in unseen datasets from TCGA (n=431 slides, AUC)=0.84) and rectal cancer biopsies (n=265 slides, AUC=0.85). imCMS spatially resolved intratumoural heterogeneity and provided secondary calls correlating with bioinformatic prediction from molecular data. imCMS classified samples previously unclassifiable by RNA expression profiling, reproduced the expected correlations with genomic and epigenetic alterations and showed similar prognostic associations as transcriptomic CMS.

**Conclusion:**

This study shows that a prediction of RNA expression classifiers can be made from H&E images, opening the door to simple, cheap and reliable biological stratification within routine workflows.

**Significance of this study**

**What is already known on this subject?**

- Previous research has shown that there are four distinct colorectal cancer subtypes defined by common patterns of gene expression.
- Clinicians hope that these consensus molecular subtypes (CMSs) of colorectal cancer could support patient stratification for precision therapy.
- Currently, colorectal cancer subtype is established through RNA analysis, which is not widely used due to high costs and the need for specialist knowledge to interpret the data.

**What are the new findings?**

- Computational models can predict transcriptional subtypes of colorectal cancer from standard histology sections.
- Image-based CMS (imCMS) makes sequencing information interpretable through association of tile level predictions with morphology, molecular features and outcome data.
- imCMS classifies samples previously unclassifiable by RNA expression profiling, gives a novel insight into tumour heterogeneity and shows similar prognostic associations as transcriptomic CMS.
- imCMS classifies endoscopic biopsies and resection specimens of colorectal cancer laying the methodological basis for patient stratification in diverse clinical settings.

**How might it impact on clinical practice in the foreseeable future?**

- A prediction of RNA expression classifiers can be made from H&E images, opening the door to simple, cheap and reliable biological stratification within routine workflows and existing retrospective cohorts.

## Introduction

Colorectal cancer (CRC) is a disease with heterogeneous molecular subtypes, variable clinical course and prognosis.^1^ An increasing understanding of CRC biology has led to the development of targeted treatments directed against key pro-oncogenic signalling pathways, but these treatments are only effective in a small proportion of patients.^2^ Molecular stratification of patients with CRC is essential to form homogeneous subgroups for targeted treatment and prognosis.^3^ Next generation sequencing technologies enable the multi-omic profiling of malignant tumours but mutation and copy number data have been of limited impact in CRC, while more informative RNA analyses are more costly, difficult to standardise and require data storage and bioinformatics expertise.^4 5^ In contrast, histopathology slides are inexpensive to produce and principal stains such as H&E are firmly established in the pathology laboratory.

The application of traditional image analysis to histopathology facilitates the quantitative assessment of tissue architecture, cell distribution and cellular morphology by light microscopy to generate feature libraries of unprecedented resolution and detail.^6^ More recently, deep learning is being used to capture morphological differences with a precision that exceeds human performance. Coudray *et al* 7 use this approach to detect targetable oncogenic driver mutations in lung cancer using deep neural classification networks. By combining an image-based analysis with molecular characterisation, it is now feasible to identify novel genotype–phenotype correlations. Complex multi-scale morphological traits as well as genomic alterations can now be characterised at scale and with short turnaround times. Given that H&E processing allows analysis of large tissue sections within existing clinical workflows, the discovery of morpho-molecular correlations holds the promise of improving patient stratification in clinical practice through the development of new image-based biomarkers.^8^

In CRC, it is well known that tumour morphology, growth pattern and architecture hold important clues to differentiating biological subtypes with clinical impact.^9^ The composition of the tumour microenvironment is a key component determining the tumour progression and therapy response.^10^ Tumour and non-tumour tissue contributes to image information on the histological slide and to the consensus molecular subtype (CMS) classification of CRC at the transcriptional level.^11^ The CMS classification distinguishes four groups of CRC with distinct clinical behaviour and underpinning biology. These include CMS1 (14%; microsatellite instability immune, favourable prognosis in early-stage disease, adverse prognosis in the metastatic setting), CMS2 (37%; canonical, epithelial gene expression profile, WNT and MYC signalling activation, intermediate prognosis), CMS3 (13%; epithelial profile with evident metabolic dysregulation, intermediate prognosis) and CMS4 (23%; mesenchymal, prominent transforming growth factor-β activation, poor prognosis).^1 11^ Samples with transitioning phenotypes or intratumoural heterogeneity are presently considered to be unclassifiable (13%). Prior studies have shown the feasibility of developing image-based biomarkers for molecular subtypes of CRC by deep learning.^12^ An association of specific imaging features with meta-gene expression profiles was described and preliminary data were subsequently reported indicating that histology slides may contain sufficient information to predict the CMS molecular subtypes of CRC.^13^

Ongoing research is investigating associations between clinical interventions and CMS subgrouping, and so this classification has the potential to guide treatment allocation in future clinical practice.^1 11^ However, clinical implementation of the CMS classification has been held back by the considerable costs of RNA sequencing, the inability to obtain confident CMS calls from single samples bioinformatically, intratumoural heterogeneity and high levels of unclassified calls when limited material is available.^11 14 15^ Here, we derive a novel image-based CMS (imCMS) classification from H&E-stained tissue sections sourced from the Medical Research Council (MRC) and Cancer Research UK (CRUK) Stratification in COloRecTal cancer (S:CORT) programme and The Cancer Genome Atlas (TCGA). We demonstrate the existence of distinct image phenotypes of CRC that reproducibly associate with CMS transcriptional classification, key oncogenic driver mutations and prognosis. Automatic, high-fidelity classification of three independent clinical cohorts including preoperative biopsies underlines the applicability of this approach to heterogeneous sample sets and relevant clinical settings. Our analysis provides a more localised analysis than is currently possible using routine RNA sequencing. Hence, imCMS allows us to analyse the spatial variation in the tissue, providing new ways of assessing tumour heterogeneity. In three cohorts including tissue samples from highly diverse clinical settings, imCMS successfully classified CRC samples that were previously considered to have unknown biological and clinical behaviour and failed transcriptional classification. imCMS classification is standardised, inexpensive and could be carried out in a telepathology setting on routinely available H&E sections. This opens up new avenues for the translation of the transcriptional classification of CRC into clinical practice and has the potential to increase availability of molecular stratification in low resource settings.

## Materials and methods

### Study design

**Figure 1:**
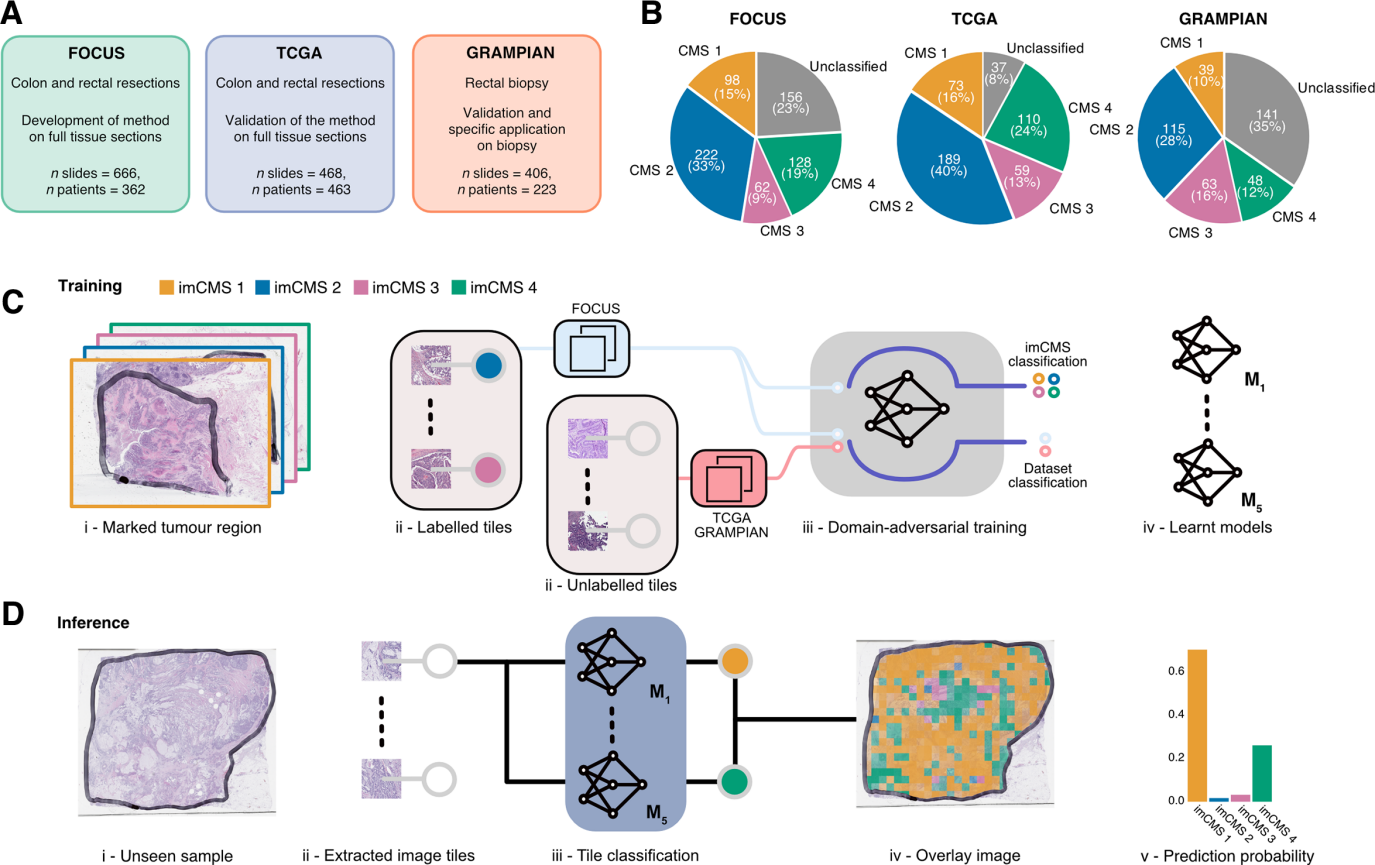
Data, study design and imCMS classification framework. Three independent datasets (FOCUS, TCGA and GRAMPIAN) were used in this study. (A) The distribution of the samples stratified by the CMS calls in each dataset. (B) The FOCUS dataset was primarily used for learning the imCMS discriminative model, while the TCGA and GRAMPIAN datasets were used for testing. (C) Training of the imCMS discriminative model based on the domain adversarial approach. Image tiles were extracted from annotated tumour regions. Tiles from the FOCUS cohort were categorised by CMS class of the original slide and were used to train the model to predict the imCMS classes on unseen datasets. Tiles from the TCGA and GRAMPIAN cohorts were unlabelled and were used together with those from the FOCUS cohort in the cohort (domain) prediction. Domain adversarial training forced the cohort classifier to perform poorly, which in turn encouraged the model to learn indiscriminative features across datasets. Five distinct models were produced. (D) At the inference time, the ensemble of the learnt models predicts the imCMS class for each of the image tiles extracted from annotated tumour regions of a slide. A slide is assigned to the imCMS class with the maximum prediction score (ie, highest number of tiles in the slide). imCMS, image-based consensus molecular subtype; TCGA, The Cancer Genome Atlas.

The study design, cohorts and aims are outlined in figure 1. Detailed methods for all studies are provided in the online supplementary materials and methods.

10.1136/gutjnl-2019-319866.supp1 Supplementary data

### Patient and public involvement

This project is tackling research gaps (RGs) identified by authors of this study (IT, VHK) in the Bowel Cancer UK ‘Critical research gaps and recommendations to inform research prioritisation for more effective prevention and improved outcomes in colorectal cancer’ research project, published in *Gut* in 2018.^16^ Briefly, RGs in CRC were identified by a multidisciplinary panel of patients, clinicians and researchers (n=71) organised in eight working groups as described in https://gut.bmj.com/content/67/1/179%23DC1. Draft papers developed by each working group were evaluated by a 20-strong patient panel. A final list of RGs and research recommendations (RR) was endorsed by all participants. Key RGs were identified in the pathological assessment of CRC (RG7). The working group highlighted the importance of integrating genomics, big data science and digital pathology methods to improve the morpho-molecular taxonomy and biological stratification of CRC (RR7.1, R.2, RG7.3, RG8). The present study aims to address these recommendations through the development of multiparameter algorithms for analysis of routine H&E slides in the pathology laboratory. Results from this study were communicated to the patient community through Bowel Cancer UK.^17 18^

## Results

### A deep learning framework for imCMS classification of CRC histology slides

The aim of this study was to develop an image analysis framework to associate features of tissue organisation on standard histology slides with molecular classification and outcome data in patients with CRC. Training and test cohorts were selected to represent relevant clinical scenarios in the management of patients with CRC including postoperative resection specimens (FOCUS and TCGA) and endoscopic biopsy material (GRAMPIAN). A total of 1540 slides from three independent datasets were utilised in this study including 666 slides of resection specimens from 362 patients in the FOCUS cohort, 468 slides of resection specimens from 463 patients in the TCGA cohort and 406 slides from preoperative biopsies of 223 patients in the GRAMPIAN cohort (figure 1A). Tumour areas on each slide were annotated by a pathologist, and the molecular analysis was performed on material obtained from strict serial sections to derive the CMS calls (figure 1B). Clinical and molecular data are summarised in online supplementary table S1.

10.1136/gutjnl-2019-319866.supp12 Supplementary data

The imCMS classifier was trained against CMS calls on the transcriptionally classified samples of the FOCUS cohort (510 slides, 278 patients) and tested on the TCGA (431 slides, 430 patients) and GRAMPIAN (265 slides, 144 patients) cohorts (online supplementary materials and methods). With the assumption that each CMS class is associated with unique histological patterns localised in different regions of the tumours,^14^ inception V3^19^ deep neural networks were trained for prediction of CMS calls for small overlapped image regions (tiles) of 512×512 pixels within the annotated regions (figure 1C). The size distribution of annotated areas per slide and the number of tiles per slide is shown in online supplementary figure S1. The imCMS class, prediction score and spatial location for each tile were recorded. An overall imCMS call for each slide was assigned based on the majority classification of tiles (figure 1D).

10.1136/gutjnl-2019-319866.supp2 Supplementary data

### Evaluation of imCMS classification

We systematically compared the performance of the imCMS classifier across all three cohorts. For benchmarking against molecular data, all unclassified samples were excluded from the test set. Classification performance was compared using image tiles derived at (a) 3× and (b) 12× magnification to determine the effect of detail levels. In the FOCUS training cohort, a robust imCMS classification performance of 0.88 AUC was reached (table 1, online supplementary table S2, S3). imCMS classification was then tested on the unseen TCGA and GRAMPIAN cohorts (table 1, online supplementary tables S2, S3). In general, imCMS trained at 3× provides comparable classification with that trained at 12× on whole tissue sections (AUC FOCUS: 0.88 at 3× vs 0.87 at 12×, p value=0.084; TCGA: 0.81 at 3× vs 0.8 at 12×, p value=0.058; GRAMPIAN: 0.82 at 3× vs 0.83 at 12×, p value=0.427, DeLong’s test^20^).

10.1136/gutjnl-2019-319866.supp13 Supplementary data

10.1136/gutjnl-2019-319866.supp14 Supplementary data

**Table 1:** Area under the curve with 95% CIs achieved by the imCMS classifier

CMS class	FOCUS n slides=510 n patients=278		TCGA n slides=431 n patients=430		GRAMPIAN n slides=265 n patients=144	
							3×	12×	3×	12×	3×	12×
CMS1	0.83 (0.78 to 0.87)	0.85 (0.81 to 0.89)	0.82 (0.77 to 0.89)	0.82 (0.76 to 0.88)	0.76 (0.65 to 0.85)	0.82 (0.75 to 0.92)
CMS2	0.88 (0.85 to 0.92)	0.86 (0.83 to 0.91)	0.84 (0.81 to 0.88)	0.82 (0.78 to 0.86)	0.78 (0.72 to 0.83)	0.75 (0.71 to 0.82)
CMS3	0.93 (0.91 to 0.97)	0.9 (0.85 to 0.94)	0.75 (0.67 to 0.83)	0.74 (0.68 to 0.81)	0.81 (0.75 to 0.9)	0.79 (0.73 to 0.88)
CMS4	0.87 (0.83 to 0.9)	0.85 (0.82 to 0.89)	0.84 (0.79 to 0.88)	0.82 (0.77 to 0.87)	0.94 (0.9 to 0.98)	0.94 (0.91 to 0.99)
Macro-average	0.88 (0.86 to 0.9)	0.87 (0.84 to 0.89)	0.81 (0.78 to 0.84)	0.8 (0.77 to 0.83)	0.82 (0.78 to 0.87)	0.83 (0.79 to 0.86)

CMS, consensus molecular subtype; imCMS, image-based consensus molecular subtype; TCGA, The Cancer Genome Atlas.

**Figure 2:**
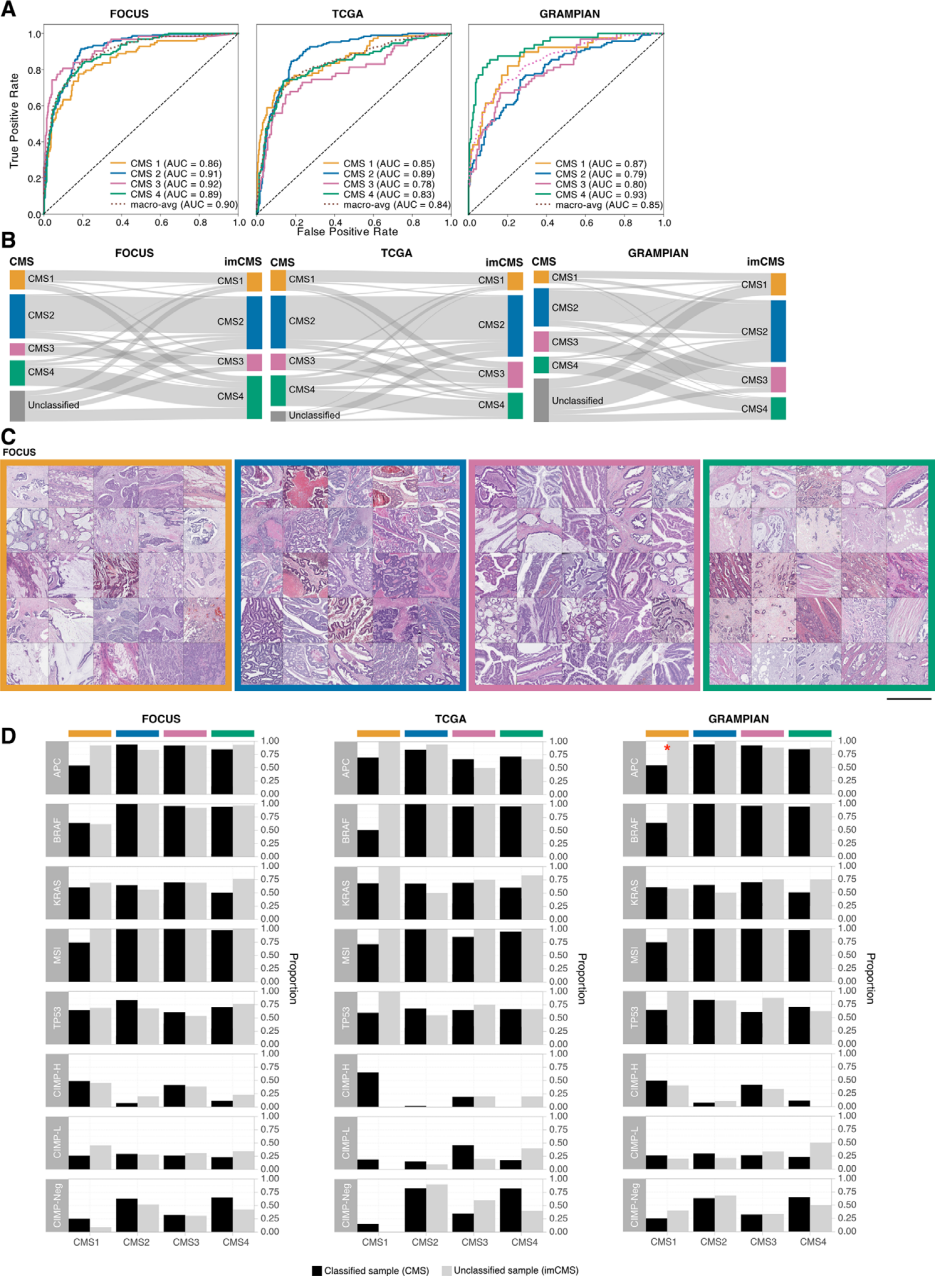
Image-based consensus molecular subtype (imCMS) classification. (A) Receiver operating curves of the imCMS classifier, optimised by the domain adversarial approach, on the FOCUS (n slides=510, 3×), TCGA (n slides=431, 3×) and GRAMPIAN cohorts (n slides=265, 12×). (B) Correspondences between CMS and imCMS classes in different datasets. All samples labelled as unclassified by RNA-based CMS calls were reclassified by imCMS. (C) Examples of image tiles with high prediction confidence for each imCMS class in FOCUS. Histological patterns associated with imCMS1 are mucin and lymphocytic infiltration. In imCMS2, evident cribriform growth patterns and comedo-like necrosis are observed, while imCMS3 is characterised by ectatic, mucin-filled glandular structures in combination with a minor component showing papillary and cribriform morphology. imCMS4 is predominantly associated with infiltrative CRC growth pattern, a prominent desmoplastic stromal reaction and frequent presence of single cell invasion (tumour budding). Scale bar ~1 mm. (D) Molecular associations of the CMS classified samples (black) and the CMS unclassified samples that have been classified by imCMS (grey). The molecular profiles of reclassified samples are largely consistent with those of the classified CMS samples. Statistically significant differences (p<0.05) are marked with a red asterisk. AUC, area under the curve; TCGA, The Cancer Genome Atlas.

CRC resection specimens (FOCUS, TCGA) and biopsies (GRAMPIAN) were used for algorithm training and testing. Samples from these cohorts have been prepared in separate institutions using different protocols and exhibit the expected range of morphological differences due to discrete preprocessing and preparation steps. The combination of these three specific cohorts captures sample variability as commonly encountered in pathology practice and provides an excellent opportunity to evaluate the generalisability of imCMS. Domain adversarial learning, a machine learning method that promotes the emergence of features that are discriminative for imCMS classification and indiscriminate of preanalytical variation between resection specimens,^21^ was used during model training. We leveraged 30% and 20% of the TCGA and GRAMPIAN cohorts in the domain adversarial training to discourage the learning of cohort-dependent features

(online supplementary materials and methods, online supplementary table S4); we further propose an adaptation of the deep learning model for colorectal biopsy samples. It should be noted that rectal biopsies have inherent biological differences with a lower frequency of CMS1 cases and a different representation of microenvironment features in biopsy samples. This resulted in very few biopsy samples classified as imCMS1 based on the majority vote rule (online supplementary table S5). To find the optimal cut-off for making an imCMS call, we used a small proportion of samples in the GRAMPIAN cohort to train a random forest (RF) classifier to make a final imCMS call based on the prediction score from the imCMS classifier (online supplementary materials and methods, online supplementary tables S5-S6). Although the domain adversarial training used parts of the TCGA and GRAMPIAN cohorts in the training, the improvement in the classification performance was consistent in both training and unseen parts of the TCGA and GRAMPIAN cohorts, alleviating the concern of overfitting (online supplementary table S7). Domain adversarial training improved classification accuracy of the final model to 0.9 AUC (70% average accuracy) in FOCUS (training), 0.84 AUC (64% average accuracy) in TCGA (test) and 0.85 AUC (72% average accuracy) in GRAMPIAN (test) (figure 2A, online supplementary figure S2; table 2, online supplementary table S8). The results presented hereafter are based on the imCMS classifier optimised by domain adversarial training. The correspondence of the CMS and imCMS classification calls for each case is shown in figure 2B. Next, we evaluated the consistency of the classification results on pairs of slides obtained from the same patients in the FOCUS and GRAMPIAN datasets. Two H&E slides were generated at different depth levels of each tissue block with at least four additional sections cut between for RNA extraction (online supplementary figure S3A). Since tissue features at different tissue levels are closely related, a robust classifier would be expected to achieve similar classification results. Indeed, imCMS classification scores were consistent between the slide pairs across different CMS classes (online supplementary figure S3B, online supplementary table S9).

10.1136/gutjnl-2019-319866.supp15 Supplementary data

10.1136/gutjnl-2019-319866.supp16 Supplementary data

10.1136/gutjnl-2019-319866.supp17 Supplementary data

10.1136/gutjnl-2019-319866.supp18 Supplementary data

10.1136/gutjnl-2019-319866.supp3 Supplementary data

10.1136/gutjnl-2019-319866.supp19 Supplementary data

10.1136/gutjnl-2019-319866.supp4 Supplementary data

10.1136/gutjnl-2019-319866.supp20 Supplementary data

**Table 2:** Area under the curve with 95% CIs achieved by the imCMS classifier trained by domain adversarial training

(A) FOCUS						
CMS	3× (n slides=510, n patients=278)					
	Model 1	Model 2	Model 3	Model 4	Model 5	Overall
CMS1	0.85 (0.77 to 0.96)	0.78 (0.64 to 0.93)	0.95 (0.91 to 1)	0.89 (0.82 to 0.97)	0.83 (0.72 to 0.97)	0.86 (0.81 to 0.92)
CMS2	0.9 (0.85 to 0.97)	0.96 (0.93 to 1)	0.93 (0.89 to 0.98)	0.9 (0.84 to 0.97)	0.87 (0.8 to 0.94)	0.91 (0.88 to 0.93)
CMS3	0.89 (0.81 to 1.02)	0.98 (0.97 to 1.01)	0.81 (0.67 to 1.01)	0.96 (0.92 to 0.99)	0.91 (0.86 to 0.99)	0.92 (0.88 to 0.98)
CMS4	0.89 (0.84 to 0.99)	0.9 (0.83 to 0.98)	0.88 (0.82 to 0.97)	0.89 (0.81 to 0.99)	0.91 (0.86 to 0.97)	0.89 (0.87 to 0.93)
Macro-average	0.88 (0.83 to 0.95)	0.9 (0.86 to 0.95)	0.89 (0.83 to 0.95)	0.91 (0.87 to 0.94)	0.88 (0.84 to 0.93)	0.9 (0.87 to 0.91)

(B) TCGA						
CMS	3× (n slides=431, n patients=430)					
	Model 1	Model 2	Model 3	Model 4	Model 5	Ensemble
CMS1	0.84 (0.79 to 0.91)	0.87 (0.83 to 0.92)	0.84 (0.78 to 0.91)	0.82 (0.77 to 0.89)	0.82 (0.75 to 0.88)	0.85 (0.8 to 0.9)
CMS2	0.88 (0.85 to 0.91)	0.87 (0.83 to 0.91)	0.87 (0.84 to 0.91)	0.83 (0.79 to 0.88)	0.83 (0.8 to 0.87)	0.89 (0.85 to 0.92)
CMS3	0.82 (0.76 to 0.88)	0.76 (0.7 to 0.82)	0.76 (0.7 to 0.83)	0.76 (0.67 to 0.82)	0.73 (0.66 to 0.81)	0.78 (0.71 to 0.85)
CMS4	0.84 (0.79 to 0.87)	0.84 (0.8 to 0.88)	0.8 (0.76 to 0.85)	0.81 (0.77 to 0.86)	0.8 (0.75 to 0.85)	0.83 (0.78 to 0.87)
Macro-average	0.84 (0.81 to 0.87)	0.83 (0.8 to 0.86)	0.82 (0.78 to 0.85)	0.8 (0.77 to 0.84)	0.79 (0.76 to 0.83)	0.84 (0.8 to 0.87)

(C) GRAMPIAN						
CMS	12× (n slides=265, n patients=144)					
	Model 1	Model 2	Model 3	Model 4	Model 5	Ensemble
CMS1	0.79 (0.71 to 0.87)	0.63 (0.51 to 0.74)	0.78 (0.66 to 0.87)	0.84 (0.77 to 0.93)	0.87 (0.82 to 0.95)	0.87 (0.81 to 0.94)
CMS2	0.83 (0.78 to 0.89)	0.73 (0.67 to 0.78)	0.73 (0.67 to 0.8)	0.75 (0.68 to 0.81)	0.77 (0.71 to 0.83)	0.79 (0.74 to 0.84)
CMS3	0.84 (0.78 to 0.89)	0.73 (0.65 to 0.81)	0.76 (0.68 to 0.83)	0.77 (0.72 to 0.83)	0.8 (0.74 to 0.87)	0.8 (0.75 to 0.86)
CMS4	0.92 (0.88 to 0.96)	0.84 (0.78 to 0.91)	0.87 (0.81 to 0.95)	0.92 (0.88 to 0.98)	0.94 (0.9 to 0.98)	0.93 (0.89 to 0.98)
Macro-average	0.84 (0.82 to 0.88)	0.73 (0.69 to 0.79)	0.78 (0.75 to 0.82)	0.82 (0.78 to 0.86)	0.85 (0.81 to 0.88)	0.85 (0.82 to 0.89)

CMS, consensus molecular subtype; TCGA, The Cancer Genome Atlas.

### Histological patterns associated with imCMS status

To understand which specific morphological patterns associate with imCMS, we extracted and visually reviewed tiles with the highest prediction confidence for each imCMS subtype. The large-scale histology patterns corresponded well with the biological characteristics of the CMS1 and CMS4 classes as predicted from the molecular assay^11^: mucinous differentiation and lymphocytic infiltration were associated with imCMS1, and a prominent desmoplastic stromal reaction with imCMS4. imCMS further allowed to visualise and systematically investigate tile level predictions for imCMS2 and imCMS3 with specific histological patterns. Image tiles associated with high-confidence calls of imCMS2 and imCMS3 showed a predominantly glandular differentiation (figure 2C, online supplementary figure 4A). In imCMS2, cribriform growth patterns and comedo-like necrosis were observed, while imCMS3 was characterised by ectatic, mucin-filled glandular structures in combination with a minor component showing papillary and cribriform morphology. Detailed visualisation of the image representations at the pixel-level^22^ corroborated the cellular and tissue components that weigh in on imCMS at high resolution (online supplementary figure S4B).

10.1136/gutjnl-2019-319866.supp5 Supplementary data

### imCMS classification of molecularly unclassified CMS samples

Failure of the transcriptional CMS classification might represent a transition phenotype, intratumoural heterogeneity or might represent technical failure to classify.^11^ We therefore tested the performance of imCMS in samples categorised as unclassifiable by transcriptomic CMS (figure 2B). Reclassification is underlined by direct comparison of the key molecular profiles between classified samples and the imCMS reclassified samples. No major differences between these two groups in the majority of the traits were found (figure 2D, online supplementary figure S5, online supplementary table S10).

10.1136/gutjnl-2019-319866.supp6 Supplementary data

10.1136/gutjnl-2019-319866.supp21 Supplementary data

### Spatial variation of imCMS class labels

**Figure 3:**
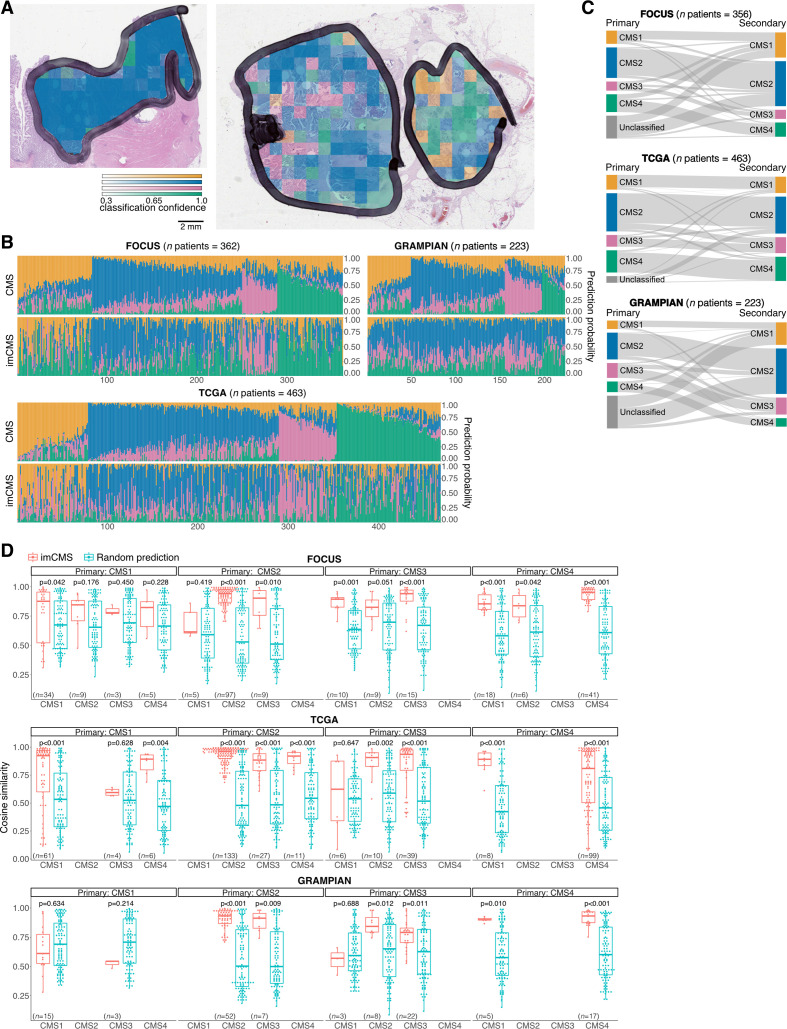
Intratumoural heterogeneity of the imCMS molecular subtypes. (A) Visualisation of the regional classification of the imCMS classifier. imCMS classification of a tumour sample can exhibit uniform results (left) or a degree of variation in the predicted imCMS class and the level of confidence (right). The colour overlay indicates the imCMS classes and the opacity reflects the classification confidence. (B) Heterogeneity of the CMS and imCMS classification scores. Each bar represents classification scores of a sample, and samples are sorted by the entropy of the prediction scores from the molecular-based random forest CMS classifier. (C) Heterogeneity of the CMS classification. A secondary CMS call was derived by relaxing the classification threshold of the random forest CMS classifier.^11^ (D) Cosine similarity between the imCMS and CMS prediction scores, stratified by the primary and secondary CMS calls. The levels of similarity were compared against those produced by a random classifier. Statistical analysis was performed using Wilcoxon rank-sum test, adjusted for the false discovery rate. P value <0.05 was considered statistically significant. *n* indicates the number of patients. Note that two diagnostic slides (serial sections) were available for the majority of cases in the FOCUS and GRAMPIAN cohorts. In cases where two slides were available, the analyses for each slide were performed separately. Panels (B) and (D) report the results for the first slide. The matched results for the second slide are provided in online supplementary figure S10. imCMS predictions represent the calls made by the domain adversarially trained imCMS classifier. imCMS, image-based consensus molecular subtype; TCGA, The Cancer Genome Atlas.

CRC tumours exhibit intratumoural variability in transcriptional features leading to a bias in transcriptional CMS calls introduced by the regions sampled for molecular analysis.^14^ imCMS captures this intrinsic variation in separate predictions for each image tile and provides a novel approach to investigate the intratumoural transcriptional heterogeneity of CRC (figure 3A, online supplementary figure S6A-D). We show that the distribution of the predicted imCMS labels is not random and that the morphological patterns associated with imCMS labels are consistent across cohorts (online supplementary materials and methods). We investigated if imCMS heterogeneity was associated with that of the molecular classification. Comparison of the imCMS versus CMS prediction scores revealed a high level of agreement between both classification schemes in the majority of the slides (figure 3B, online supplementary figure S7A). We next derived secondary CMS calls from the molecular data (figure 3C, online supplementary materials and methods) and further investigated the similarity between the corresponding CMS and imCMS prediction scores in groups stratified by primary and secondary CMS calls. Based on the cosine similarity measure, the concordance of the two prediction scores was significantly better than chance in a majority of the stratified groups (figure 3D, online supplementary figures S7B, S9, online supplementary materials and methods).

10.1136/gutjnl-2019-319866.supp7 Supplementary data

10.1136/gutjnl-2019-319866.supp8 Supplementary data

10.1136/gutjnl-2019-319866.supp10 Supplementary data

10.1136/gutjnl-2019-319866.supp11 Supplementary data

### Prognostic associations by imCMS status

**Figure 4:**
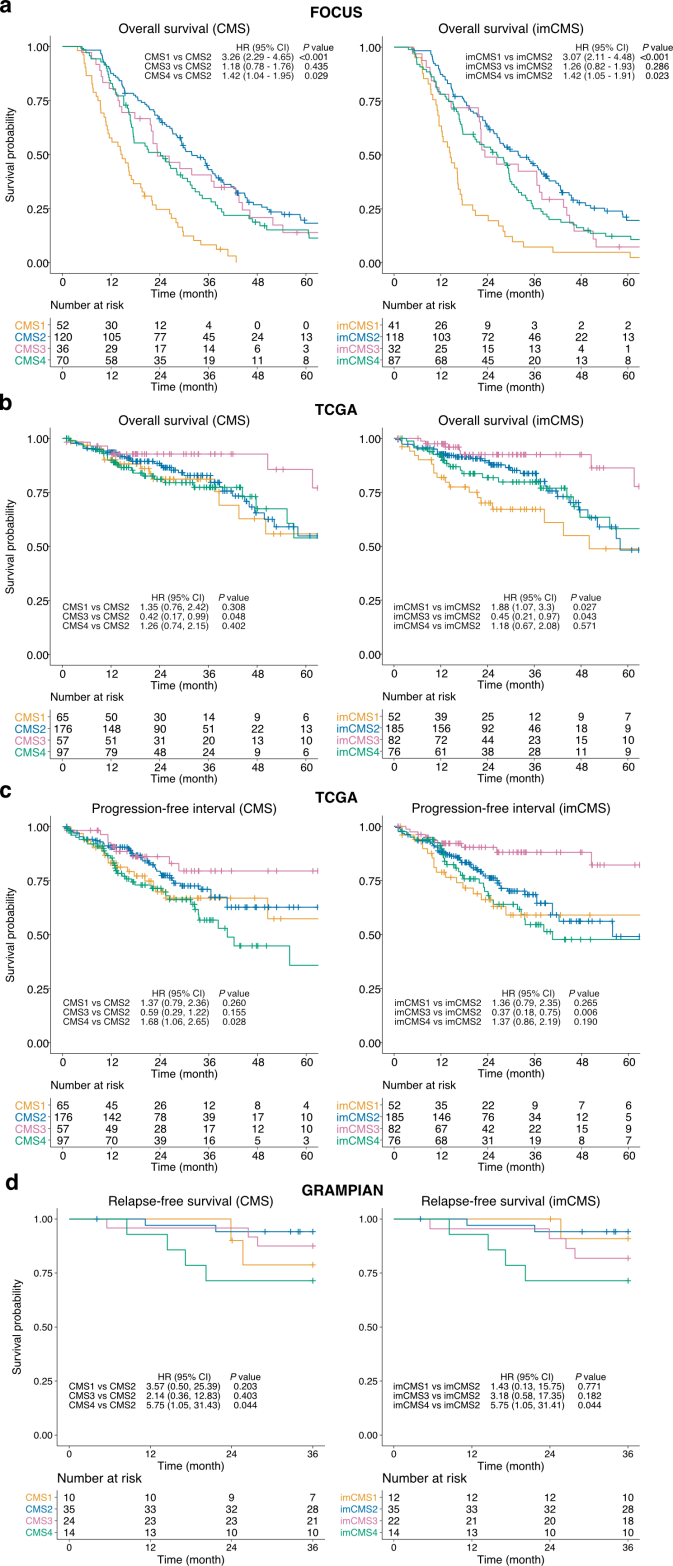
Prognostic associations of the image-based consensus molecular subtypes (imCMSs). Overall survival (OS) outcomes of the FOCUS cohort (n=278 patients, (A)) and TCGA cohort (n=395 patients, (B)), progression-free interval (PFI) outcome of the TCGA cohort (n=395, (C)) and relapse-free survival (RFS) outcome (n=83, (D)) as stratified by the transcriptional-based CMS classification and imCMS classification produced by the domain adversarially trained imCMS classifier. Kaplan–Meier estimator was used to estimate the survival probability, and pairwise log-rank test and univariate Cox proportional hazards regression were performed between CMS groups and imCMS groups. HRs and 95% CI for pairwise comparisons were reported. Test results with p value<0.05 were considered statistically significant. TCGA, The Cancer Genome Atlas.

We performed univariate Cox proportional hazard analysis to assess the prognostic associations of the imCMS classification compared with its molecular counterpart. The trend of patient survival outcomes stratified by imCMS was largely in agreement with those of the transcriptional classification (figure 4, online supplementary table S11) and imCMS survival predictions were concordant when the input slides were replaced by sections cut at deeper tissue levels in FOCUS and GRAMPIAN (online supplementary table S11, online supplementary figure S8). The prognostic association of the imCMS classification was maintained in multivariable analysis including TNM stage, age and gender, indicating strong potential to stratify risk beyond pathological staging (online supplementary table S11). In TCGA, a tendency towards worse overall survival was identified for cases classified as imCMS1 compared with molecular CMS in univariate analysis (imCMS HR=1.88, p value=0.027 vs CMS HR=1.35, p value=0.308). The same trend was captured in the multivariable analysis but was not statistically significant (imCMS HR=1.78, p=0.007 vs CMS HR=1.41, p=0.285). This discrepancy in the TCGA cohort may reflect the ability of the classifier to identify inherently poor prognosis CMS1 cases due to training on a cohort of metastatic CRC (FOCUS) and requires additional investigation in subsequent studies. TCGA cases classified as CMS4 showed a worse prognosis in univariate progression-free interval analysis (HR=1.68, p=0.028) while CMS1 associated with adverse outcome in multivariable analysis. Similar prognostic trends were reproduced by imCMS but did not reach statistical significance. In GRAMPIAN, both CMS and imCMS produced similar survival trends in both univariate and multivariate relapse-free survival analyses. Nevertheless, imCMS4 exhibits a stronger trend towards worse prognosis in the multivariate analysis compared with CMS4 (imCMS HR>6.63, p value<0.05 vs CMS HR=5.99, p value=0.061).

10.1136/gutjnl-2019-319866.supp22 Supplementary data

10.1136/gutjnl-2019-319866.supp9 Supplementary data

## Discussion

H&E slides are generated as part of the standard work-up of any CRC treated by surgical resection. In the assessment of this histologic material, pathologists are presently limited to a strictly defined set of morphologic and anatomic criteria.^23 24^ This information supports the definition of broad prognostic risk groups but has no predictive value.^24^ The integration of genomic technologies in the clinical care of patients with CRC has immense potential to drive personalised treatment and is now widely implemented for panel-based DNA analysis. While this is of great value in some tumour types such as non-small cell lung cancer, in CRC and many other tumour types the impact is relatively minor. Gene expression data provide information regarding the behaviour of epithelial, stromal and immune compartments of the tumour, which is more informative especially in CRC as the basis for the CMS subtyping, but requires substantial financial, personnel and infrastructure resources.^1^ Combining morphological and molecular pathology is a promising approach to extend the amount of clinically relevant information that can be extracted from standard histologic slides.^8 25^ In this study, we leverage advanced machine learning for the development of an image-based taxonomy of CRC that was previously inaccessible to human interpretation.

We trained and tested the imCMS approach towards consensus molecular subtyping of CRC on three independent and well-characterised patient cohorts with availability of digital slides and transcriptional information from the MRC CRUK S:CORT consortium and TCGA. We specifically focused on relevant clinical scenarios in the management of patients with CRC and investigated the imCMS classification of both preoperative biopsies and resection specimens. Our analysis demonstrates the feasibility of imCMS classification of both primary colon and rectal resection specimens in the FOCUS and TCGA cohorts. imCMS calls closely matched transcriptional classification for survival stratification, underlining the potential of imCMS to aid pathologists in the identification of aggressive disease for intensified follow-up and chemotherapy trials.^1^ In advanced disease, the development of molecular stratifiers for the prediction of treatment response is of critical importance to balance care and overtreatment. No clinically approved tests are currently available to predict chemotherapy response in metastatic CRC and beneficial effects are set-off by considerable toxicity.^26 27^ Transcriptional classification of CRC has shown promise to stratify survival outcomes and response to treatment in retrospective analyses but requires further validation.^26 28 29^ imCMS represents a readily translatable and cost-effective approach for further investigation of treatment outcomes in existing retrospective cohorts with potential to inform future clinical trials.

Small biopsy fragments have previously proven difficult to analyse using genomic technologies due to the limited amount of tissue available and pathologist assessment is usually restricted to the diagnosis of cancer, a select panel of immunohistochemical studies and a limited assessment of additional prognostic features.^27 30^ We found that imCMS could be efficiently adapted for morpho-molecular classification of rectal cancer biopsy fragments at high magnification. Further study where the model is trained on biopsy samples and evaluated on independent biopsy cohorts is required. Clinically approved assays that are predictive of therapeutic response from biopsy material are presently lacking, with up to 25% of patients with rectal cancer gaining no benefit from current radiotherapy and chemotherapy protocols.^29^ As a stemlike (CMS4) transcriptional profile of CRC has been linked to poor prognosis and therapeutic resistance, imCMS could allow for more effective stratification of patients for prospective studies in primary surgery or neoadjuvant treatment.^31^

The robustness of image analysis algorithms is a well-recognised problem in the setting of limited training sets and poorly annotated reference data. We addressed the problem of sample diversity by training the imCMS classifier on histological samples sourced from multiple institutions (n=59) participating in the FOCUS trial. We also included endoscopic biopsies and surgical resections as two diverse examples of CRC tissue specimens with different preprocessing procedures. Biopsy tissue undergoes relevant compression stress during capture by endoscopic forceps leading to architectural distortion and variable alteration of cellular morphology. We show that imCMS classification is relatively stable across these settings. Domain adversarial training was used to further minimise learning features that are attributed to a specific cohort.^21^ Indeed, an ensemble of multiple models, analogous to consensus of experts’ opinions, reduces the bias of individual predictions.^21^ Our study underlines that convolutional neural networks excel in their ability to learn relationships of tissue compartments as a whole and to identify relevant patterns with clear morphological interpretability. The resulting feature space represented both tumour-intrinsic and microenvironment-related signals, which are intimately linked to CRC phenotypes with distinct biological characteristics and disease outcomes. Indeed, imCMS highlighted the well-known morpho-molecular associations with inflammatory infiltrates (imCMS1) and a prominent stromal reaction (imCMS4) but also identified novel morphological features in association with high-confidence calls of imCMS2 and imCMS3. Despite these promising results, potential overfitting of the computational model to the training cohort is a limitation of the current study, and results need to be interpreted in the context of the current stage of research development.

Transcriptomic CMS was released as the most robust molecular classification in CRC and the basis for clinical stratification and targeted intervention.^1 11^ However, some key issues hamper clinical implementation of CMS such as the inability to obtain reliable calls from single samples. Two methods to call CMS were released by the original authors based on RF and single sample prediction.^1 11^ RF classification is cohort-dependent and requires a high minimum number of samples, while calls on single samples are often of limited quality leading to underutilisation. Some samples do not show enough evidence to make calls by either method leading to a substantial number of cases left as unclassified. Inconsistent classification calls could also be an expression of intratumoural heterogeneity or representative of a transition phenotype, which is of considerable biological interest.^1^ imCMS is able to overcome all these problems. First, imCMS calls are intrinsically generated for single samples. Second, imCMS classification visualises heterogeneity through tile-based calls with a cell size of 512×512 pixels, leading to quantitative prediction scores while retaining the value of an image to support understanding at a pathomorphological level. Importantly, all CMS unclassified samples were reclassified by imCMS, with their molecular characteristics closely resembling those classified by sequencing methods. Our results suggest that imCMS performs reliably in samples categorised as unclassified by transcriptional profiling. Nonetheless, this should not be interpreted that the stratification of unclassified samples into molecular subgroups is considered correct. Rather, imCMS provides a tile-based overlay of CMS subtypes across a single section, thereby providing rich information about tissue heterogeneity and spatial context readily accessible to the reporting pathologist. imCMS is a versatile tool to address deficiencies in transcriptional profiling that may arise due to low amounts or quality of RNA, an expected problem in clinical formalin-fixed paraffin-embedded blocks. Importantly, it offers a possible alternative solution when there is an inability to confidently call CMS in single samples from transcriptomic data.

Tumour heterogeneity introduces important challenges in designing optimal treatment strategies for patients with cancer.^14 30^ To investigate sample heterogeneity, we bioinformatically derived secondary CMS calls from all samples and investigated the similarity of the CMS and imCMS prediction scores for primary and secondary calls. imCMS captured secondary calls with high accuracy based on a cosine similarity measure between transcriptional and image-based classification. Hallmark of imCMS is the ability to predict a class label for a given image tile and hence the ability to estimate a distribution of labels in a given tumour area. We provide evidence that the distribution of imCMS labels is not random, it is consistent across the different cohorts, and the reinterpretation of RNA expression is in line with local imCMS label predictions. Further investigation of local tumour heterogeneity using methods such as spatial transcriptomics is warranted.

Prospective validation of imCMS in independent studies will be critical to clinical translation. A first application will be its use as a tool to call CMS in large clinical trial cohorts in whom funding for gene expression profiling is not available. This will build the evidence base to show whether or not imCMS can be used as a predictive biomarker for treatment response or patient enrichment for certain research studies. We hypothesise that the general principle can be applied not only to other cancer types but also to other diseases. It will therefore lay the foundation of a more systematic integration of image-based morphological analysis and molecular stratification.
