# Supplementary material for: Image-based consensus molecular subtype (imCMS) classification of colorectal cancer using deep learning
Source: Gut. 2020 Jul 20;70(3):544–54. doi: 10.1136/gutjnl-2019-319866 (PMC7873419; doi:10.1136/gutjnl-2019-319866)
Supplement: Supplementary data [file gutjnl-2019-319866supp001.pdf]

Title: Image-based consensus molecular subtype classification (imCMS) of colorectal cancer using deep learning

Short title: Image-based classification of colorectal cancer

Authors: Korsuk Sirinukunwattana[1,2,3], Enric Domingo[4*], Susan Richman[5], Keara L Redmond[6], Andrew Blake[4], Clare Verrill[3,7,8], Simon J Leedham[9,10], Aikaterini Chatzipli[11], Claire Hardy[11], Celina Whalley[12], Chieh-Hsi Wu[13], Andrew D Beggs[12], Ultan McDermott[11], Philip Dunne[6], Angela A Meade[14], Steven M Walker[6,15], Graeme I Murray[16], Leslie M Samuel[17], Matthew Seymour[5], Ian Tomlinson[12], Philip Quirke[5], Tim Maughan[18], Jens Rittscher[1,2,3,19§*] and Viktor H Koelzer[4,20,21§*] on behalf of S:CORT consortium

Affiliations:

[1] Institute of Biomedical Engineering (IBME), Department of Engineering Science, Old Road Campus Research Building, University of Oxford, Oxford, UK

[2] Big Data Institute, University of Oxford, Li Ka Shing Centre for Health Information and Discovery, Oxford, UK

[3] Oxford NIHR Biomedical Research Centre, Oxford University Hospitals Trust, Oxford, UK

[4] Department of Oncology, University of Oxford, Oxford, UK

[5] Department of Pathology and Tumour Biology, Leeds Institute of Cancer and Pathology, Leeds, UK

[6] Centre for Cancer Research & Cell Biology, Queens University, Belfast, UK

[7] Department of Cellular Pathology, Oxford University Hospitals NHS Foundation Trust, Oxford, UK

[8] Nuffield Department of Surgical Sciences and NIHR Oxford Biomedical Research Centre, University of Oxford, Oxford, UK

[9] Gastrointestinal Stem-cell Biology Laboratory, Oxford Centre for Cancer Gene Research, Wellcome Trust Centre for Human Genetics, University of Oxford, Oxford, UK.

[10] Translational Gastroenterology Unit, Experimental Medicine Division, Nuffield Department of Clinical Medicine, John Radcliffe Hospital, Oxford, UK.

[11] Wellcome Trust Sanger Institute, Hinxton, UK

[12] Institute of Cancer and Genomic Science, University of Birmingham, UK

[13] Department of Statistics, University of Oxford, Oxford, United Kingdom.

[14] MRC Clinical Trials Unit at University College London, London, UK

[15] Almac Diagnostics, Craigavon, UK

[16] Department of Pathology, School of Medicine, Medical Sciences and Nutrition, University of Aberdeen, Aberdeen, UK

[17] Department of Clinical Oncology, Aberdeen Royal Infirmary, NHS Grampian, Aberdeen, UK

[18] CRUK/MRC Oxford Institute for Radiation Oncology, University of Oxford, Oxford, UK

[19] Ludwig Institute for Cancer Research, University of Oxford, Nuffield Department of Medicine, Old Road Campus Research Building, Oxford, UK

[20] Nuffield Department of Medicine, University of Oxford, Oxford, UK

[21] Department of Pathology and Molecular Pathology, University Hospital and University of Zurich, CH-8091 Zurich, Switzerland

§ These authors jointly directed this work: Jens Rittscher and Viktor H Koelzer

Sirinukunwattana K, *et al. Gut* 2021; 70:544–554. doi: 10.1136/gutjnl-2019-319866

SUPPLEMENTARY MATERIALS AND METHODS

Patients

Cohort 1: FOCUS (Retrospective cohort, S:CORT)

As part of the Stratification in COloRecTal cancer (S:CORT) program, 375 patients with available formalin-fixed paraffin embedded (FFPE) blocks of the primary CRC were selected from the MRC FOCUS randomised clinical trial (RCT) that tested different strategies of sequential and combination chemotherapy for patients with advanced CRC.[1] Serial sections were cut from one representative block for H&E staining followed by four unstained sections for RNA extraction, a second H&E and eight unstained sections for DNA extraction for a total of 722 slides. Glass H&E slides were re-reviewed by an expert gastrointestinal pathologist and tumour tissue with the associated intratumoural stroma was annotated and used to guide RNA and DNA extractions from the first and second H&E respectively. No tumour microdissection was performed. Regions of extensive necrosis and non-tumour tissue were excluded according to standard practice for downstream molecular tumour profiling. RNA expression microarrays (Xcel array, Affymetrix), DNA target capture (SureSelect, Agilent) followed by NGS sequencing (Illumina) and DNA methylation arrays (EPIC arrays, Illumina) were applied in this order. All H&E slides were scanned at high resolution on an Aperio scanner at a total magnification of 20x. Digital slides were re-reviewed by a second gastrointestinal pathologist and tumour annotations were traced to generate region annotations for machine learning classification. Areas containing folds or debris were excluded by digital annotation. Clinical data was retrieved from the trial database. Pathological TNM-stage and sidedness were extracted from pathological reports. Patients with synchronous disease were considered to be stage IV. 56 slides with technical failure of the staining or scanning procedure were excluded from further analysis for a final set of 666 slides (n=362 cases). Clinical and molecular data is summarised in **[Table S1]** and **[Figure 1A-B]**.

Cohort 2: TCGA (colon and rectal adenocarcinomas)

A total of 624 digital slides from 615 cases of colon and rectal adenocarcinoma with available FFPE samples were downloaded from the TCGA Data Portal (data accessed on August 2nd, 2018). All

Sirinukunwattana K, *et al. Gut* 2021; 70:544–554. doi: 10.1136/gutjnl-2019-319866

digital slides were re-reviewed and tumour tissue was annotated. A total of 156 slides were excluded (42 based on quality control criteria and 114 had no CMS classification as explained below). Clinical data was obtained from Liu et al.[2] while somatic mutations and gene level expression data were downloaded with the R package TCGAbiolinks[3]on November 7th, 2018. Mutations from Varscan and Mutect were combined and calls for driver mutations were computed for relevant genes (all truncating mutations for *APC*; missense mutations for *KRAS* in codons 12, 13, 19, 22, 59, 61, 68, 117 and 146; V600E for *BRAF;* all missense and truncating mutations for *TP53*). The final number of slides for imCMS classification was 468 (n = 463 patients) **[Table S1]** and **[Figure 1A-B]**.

Cohort 3: GRAMPIAN (Retrospective cohort, S:CORT)

A total of 418 slides from 225 pre-treatment biopsy FFPE blocks from rectal cancer patients of the neoadjuvant setting were available for this study as part of the S:CORT program. All patients received pre-operative chemoradiotherapy followed by surgical resection. Slides and molecular profiling were processed as described for cohort 1 (FOCUS) but using 5 to 9 sections for RNA extraction and 9 for DNA. Staging was derived at time of resection after neoadjuvant treatment. A total of 12 slides were excluded based on quality control criteria for a final set of 406 slides (n = 223 cases). Clinical and molecular data is summarised in **[Table S1]** and **[Figure 1A-B]**.

CMS calls

CMS were derived with the random forest (RF) CMSclassifier with the default posterior probability of 0.5. RF CMS classification of FFPE samples from the FOCUS and GRAMPIAN cohorts led to an increased frequency of unclassified samples as compared to the TCGA datasets derived from fresh frozen material. To derive calls with comparable frequencies, we computed single sample predictor calls after row-centring the expression data.[4]Final CMS calls were generated when there was a match between both methods (RF and single sample predictor without applying any cut-off). There were 110 TCGA cases with discrepancies between our CMS calls and the calls originally reported, mostly involving a lost or gain of unclassified status.[4] These discrepant calls are most likely due to application of a clustering method that is strongly cohort-dependent in our analysis of the TCGA

Sirinukunwattana K, *et al. Gut* 2021; 70:544–554. doi: 10.1136/gutjnl-2019-319866

samples while the original report combines thousands of samples from several cohorts. Due to lack of clear evidence of the ground truth CMS status, these samples were excluded from analysis.

Secondary CMS calls from RNA in classified samples were computed by RF using the second highest call with posterior probability above 0.3. The primary call was matched if no different CMS subtype was found. For unclassified samples, the first highest call above 0.3 was used, leaving the sample as unclassified if no subtype met this requirement. All these analyses were performed with R v3.5.1.

imCMS classification

Pre-processing of image data and exclusion criteria

Digital slides were re-reviewed and invasive cancer regions were annotated by an expert gastrointestinal pathologist using the HALO™ software v2.3.2089.52 (Indica Labs, Corrales, NM, USA). For each slide, the annotated tumour areas were divided into tiles of 512x512 pixels. Tiles that have an overlap with the annotated tumour areas of less than 20% were excluded to avoid tiles containing pen markers. Tiles with less than 50% tissue area were further excluded. We identify tissue areas by converting an image into greyscale [5] and using Otsu's method [6] to determine a global threshold in which pixels with a greyscale value higher than the threshold were considered background and those with a greyscale value lower than or equal to the threshold were considered part of tissue areas. Total tissue area and the number of tiles is shown in **[Figure S1]**. At 5x magnification, neighbouring tiles were 50% overlapped in the FOCUS and TCGA cohorts (resections). To account for the small sample surface area of the tumour identified in endoscopic biopsies of the GRAMPIAN cohort at 5x, tiles with a 75% overlap were used. At 20x, no overlap in FOCUS and TCGA and 50% overlap in GRAMPIAN were used.

imCMS classifier and the training procedure

We trained a neural network to classify a given image tile taken from the marked tumour area into one of the 4 CMS classes. An Inception V3[7] model whose final fully connected layer was modified to output 4 classes was initialised with weights pretrained on the ImageNet dataset[8]. The network was trained on samples taken from the FOCUS cohort **[Figure 1C, Table S4].** The class of each tile

in the training set was matched to the overall RNA-based CMS call of the FOCUS slide. Image tiles were resized from 512x512 pixels to 299x299 pixels before feeding to the network, effectively reducing the magnification of tiles from 5x to 3x and 20x to 12x. Tiles from unclassified slides were excluded. We trained 5 separate models with different subsets of the data in the manner akin to cross-validation **[Table S4]**. That is three portions were used for training, and one portion each for validation and testing. During training, classification performance at the slide level, determined by a macro average F1 score, was evaluated on the validation partition. The model that yielded the highest performance on this portion was then selected and applied on the test portion as well as unseen samples from TCGA and GRAMPIAN cohorts **[Table S4]**. The split was done at the patient level, i.e., no image tiles from the same patients would be used for training, validation, and testing at the same time. During training, data augmentation [9] were applied in the following order: (1) random horizontal flip with 50% chance and random vertical flip with 20% chance, (2) random orientation of image tiles with a rotation degree uniformly drawn from a set of [0, 90, 180, and 270], (3) colour jittering with brightness, contrast, saturation, and hue factors uniformly drawn from the intervals [0.5, 1.5], [0.75, 1.5], [0.8, 1.2], and [-0.1, 0.1], respectively. Data augmentation, including colour jittering, random flip and random orientation of image tiles were used during training. We set the initial learning rate at 0.0002 and used ADAM optimiser[10].

Testing the model on independent cohorts

On the TCGA and GRAMPIAN datasets, we applied 5 versions of the imCMS model, producing 5 different classification results for each tile which were then averaged to obtain the final prediction **[Table S4]**. The prediction probability for each imCMS class was obtained from the proportion of the number of tiles assigned to that class, and the final imCMS call at the slide level was derived from the majority vote of tiles **[Figure 1D]**. No unclassified slides were used in the evaluation. The classification performance of the model is reported in **[Table S2, Table S3]**.

Determination of an optimal decision cutoff in the GRAMPIAN cohort

Image tiles containing features associated with the imCMS1 class in resection specimens were underrepresented in the rectal biopsies in the GRAMPIAN cohort due to the inherent biological differences in rectal cancer with a lower frequency of CMS1 cases as well as the clinical process of biopsy sampling from the tumour surface, leading to a different representation of microenvironment features in biopsy samples. This resulted in very few image tiles classified as imCMS1 **[Table S5]**, leading to very few biopsy samples classified as imCMS1 based on the majority vote rule **[Table S3].** As such, an alternative decision cut-off that took into account the underrepresentation of imCMS1 tiles was required. To this end, we trained a RF model with 100 trees of the maximum depth of 2. The input representing each biopsy slide was a vector of the imCMS prediction scores and the output was the predicted imCMS class. We trained the model on the 20% of CMS classified patients (the same subset of the data used in the domain adversarial training described in the below section). In addition, we showed that the choice of the training subset did not compromise the performance of the random forest model **[Table S6]**. Here, we performed 3 separate trials in which 20% of CMS classified patients were selected at random while preserving the class distribution of the original data to train the model.

Domain adversarial training for better generalisation

To prevent the learning of dataset-dependent features that would limit the general applicability of the model, we leveraged domain-adversarial training.[11] The model was augmented with an additional classifier for predicting whether tiles were drawn from the training (FOCUS) or external cohorts (TCGA and GRAMPIAN) **[Figure 1C]**. We forced this classifier to perform poorly to encourage the learning of dataset-independent features. Domain adversarial training did not involve imCMS class information. From all cohorts a random subset of tiles taken from CMS classified samples was used during training. All CMS labelled cases of FOCUS were used, 30% of the CMS labelled patient cases of TCGA as well as 20% of the CMS labelled cases from GRAMPIAN cohorts. **[Table S4].** Patient cases from TCGA and GRAMPIAN were selected randomly. This random selection was only performed once and the resulting adversarial learning is based on this

specific selection. While using a small proportion of samples from TCGA and GRAMPIAN for the domain adversarial training might introduce a bias **[Figure 2a, Tables 2, S8]**, the improvement was consistent in both training and unseen portions of the TCGA and GRAMPIAN data **[Table S7]**. This supports the argument that domain adversarial learning was critical to train a classifier that is suitable for a better generalisation.

Handling of multiple slides in patient-level analyses

Two serial sections were available for the majority of cases in FOCUS and GRAMPIAN cohorts (see Patient Section above for details). To avoid data correlation due to multiple representative slides from the same patients, we performed two separate analyses. If a case consisted of two slides, only one slide was used in each analysis. If only one slide was available, it would be used in both analyses. This applies to the following analyses: i) molecular association of the CMS unclassified samples **[Figures 2D, S5, Table S10]**, ii) cosine similarity between the imCMS and CMS prediction scores **[Figures 3D, S7B],** and iii) Survival analyses **[Figures 4, S8, Table S11]**

Intratumoural heterogeneity of the imCMS classification

We approached the problem of tumour heterogeneity using three different modelling approaches: First, to assess if the local distribution of imCMS is not due to random chance, we statistically compared the distribution of imCMS calls generated by the classifier with calls generated by a model assigning each CMS class an equal weight using the Kolmogorov-Smirnov test (H0: imCMS labels are uniformly distributed; H1: imCMS labels are not uniformly distributed). The results are shown in **[Figure S5]**. Second, we assessed the robustness of imCMS across the three different cohorts. Here, we represented each of the imCMS classes as a distribution over morphological patterns, which are identified using clustering methods (see details in **Clustering Analyses** below). We observed that the distribution profiles of the same CMS class are consistent across cohorts. This implies that our classification framework can robustly identify common morphological patterns that are enriched in different transcriptional classes **[See Figure S10]**. Third, we assessed whether the similarity between the imCMS and CMS prediction probabilities is not due to random chance. Cosine similarity was calculated between each pair of corresponding imCMS and CMS prediction probabilities. Samples

Sirinukunwattana K, *et al. Gut* 2021; 70:544–554. doi: 10.1136/gutjnl-2019-319866

were then stratified according to their primary and secondary CMS profile. For each stratification, we tested the null hypothesis (H0) that the distribution of the cosine similarity values between pairs of corresponding imCMS and CMS prediction probabilities is similar to the distribution of the cosine similarity values calculated between pairs of CMS and random prediction probabilities. Here, we assumed that a random model, in which the probability for all imCMS classes are equal, is a 4-dimensional Dirichlet distribution with a concentration parameter of 1.0 in each dimension. To form a baseline distribution in each stratification of samples, a total of 100 random prediction probabilities were drawn and the cosine similarities of these random prediction probabilities and the mean of the CMS prediction probabilities were calculated. To test the null hypothesis, the median difference between groups was compared using the Wilcoxon rank-sum test. The p-values were adjusted to control false discovery rate.[12] Any comparison that was highly underpowered due to the sample size (less than 2 data points in one of the populations) was discarded. For each group, outliers were removed using Tukey's rule.[13] P-values <0.05 were considered statistically significant. See comparison results in **Figures 3 and S7**.

Clustering analyses

We performed clustering analysis on image tiles to identify differential histological patterns **[Figure S10]**. We represented each image tile by a feature vector obtained from the convolutional layer prior to the fully connected layer in the Inception V3 model. The feature vectors were standardised independently in each dimension to have zero mean and unit variance. Principal component analysis (PCA) was then performed to reduce the dimensionality of the feature vectors from 1024 to 128. The choice of the reduced dimensionality was based on the amount of explained variance.

In the clustering analysis, we sampled up to 20 tiles from each slide from all 3 datasets, resulting in a total of 27,053 tiles. Self-organising map (SOM)[14] with a 10-by-10 grid was then performed on the feature vectors representing these tiles to initially identify 100 clusters of tiles with histologically similar patterns. Consensus clustering[15] based on robust continuous clustering (RCC)[16] was

Sirinukunwattana K, *et al. Gut* 2021; 70:544–554. doi: 10.1136/gutjnl-2019-319866

subsequently deployed to determine the final similarity clusters. Here, the consensus clustering was done on the set of 100 SOM exemplars and for the RCC we used the cosine distance as a similarity metric and set the clustering threshold to 1. In the bootstrapping process of the consensus clustering, the number of neighbours (*k*) of the RCC was varied from 3 to 11, and for each value of *k*, we ran RCC for 50 times each with 80% of the exemplars.

Survival analyses

Overall survival (OS) in the FOCUS cohort was computed from the time of diagnosis of the primary CRC (from 1988 to 2003) until death and was right censored for patients still alive at the date of last known follow-up. Progression-free interval (PFI) in the TCGA cohort was defined as a period from the date of diagnosis until the date of the first occurrence of a new tumour event, which includes progression of the disease, locoregional recurrence, distant metastasis, new primary tumour, or death with tumour. Patients who were alive without these events or died with unrelated reasons were censored[2]. OS and PFI in TCGA were retrieved from Liu et al[2]. Relapse-free survival (RFS) was measured in GRAMPIAN and right-truncated at 3 years. RFS is a period from the date of first diagnosis until the date of the first relapse event after confirmation of a disease-free status. Patients who died with unrelated reasons or were alive without new relapse events were censored [2]. In total, survival data were available in 362, 460, and 125 patients from FOCUS, TCGA and GRAMPIAN cohorts, respectively **[Table S1]**. In all cohorts, CMS unclassified patients were excluded. This excluded 84 (FOCUS), 33 (TCGA), and 42 (GRAMPIAN) patients. A total of 32 patients with less than 1 month follow-up time were further excluded from the TCGA cohort. This led to 278 (FOCUS), 395 (TCGA), and 83 (GRAMPIAN) patients used in the analyses. Univariable and multivariate Cox proportional hazards analyses were performed to assess the prognosis. Multivariate Cox regression analysis was carried out with TNM stage, age and gender as possible confounding factors. Patients with missing data in any covariate were dropped from the models. P-values <0.05 were considered statistically significant. Results are summarised in **Table S11.**

Sirinukunwattana K, *et al. Gut* 2021; 70:544–554. doi: 10.1136/gutjnl-2019-319866

Ethics approval

Cohorts 1 and 3 of S:CORT had ethical approval (REC 15/EE/0241) by the East of England - Cambridge South Research Ethics Committee.

Sirinukunwattana K, *et al. Gut* 2021; 70:544–554. doi: 10.1136/gutjnl-2019-319866

REFERENCES

1 Seymour MT, Maughan TS, Ledermann JA, Topham C, James R, Gwyther SJ*, et al.* Different strategies of sequential and combination chemotherapy for patients with poor prognosis advanced colorectal cancer (MRC FOCUS): a randomised controlled trial. Lancet 2007;**370**:143-52.

2 Liu J, Lichtenberg T, Hoadley KA, Poisson LM, Lazar AJ, Cherniack AD*, et al.* An Integrated TCGA Pan-Cancer Clinical Data Resource to Drive High-Quality Survival Outcome Analytics. Cell 2018;**173**:400-16 e11.

3 Colaprico A, Silva TC, Olsen C, Garofano L, Cava C, Garolini D*, et al.* TCGAbiolinks: an R/Bioconductor package for integrative analysis of TCGA data. Nucleic Acids Res 2016;**44**:e71.

4 Dienstmann R, Vermeulen L, Guinney J, Kopetz S, Tejpar S, Tabernero J. Consensus molecular subtypes and the evolution of precision medicine in colorectal cancer. Nature Reviews Cancer 2017;**17**:79.

5 BT RI-R. Studio encoding parameters of digital television for standard 4: 3 and wide-screen 16: 9 aspect ratios. International Telecommunication Union Standard Geneva CH (2011).

6 Otsu N. A threshold selection method from gray-level histograms. IEEE transactions on systems, man, and cybernetics:62-6.

7 Szegedy C, Vanhoucke V, Ioffe S, Shlens J, Wojna Z. Rethinking the inception architecture for computer vision. 2016:2818-26.

8 Russakovsky O, Deng J, Su H, Krause J, Satheesh S, Ma S*, et al.* ImageNet Large Scale Visual Recognition Challenge. International journal of computer vision 2015 **115**:211-52.

9 Paszke A, Gross S, Chintala S, Chanan G, Yang E, DeVito Z*, et al.* Automatic differentiation in PyTorch. NIPS-W 2017.

10 Kingma DP, Ba J. Adam: A method for stochastic optimization. a. rXiv preprint arXiv:14126980 2014.

11 Ganin Y, Ustinova E, Ajakan H, Germain P, Larochelle H, Laviolette F*, et al.* Domain-adversarial training of neural networks. The Journal of Machine Learning Research 2016;**17**:2096-30.

12 Benjamini Y, Hochberg Y. Controlling the false discovery rate: a practical and powerful approach to multiple testing. Journal of the Royal statistical society: series B (Methodological) 1995:289-300.

13 Beyer H, Tukey JW. Exploratory Data Analysis. . Addison-Wesley Publishing Company Reading, Mass—Menlo Park, Cal, London, Amsterdam, Don Mills, Ontario, Sydney 1977, XVI, 688 S.

14 Kohonen T. Exploration of Very Large Databases by Self-Organizing Maps. . International Conference on Neural Networks (ICNN 97), Houston, Texas, USA 1997.

15 Monti S, Tamayo P, Mesirov J, Golub T. Consensus Clustering: A Resampling-Based Method for Class Discovery and Visualization of Gene Expression Microarray Data. Machine Learning 2003;**52**:91-118.

16 Shah SA, Koltun V. Robust continuous clustering. Proc Natl Acad Sci U S A 2017;**114**:9814-9.

Sirinukunwattana K, *et al. Gut* 2021; 70:544–554. doi: 10.1136/gutjnl-2019-319866
