# Supplementary material for: Image-based consensus molecular subtype (imCMS) classification of colorectal cancer using deep learning
Source: Gut. 2020 Jul 20;70(3):544–54. doi: 10.1136/gutjnl-2019-319866 (PMC7873419; doi:10.1136/gutjnl-2019-319866)
Supplement: Supplementary data [file gutjnl-2019-319866supp012.pdf]

Table S01

FOCUS		All samples	CMS1	CMS2	CMS3	CMS4	Unclassified
**Clinico-pathological features**		Statistics	Statistics	Statistics	Statistics	Statistics	Statistics
Age		*n=362*	*n=52*	*n=120*	*n=36*	*n=70*	*n=84*
	Median (range)	64 (31, 83)	60 (35, 82)	66 (39, 83)	65 (48, 79)	63 (32, 82)	65 (31, 81)
Gender		*n=362*	*n=52*	*n=120*	*n=36*	*n=70*	*n=84*
	Male	229	29	79	22	42	57
	Female	133	23	41	14	28	27
Site		*n=326*	*n=45*	*n=106*	*n=33*	*n=64*	*n=78*
	Right colon	130	27	28	17	21	37
	Left colon	196	18	78	16	43	41
TNM Stage		*n=350*	*n=50*	*n=116*	*n=35*	*n=66*	*n=83*
	I	4	0	3	0	0	1
	II	39	2	22	6	5	4
	III	75	6	25	8	19	17
	IV	232	42	66	21	42	61
Overall survival (months)		*n=362*	*n=52*	*n=120*	*n=36*	*n=70*	*n=84*
	Median (range)	23 (2, 172)	15 (4, 43)	30 (5, 119)	23 (5, 172)	23 (5, 107)	20 (2, 91)
**Major molecular features**							
Microsatellite status		*n=339*	*n=47*	*n=116*	*n=30*	*n=65*	*n=81*
	Stable (MSS)	328	37	116	30	64	81
	Instable (MSI)	11	10	0	0	1	0
CIMP		*n=313*	*n=41*	*n=108*	*n=28*	*n=61*	*n=75*
	CIMP.High	72	22	7	13	9	21
	CIMP.Low	83	11	29	5	13	25
	CIMP.Negative	158	8	72	10	39	29
**Selected mutations**							
KRAS mut status		*n=339*	*n=47*	*n=116*	*n=30*	*n=65*	*n=81*
	Wild-type	171	31	68	8	33	31
	Mutated	168	16	48	22	32	50
BRAF mut status		*n=339*	*n=47*	*n=116*	*n=30*	*n=65*	*n=81*
	Wild-type	303	27	116	27	59	74
	Mutated	36	20	0	3	6	7
TP53 mut status		*n=339*	*n=47*	*n=116*	*n=30*	*n=65*	*n=81*
	Wild-type	101	18	21	17	20	25
	Mutated	238	29	95	13	45	56
APC mut status		*n=339*	*n=47*	*n=116*	*n=30*	*n=65*	*n=81*
	Wild-type	50	18	7	3	14	8
	Mutated	289	29	109	27	51	73

TCGA		All samples	CMS1	CMS2	CMS3	CMS4	Unclassified
**Clinico-pathological features**		**Statistics**	**Statistics**	**Statistics**	**Statistics**	**Statistics**	**Statistics**
Age		*n=461*	*n=73*	*n=188*	*n=58*	*n=109*	*n=33*
	Median (range)	67 (31, 90)	77 (34, 90)	66 (34, 90)	67 (31, 88)	65 (37, 90)	66 (43, 89)
Gender		*n=461*	*n=73*	*n=188*	*n=58*	*n=109*	*n=33*
	Male	245	32	105	30	59	19
	Female	216	41	83	28	50	14
Site		*n=447*	*n=70*	*n=182*	*n=57*	*n=106*	*n=32*
	Right colon	194	63	*48*	30	37	16
	Left colon	253	7	134	27	69	16
Stage at diagnosis		*n=444*	*n=72*	*n=181*	*n=55*	*n=104*	*n=32*
	I	99	18	43	20	11	7
	II	154	35	56	21	34	8
	III	128	15	50	11	38	14
	IV	63	4	32	3	21	3
Overall survival		*n=460*	*n=73*	*n=187*	*n=58*	*n=109*	*n=33*
	Median (range)	22 (0, 150)	21 (0, 133)	23 (0, 141)	25 (0, 142)	22 (0, 150)	17 (0, 65)
Progression-free interval		*n=460*	*n=73*	*n=187*	*n=58*	*n=109*	*n=33*
	Median (range)	19 (0, 150)	18 (0, 133)	21 (0, 141)	20 (0, 142)	17 (0, 150)	15 (0, 42)
**Major molecular features**							
Microsatellite status		*n=434*	*n=67*	*n=174*	*n=55*	*n=106*	*n=32*
	Stable (MSS)	363	10	173	46	102	32
	Instable (MSI)	71	57	1	9	4	0
CIMP		*n=357*	*n=67*	*n=142*	*n=46*	*n=82*	*n=20*
	CIMP.High	59	45	3	9	0	2
	CIMP.Low	70	11	22	19	14	4
	CIMP.Negative	228	11	117	18	68	14
**Selected mutations**							
KRAS mut status		*n=392*	*n=60*	*n=152*	*n=49*	*n=102*	*n=29*
	Wild-type	228	44	101	11	61	11
	Mutated	164	16	51	38	41	18
BRAF mut status		*n=392*	*n=60*	*n=152*	*n=49*	*n=102*	*n=29*
	Wild-type	351	26	152	47	97	29
	Mutated	41	34	0	2	5	0
TP53 mut status		*n=392*	*n=60*	*n=152*	*n=49*	*n=102*	*n=29*
	Wild-type	166	39	48	32	34	13
	Mutated	226	21	104	17	68	16
APC mut status		*n=392*	*n=60*	*n=152*	*n=49*	*n=102*	*n=29*
	Wild-type	115	44	21	15	30	5
	Mutated	277	16	131	34	72	24

GRAMPIAN		**All samples**	**CMS1**	**CMS2**	**CMS3**	**CMS4**	**Unclassified**
**Clinico-pathological features**		**Statistics**	**Statistics**	**Statistics**	**Statistics**	**Statistics**	**Statistics**
Age		*n=223*	*n=20*	*n=63*	*n=35*	*n=26*	*n=79*
	Median (range)	67 (31, 91)	72 (45, 86)	67 (31, 89)	69 (36, 83)	60 (40, 79)	67 (43, 91)
Gender		*n=223*	*n=20*	*n=63*	*n=35*	*n=26*	*n=79*
	Male	142	9	43	21	16	53
	Female	81	11	20	14	10	26
Site		*223*	*n=20*	*n=63*	*n=35*	*n=26*	*n=79*
	Right colon	0	0	0	0	0	0
	Left colon	223	20	63	35	26	79
Stage at diagnosis		*n=190*	*n=15*	*n=56*	*n=25*	*n=25*	*n=69*
	I	70	2	27	9	8	24
	II	61	5	14	9	9	24
	III	53	8	14	7	6	18
	IV	6	0	1	0	2	3
Relapse-free survival		*n=125*	*n=10*	*n=35*	*n=24*	*n=14*	*n=42*
	Median (range)	36 (4, 113)	36 (24, 36)	36 (4, 41)	36 (6, 54)	36 (9, 36)	36 (6, 113)
**Major molecular features**							
Microsatellite status		*n=181*	*n=19*	*n=51*	*n=30*	*n=21*	*n=60*
	Stable (MSS)	178	16	31	30	21	60
	Instable (MSI)	3	3	0	0	0	0
CIMP		*n=118*	*n=11*	*n=40*	*n=24*	*n=11*	*n=32*
	CIMP.High	21	5	2	8	0	6
	CIMP.Low	39	4	18	9	1	7
	CIMP.Negative	58	2	20	7	10	19
**Selected mutations**							
KRAS mut status		*n=181*	*n=19*	*n=51*	*n=30*	*n=21*	*n=60*
	Wild-type	84	4	32	10	12	26
	Mutated	97	15	19	20	9	34
BRAF mut status		*n=181*	*n=19*	*n=51*	*n=30*	*n=21*	*n=60*
	Wild-type	178	16	51	30	21	60
	Mutated	3	3	0	0	0	0
TP53 mut status		*n=181*	*n=19*	*n=51*	*n=30*	*n=21*	*n=60*
	Wild-type	32	5	7	7	5	8
	Mutated	149	14	44	23	16	52
APC mut status		*n=181*	*n=19*	*n=51*	*n=30*	*n=21*	*n=60*
	Wild-type	15	7	1	3	1	3
	Mutated	166	12	50	27	20	57
