# Supplementary figures and images for: Image-based consensus molecular subtype (imCMS) classification of colorectal cancer using deep learning
Source: Gut. 2020 Jul 20;70(3):544–54. doi: 10.1136/gutjnl-2019-319866 (PMC7873419; doi:10.1136/gutjnl-2019-319866)

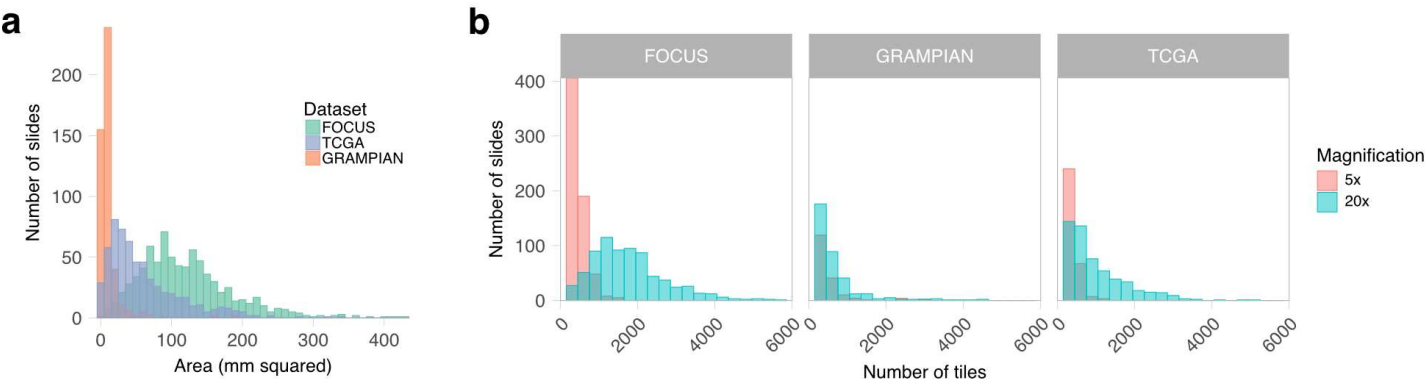

Supplement: Supplementary data [file gutjnl-2019-319866supp002.pdf]

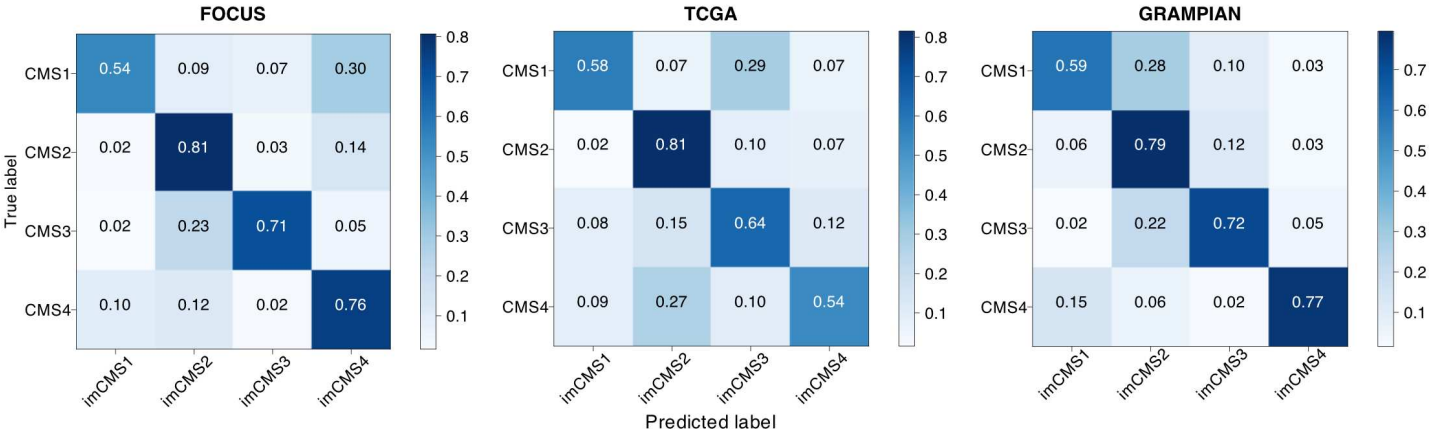

Supplement: Supplementary data [file gutjnl-2019-319866supp003.pdf]

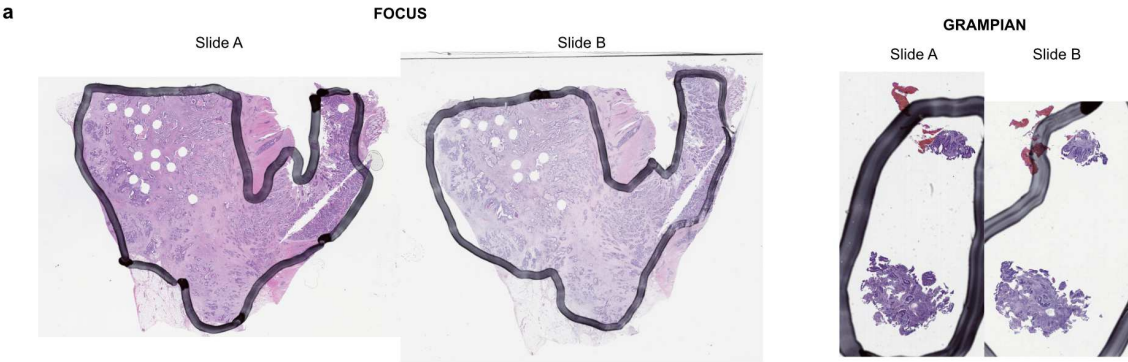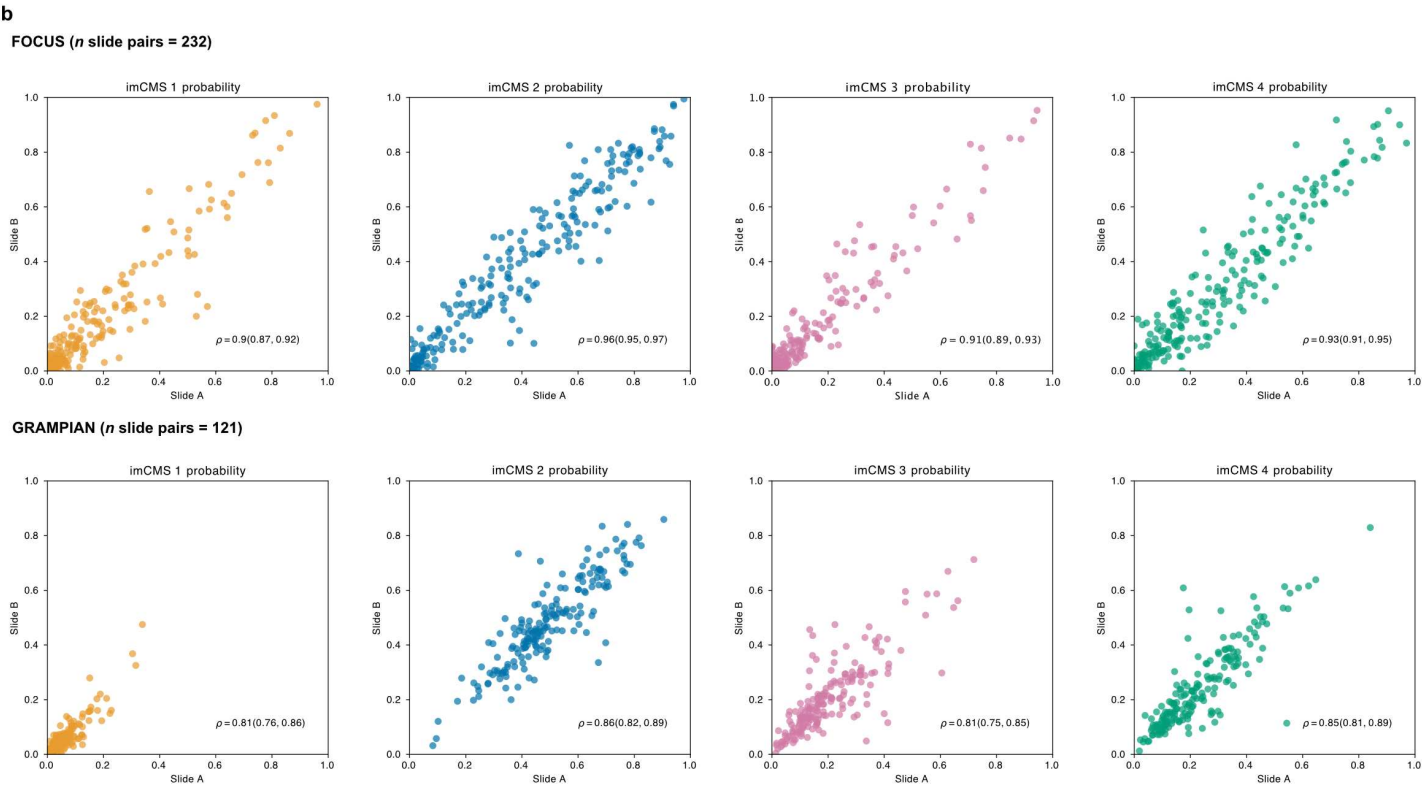

Supplement: Supplementary data [file gutjnl-2019-319866supp004.pdf]

a

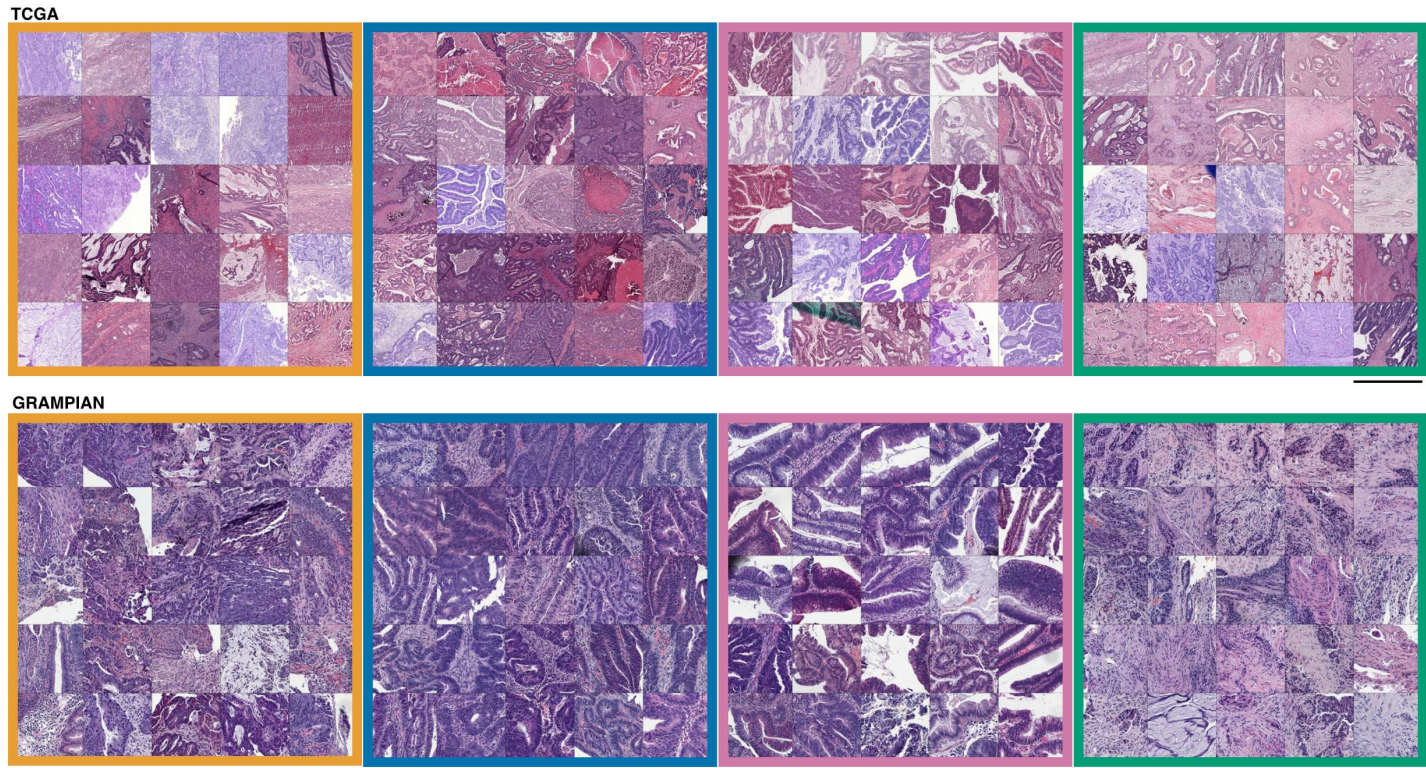

b

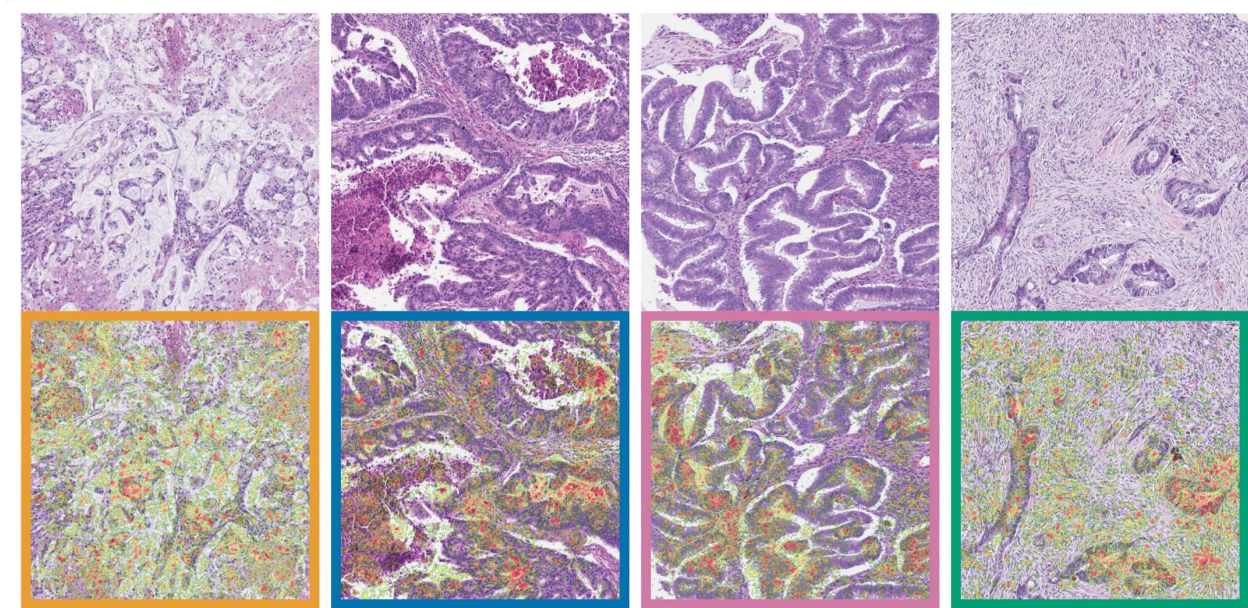

Supplement: Supplementary data [file gutjnl-2019-319866supp005.pdf]

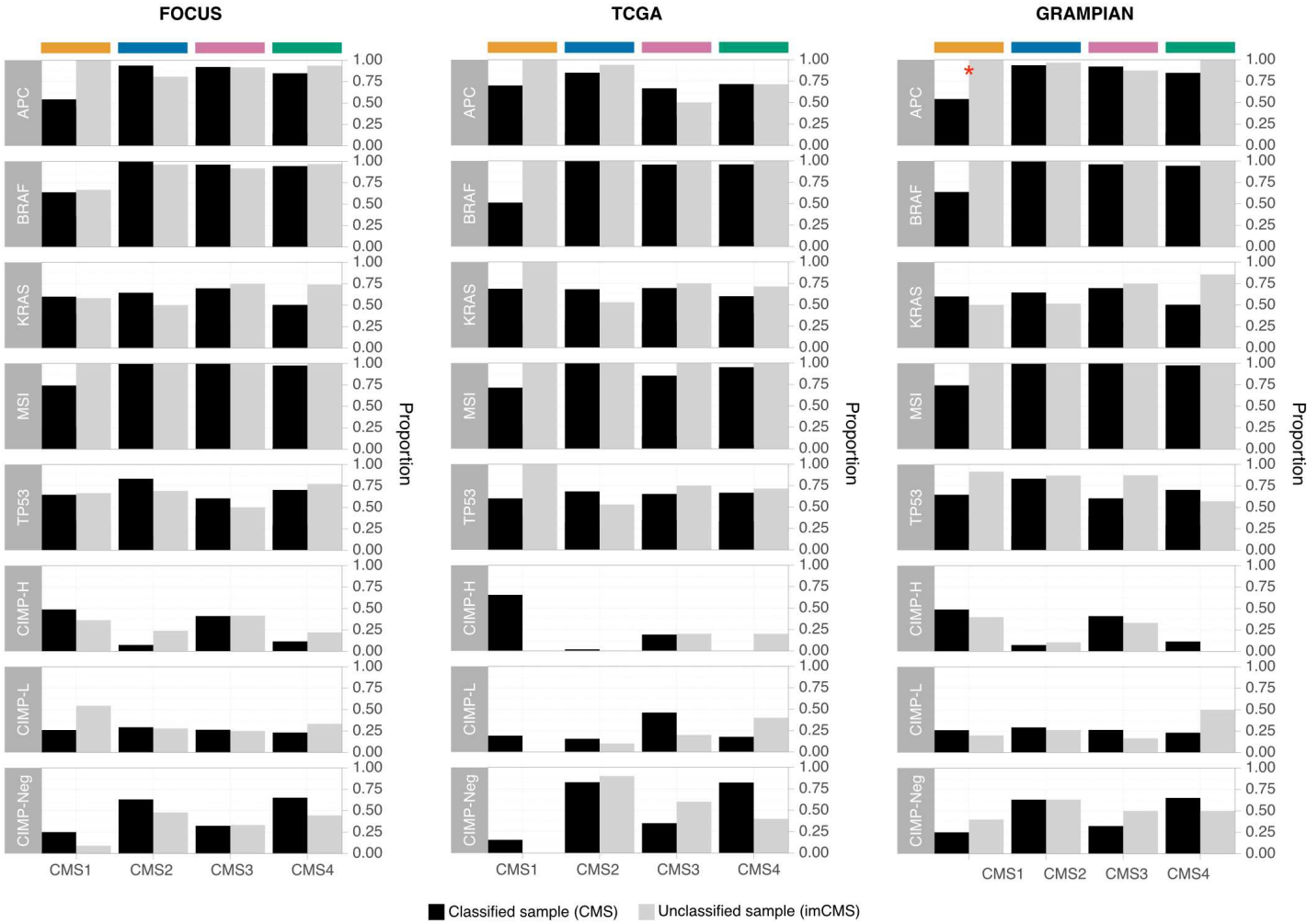

Supplement: Supplementary data [file gutjnl-2019-319866supp006.pdf]

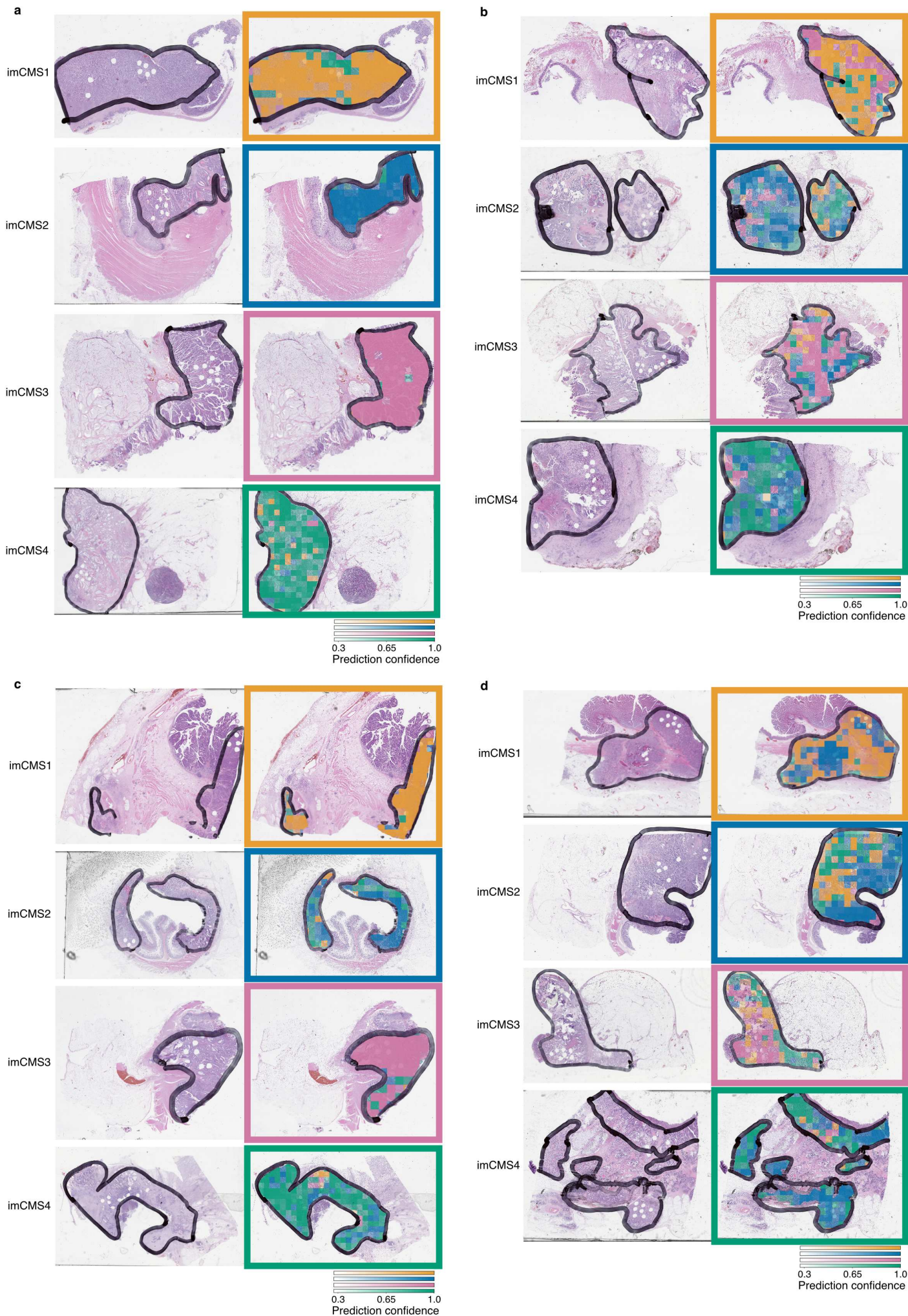

Supplement: Supplementary data [file gutjnl-2019-319866supp007.pdf]

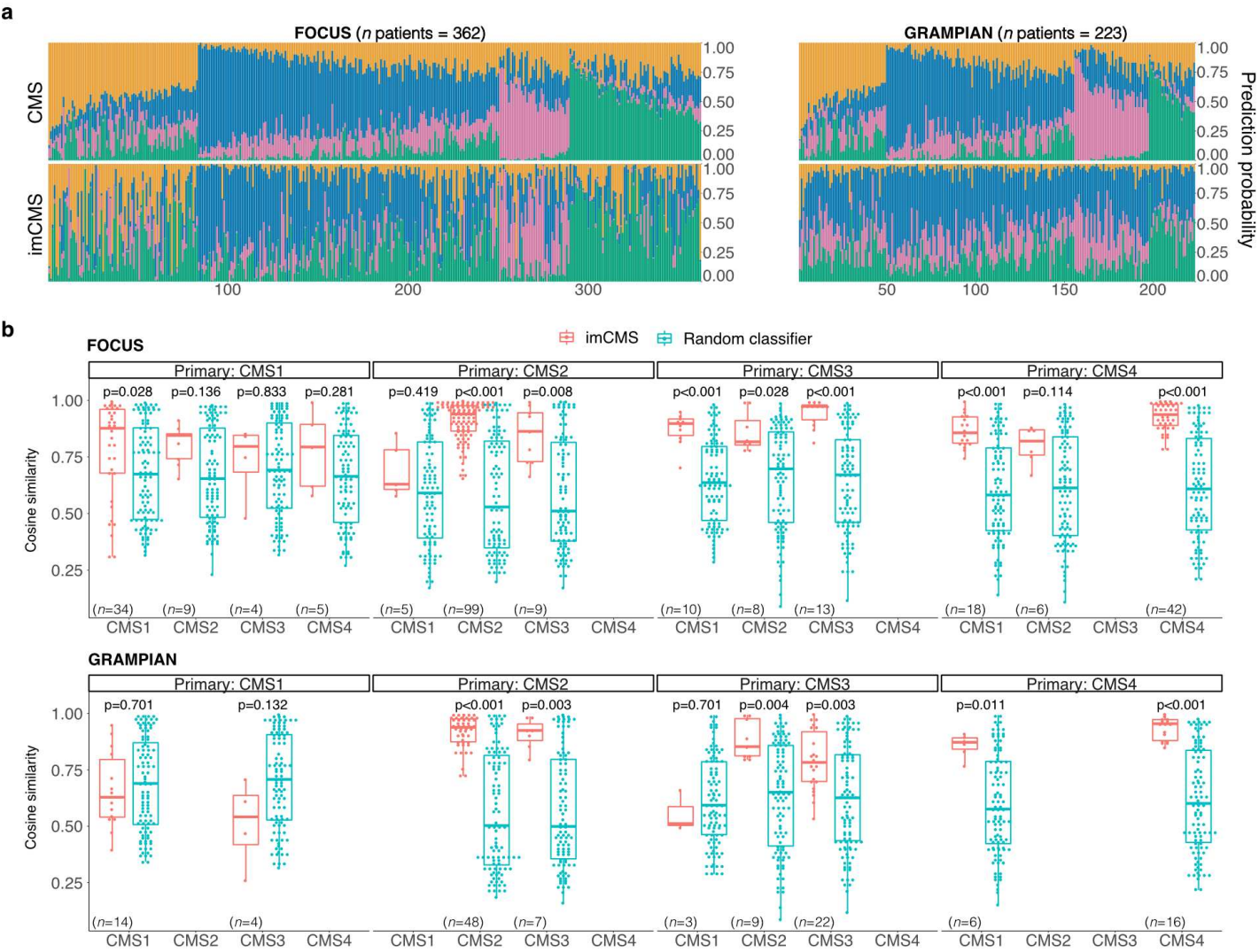

Supplement: Supplementary data [file gutjnl-2019-319866supp008.pdf]

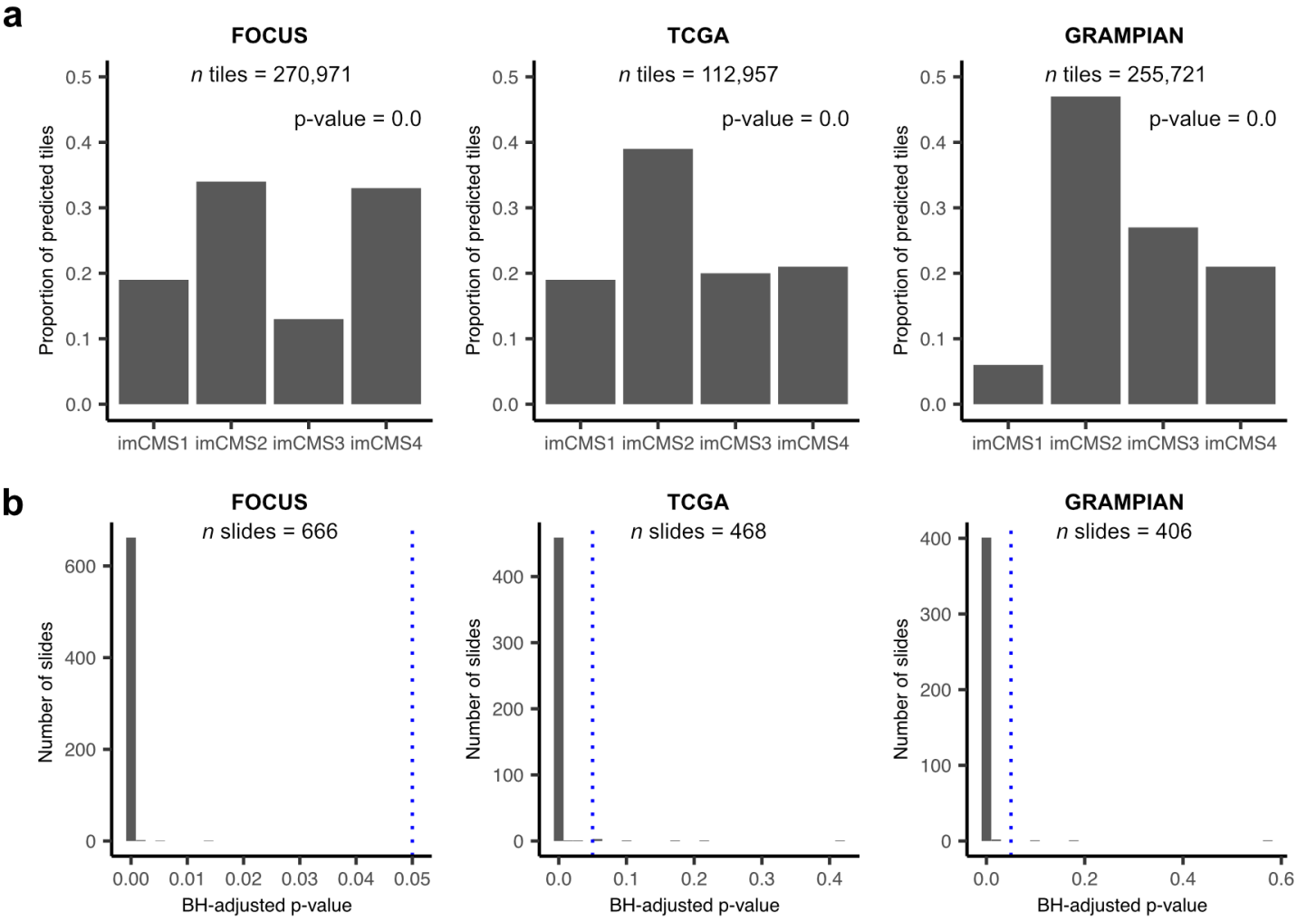

Supplement: Supplementary data [file gutjnl-2019-319866supp010.pdf]

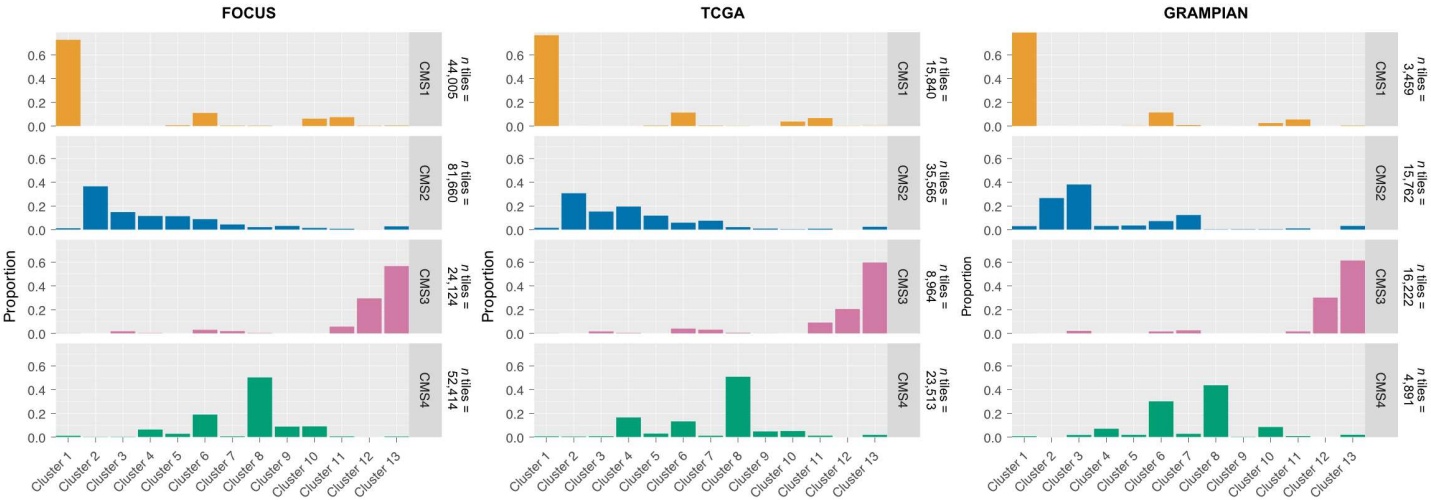

Supplement: Supplementary data [file gutjnl-2019-319866supp011.pdf]

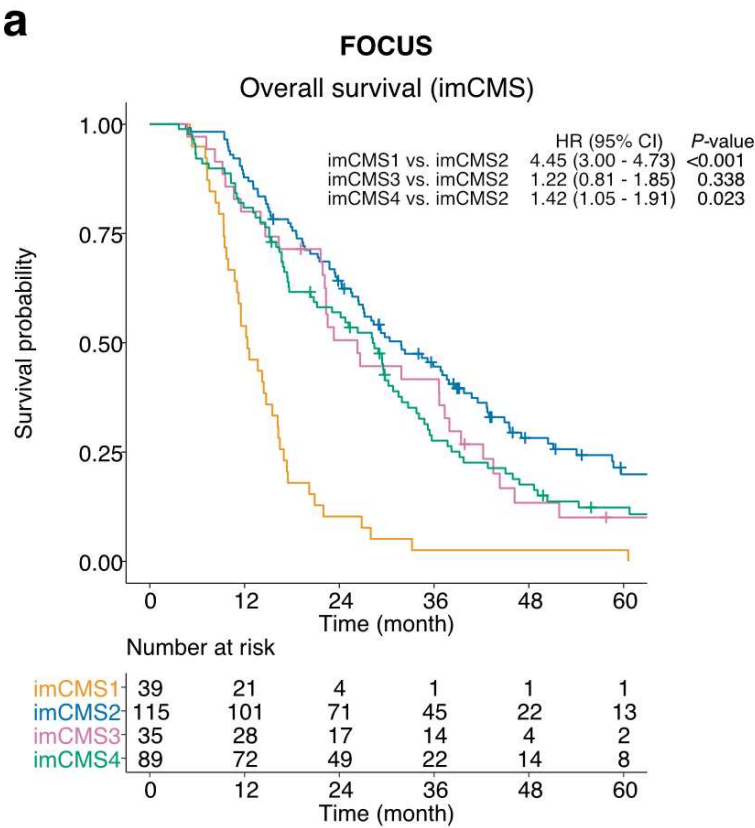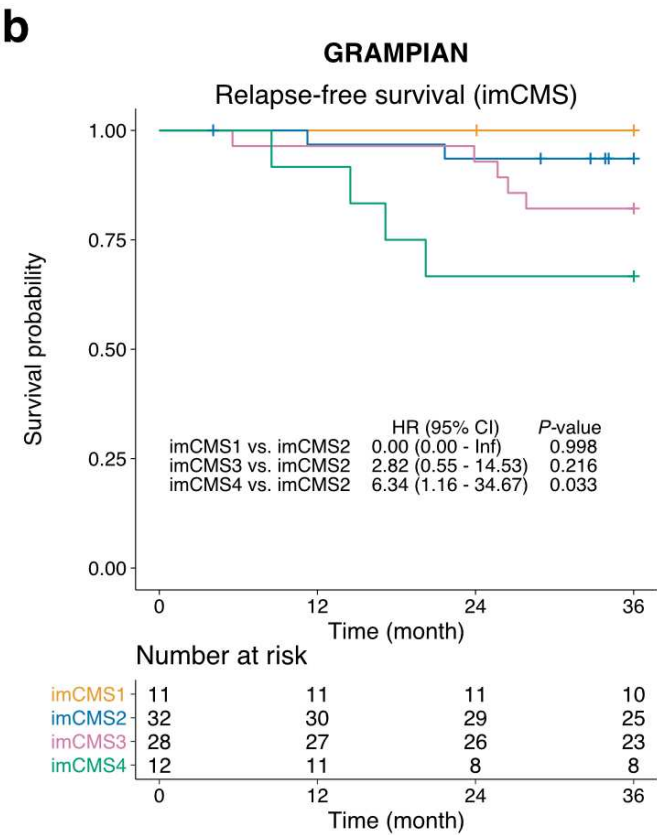

Supplement: Supplementary data [file gutjnl-2019-319866supp009.pdf]
