# Supplementary material for: Image-based consensus molecular subtype (imCMS) classification of colorectal cancer using deep learning
Source: Gut. 2020 Jul 20;70(3):544–54. doi: 10.1136/gutjnl-2019-319866 (PMC7873419; doi:10.1136/gutjnl-2019-319866)
Supplement: Supplementary data [file gutjnl-2019-319866supp013.pdf]

Table S02

FOCUS

n slides = 510, n patients = 278

CMS	3X					
	**Model 1 (Fold 1)**	**Model 2 (Fold 2)**	**Model 3 (Fold 3)**	**Model 4 (Fold 4)**	**Model 5 (Fold 5)**	**Overall**
**CMS1**	0.8 (0.65,0.94)	0.86 (0.76,0.98)	0.94 (0.91,1)	0.82 (0.73,0.95)	0.74 (0.63,0.85)	0.83 (0.78,0.87)
**CMS2**	0.87 (0.8,0.95)	0.93 (0.89,0.99)	0.95 (0.92,0.99)	0.82 (0.73,0.91)	0.84 (0.77,0.93)	0.88 (0.85,0.92)
**CMS3**	0.94 (0.89,1.02)	0.98 (0.96,1.01)	0.92 (0.87,0.99)	0.91 (0.85,0.99)	0.92 (0.86,1.01)	0.93 (0.91,0.97)
**CMS4**	0.9 (0.85,0.98)	0.82 (0.75,0.91)	0.84 (0.73,0.95)	0.89 (0.83,0.98)	0.9 (0.84,0.98)	0.87 (0.83,0.9)
**Macro-average**	0.88 (0.82,0.93)	0.9 (0.86,0.95)	0.91 (0.87,0.97)	0.86 (0.8,0.93)	0.85 (0.81,0.91)	0.88 (0.86,0.9)

CMS	12X					
	**Model 1**	**Model 2**	**Model 3**	**Model 4**	**Model 5**	**Overall**
**CMS1**	0.89 (0.81,0.99)	0.85 (0.76,0.95)	0.9 (0.84,1.02)	0.89 (0.83,0.97)	0.74 (0.6,0.89)	0.85 (0.81,0.89)
**CMS2**	0.89 (0.83,0.96)	0.92 (0.87,0.97)	0.89 (0.83,0.95)	0.84 (0.78,0.93)	0.84 (0.76,0.92)	0.86 (0.83,0.91)
**CMS3**	0.93 (0.88,0.99)	0.92 (0.86,0.99)	0.82 (0.66,1.04)	0.89 (0.82,0.97)	0.95 (0.92,1.01)	0.9 (0.85,0.94)
**CMS4**	0.9 (0.85,0.96)	0.86 (0.8,0.95)	0.84 (0.76,0.96)	0.89 (0.82,0.97)	0.81 (0.74,0.92)	0.85 (0.82,0.89)
**Macro-average**	0.9 (0.86,0.95)	0.89 (0.85,0.93)	0.86 (0.8,0.93)	0.88 (0.83,0.93)	0.84 (0.78,0.89)	0.87 (0.84,0.89)

TCGA

n slides = 431, n patients = 430

CMS	3x					
	**Model 1**	**Model 2**	**Model 3**	**Model 4**	**Model 5**	**Ensemble model**
**CMS1**	0.85 (0.81,0.93)	0.79 (0.73,0.85)	0.77 (0.7,0.85)	0.82 (0.76,0.87)	0.77 (0.7,0.85)	0.82 (0.77,0.89)
**CMS2**	0.83 (0.8,0.87)	0.75 (0.7,0.81)	0.79 (0.75,0.84)	0.78 (0.74,0.82)	0.78 (0.73,0.83)	0.84 (0.81,0.88)
**CMS3**	0.76 (0.7,0.84)	0.72 (0.63,0.79)	0.69 (0.58,0.78)	0.75 (0.67,0.84)	0.72 (0.64,0.81)	0.75 (0.67,0.83)
**CMS4**	0.79 (0.74,0.85)	0.78 (0.72,0.84)	0.79 (0.74,0.84)	0.79 (0.75,0.86)	0.71 (0.66,0.78)	0.84 (0.79,0.88)
**Macro-average**	0.81 (0.78,0.84)	0.76 (0.73,0.8)	0.76 (0.72,0.79)	0.79 (0.75,0.82)	0.74 (0.71,0.79)	0.81 (0.78,0.84)

CMS	12x					
	**Model 1**	**Model 2**	**Model 3**	**Model 4**	**Model 5**	**Ensemble model**
**CMS1**	0.8 (0.74,0.87)	0.78 (0.73,0.84)	0.77 (0.72,0.86)	0.83 (0.77,0.89)	0.76 (0.69,0.83)	0.82 (0.76,0.88)
**CMS2**	0.76 (0.72,0.82)	0.79 (0.74,0.85)	0.79 (0.75,0.84)	0.79 (0.74,0.84)	0.81 (0.76,0.86)	0.82 (0.78,0.86)
**CMS3**	0.76 (0.68,0.82)	0.66 (0.59,0.74)	0.71 (0.61,0.79)	0.69 (0.6,0.77)	0.75 (0.69,0.84)	0.74 (0.68,0.81)
**CMS4**	0.76 (0.71,0.81)	0.76 (0.71,0.84)	0.74 (0.69,0.81)	0.77 (0.72,0.84)	0.72 (0.66,0.79)	0.82 (0.77,0.87)
**Macro-average**	0.77 (0.73,0.8)	0.75 (0.71,0.78)	0.75 (0.72,0.78)	0.77 (0.74,0.8)	0.76 (0.73,0.8)	0.8 (0.77,0.83)

GRAMPIAN

n slides= 265, n patients = 144

CMS	3x					
	**Model 1**	**Model 2**	**Model 3**	**Model 4**	**Model 5**	**Ensemble model**
**CMS1**	0.69 (0.56,0.8)	0.69 (0.57,0.8)	0.68 (0.59,0.79)	0.72 (0.61,0.81)	0.58 (0.41,0.67)	0.76 (0.65,0.85)
**CMS2**	0.72 (0.65,0.81)	0.65 (0.6,0.74)	0.76 (0.69,0.83)	0.65 (0.58,0.72)	0.77 (0.72,0.83)	0.78 (0.72,0.83)
**CMS3**	0.74 (0.69,0.8)	0.72 (0.67,0.81)	0.76 (0.67,0.83)	0.76 (0.68,0.83)	0.8 (0.74,0.86)	0.81 (0.75,0.9)
**CMS4**	0.91 (0.86,0.95)	0.91 (0.87,0.96)	0.93 (0.9,0.97)	0.76 (0.69,0.84)	0.91 (0.86,0.98)	0.94 (0.9,0.98)
**Macro-average**	0.77 (0.72,0.81)	0.75 (0.71,0.79)	0.78 (0.73,0.82)	0.72 (0.68,0.77)	0.76 (0.71,0.8)	0.82 (0.78,0.87)

CMS	12x					
	**Model 1**	**Model 2**	**Model 3**	**Model 4**	**Model 5**	**Ensemble model**
**CMS1**	0.75 (0.67,0.86)	0.75 (0.67,0.84)	0.72 (0.59,0.83)	0.84 (0.76,0.91)	0.64 (0.5,0.73)	0.82 (0.75,0.92)
**CMS2**	0.75 (0.68,0.81)	0.63 (0.58,0.71)	0.7 (0.64,0.77)	0.69 (0.63,0.76)	0.75 (0.69,0.81)	0.75 (0.71,0.82)
**CMS3**	0.79 (0.73,0.84)	0.54 (0.46,0.62)	0.79 (0.73,0.86)	0.82 (0.76,0.88)	0.75 (0.67,0.83)	0.79 (0.73,0.88)
**CMS4**	0.93 (0.88,0.98)	0.9 (0.84,0.97)	0.91 (0.87,0.96)	0.86 (0.8,0.94)	0.94 (0.9,0.98)	0.94 (0.91,0.99)
**Macro-average**	0.8 (0.77,0.86)	0.7 (0.67,0.74)	0.78 (0.73,0.82)	0.8 (0.76,0.84)	0.77 (0.72,0.81)	0.83 (0.79,0.86)
