# Supplementary material for: Image-based consensus molecular subtype (imCMS) classification of colorectal cancer using deep learning
Source: Gut. 2020 Jul 20;70(3):544–54. doi: 10.1136/gutjnl-2019-319866 (PMC7873419; doi:10.1136/gutjnl-2019-319866)
Supplement: Supplementary data [file gutjnl-2019-319866supp014.pdf]

Table S03

FOCUS 3X (majority vote)

n slides = 510, n patients = 278

Model1 / Fold 1	n slides	Count				Percentage				Macro average
		imCMS1	imCMS2	imCMS3	imCMS4	imCMS1	imCMS2	imCMS3	imCMS4	
**CMS1**	20	10	2	3	5	50	10	15	25	67
**CMS2**	40	0	31	1	8	0	78	3	20	
**CMS3**	13	0	5	8	0	0	38	62	0	
**CMS4**	24	2	3	0	19	8	13	0	79	

Model2 / Fold 2	n slides	Count				Percentage				Macro average
		imCMS1	imCMS2	imCMS3	imCMS4	imCMS1	imCMS2	imCMS3	imCMS4	
**CMS1**	19	11	1	3	4	58	5	16	21	66
**CMS2**	47	0	43	0	4	0	91	0	9	
**CMS3**	14	0	3	10	1	0	21	71	7	
**CMS4**	27	4	11	0	12	15	41	0	44	

Model3 / Fold 3	n slides	Count				Percentage				Macro average
		imCMS1	imCMS2	imCMS3	imCMS4	imCMS1	imCMS2	imCMS3	imCMS4	
**CMS1**	19	15	0	0	4	79	0	0	21	61
**CMS2**	46	1	43	0	2	2	93	0	4	
**CMS3**	11	3	6	2	0	27	55	18	0	
**CMS4**	25	2	10	0	13	8	40	0	52	

Model4 / Fold 4	n slides	Count				Percentage				Macro average
		imCMS1	imCMS2	imCMS3	imCMS4	imCMS1	imCMS2	imCMS3	imCMS4	
**CMS1**	20	6	9	0	5	30	45	0	25	57
**CMS2**	44	0	37	3	4	0	84	7	9	
**CMS3**	11	1	5	5	0	9	45	45	0	
**CMS4**	26	0	6	2	18	0	23	8	69	

Model5 / Fold 5	n slides	Count				Percentage				Macro average
		imCMS1	imCMS2	imCMS3	imCMS4	imCMS1	imCMS2	imCMS3	imCMS4	
**CMS1**	20	10	5	2	3	50	25	10	15	55
**CMS2**	45	8	28	3	6	18	62	7	13	
**CMS3**	13	3	3	7	0	23	23	54	0	
**CMS4**	26	6	6	0	14	23	23	0	54	

Overall	n slides	Count				Percentage				Macro average
		imCMS1	imCMS2	imCMS3	imCMS4	imCMS1	imCMS2	imCMS3	imCMS4	
**CMS1**	98	52	17	8	21	53	17	8	21	62
**CMS2**	222	9	182	7	24	4	82	3	11	
**CMS3**	62	7	22	32	1	11	35	52	2	
**CMS4**	128	14	36	2	76	11	28	2	59	

FOCUS 12X (majority vote)

n slides = 510, n patients = 278

Model1 / Fold 1	n slides	Count				Percentage				Macro average
		imCMS1	imCMS2	imCMS3	imCMS4	imCMS1	imCMS2	imCMS3	imCMS4	
**CMS1**	20	10	7	1	2	50	35	5	10	52
**CMS2**	40	0	38	0	2	0	95	0	5	
**CMS3**	13	1	8	4	0	8	62	31	0	
**CMS4**	24	2	14	0	8	8	58	0	33	

Model2 / Fold 2	n slides	Count				Percentage				Macro average
		imCMS1	imCMS2	imCMS3	imCMS4	imCMS1	imCMS2	imCMS3	imCMS4	
**CMS1**	19	11	0	3	5	58	0	16	26	68
**CMS2**	47	4	40	0	3	9	85	0	6	
**CMS3**	14	0	3	10	1	0	21	71	7	
**CMS4**	27	6	5	0	16	22	19	0	59	

Model3 / Fold 3	n slides	Count				Percentage				Macro average
		imCMS1	imCMS2	imCMS3	imCMS4	imCMS1	imCMS2	imCMS3	imCMS4	
**CMS1**	19	12	2	1	4	63	11	5	21	64
**CMS2**	46	2	37	1	6	4	80	2	13	
**CMS3**	11	4	3	4	0	36	27	36	0	
**CMS4**	25	2	3	1	19	8	12	4	76	

Model4 / Fold 4	n slides	Count				Percentage				Macro average
		imCMS1	imCMS2	imCMS3	imCMS4	imCMS1	imCMS2	imCMS3	imCMS4	
**CMS1**	20	18	2	0	0	90	10	0	0	68
**CMS2**	44	1	28	13	2	2	64	30	5	
**CMS3**	11	3	1	7	0	27	9	64	0	
**CMS4**	26	4	4	4	14	15	15	15	54	

Model5 / Fold 5	n slides	Count				Percentage				Macro average
		imCMS1	imCMS2	imCMS3	imCMS4	imCMS1	imCMS2	imCMS3	imCMS4	
**CMS1**	20	12	3	0	5	60	15	0	25	56
**CMS2**	45	5	25	0	15	11	56	0	33	
**CMS3**	13	3	4	5	1	23	31	38	8	
**CMS4**	26	6	2	0	18	23	8	0	69	

Overall	n slides	Count				Percentage				Macro average
		imCMS1	imCMS2	imCMS3	imCMS4	imCMS1	imCMS2	imCMS3	imCMS4	
**CMS1**	98	63	14	5	16	64	14	5	16	62
**CMS2**	222	12	168	14	28	5	76	6	13	
**CMS3**	62	11	19	30	2	18	31	48	3	
**CMS4**	128	20	28	5	75	16	22	4	59	

TCGA 3X (majority vote)

n slides = 431, n patients = 430

Model1 / Fold 1	n slides	Count				Percentage				Macro average
		imCMS1	imCMS2	imCMS3	imCMS4	imCMS1	imCMS2	imCMS3	imCMS4	
**CMS1**	73	36	8	23	6	49	11	32	8	60
**CMS2**	189	4	158	19	8	2	84	10	4	
**CMS3**	59	6	13	33	7	10	22	56	12	
**CMS4**	110	4	38	10	58	4	35	9	53	

Model 2	n slides	Count				Percentage				Macro average
		imCMS1	imCMS2	imCMS3	imCMS4	imCMS1	imCMS2	imCMS3	imCMS4	
**CMS1**	73	30	9	25	9	41	12	34	12	57
**CMS2**	189	10	95	70	14	5	50	37	7	
**CMS3**	59	4	7	41	7	7	12	69	12	
**CMS4**	110	4	20	12	74	4	18	11	67	

Model 3	n slides	Count				Percentage				Macro average
		imCMS1	imCMS2	imCMS3	imCMS4	imCMS1	imCMS2	imCMS3	imCMS4	
**CMS1**	73	37	6	24	6	51	8	33	8	59
**CMS2**	189	12	113	33	31	6	60	17	16	
**CMS3**	59	8	12	31	8	14	20	53	14	
**CMS4**	110	6	14	10	80	5	13	9	73	

Model 4	n slides	Count				Percentage				Macro average
		imCMS1	imCMS2	imCMS3	imCMS4	imCMS1	imCMS2	imCMS3	imCMS4	
**CMS1**	73	38	3	28	4	52	4	38	5	59
**CMS2**	189	11	92	77	9	6	49	41	5	
**CMS3**	59	4	6	43	6	7	10	73	10	
**CMS4**	110	4	19	18	69	4	17	16	63	

Model 5	n slides	Count				Percentage				Macro average
		imCMS1	imCMS2	imCMS3	imCMS4	imCMS1	imCMS2	imCMS3	imCMS4	
**CMS1**	73	45	11	16	1	62	15	22	1	54
**CMS2**	189	18	128	36	7	10	68	19	4	
**CMS3**	59	11	15	30	3	19	25	51	5	
**CMS4**	110	24	39	7	40	22	35	6	36	

Ensemble	n slides	Count				Percentage				Macro average
		imCMS1	imCMS2	imCMS3	imCMS4	imCMS1	imCMS2	imCMS3	imCMS4	
**CMS1**	73	40	5	22	6	55	7	30	8	59
**CMS2**	189	9	130	36	14	5	69	19	7	
**CMS3**	59	5	10	37	7	8	17	63	12	
**CMS4**	110	7	35	13	55	6	32	12	50	

TCGA 12X (majority vote)

n slides = 431, n patients = 430

Model 1	n slides	Count				Percentage				Macro average
		imCMS1	imCMS2	imCMS3	imCMS4	imCMS1	imCMS2	imCMS3	imCMS4	
**CMS1**	73	40	13	20	0	55	18	27	0	57
**CMS2**	189	10	145	30	4	5	77	16	2	
**CMS3**	59	10	16	32	1	17	27	54	2	
**CMS4**	110	11	46	8	45	10	42	7	41	

Model 2	n slides	Count				Percentage				Macro average
		imCMS1	imCMS2	imCMS3	imCMS4	imCMS1	imCMS2	imCMS3	imCMS4	
**CMS1**	73	54	9	10	0	74	12	14	0	58
**CMS2**	189	28	146	10	5	15	77	5	3	
**CMS3**	59	15	23	16	5	25	39	27	8	
**CMS4**	110	14	33	6	57	13	30	5	52	

Model 3	n slides	Count				Percentage				Macro average
		imCMS1	imCMS2	imCMS3	imCMS4	imCMS1	imCMS2	imCMS3	imCMS4	
**CMS1**	73	45	8	16	4	62	11	22	5	56
**CMS2**	189	28	120	26	15	15	63	14	8	
**CMS3**	59	14	16	26	3	24	27	44	5	
**CMS4**	110	11	23	16	60	10	21	15	55	

Model 4	n slides	Count				Percentage				Macro average
		imCMS1	imCMS2	imCMS3	imCMS4	imCMS1	imCMS2	imCMS3	imCMS4	
**CMS1**	73	53	1	18	1	73	1	25	1	57
**CMS2**	189	31	73	75	10	16	39	40	5	
**CMS3**	59	15	6	35	3	25	10	59	5	
**CMS4**	110	12	16	17	65	11	15	15	59	

Model 5	n slides	Count				Percentage				Macro average
		imCMS1	imCMS2	imCMS3	imCMS4	imCMS1	imCMS2	imCMS3	imCMS4	
**CMS1**	73	39	7	26	1	53	10	36	1	57
**CMS2**	189	16	139	29	5	8	74	15	3	
**CMS3**	59	6	13	35	5	10	22	59	8	
**CMS4**	110	12	36	14	48	11	33	13	44	

Ensemble	n slides	Count				Percentage				Macro average
		imCMS1	imCMS2	imCMS3	imCMS4	imCMS1	imCMS2	imCMS3	imCMS4	
**CMS1**	73	49	6	17	1	67	8	23	1	60
**CMS2**	189	18	135	28	8	10	71	15	4	
**CMS3**	59	9	11	36	3	15	19	61	5	
**CMS4**	110	14	38	12	46	13	35	11	42	

GRAMPIAN 3X (majority vote)

n slides = 265, n patients = 144

Model1 / Fold	n slides	Count				Percentage				Macro average
		imCMS1	imCMS2	imCMS3	imCMS4	imCMS1	imCMS2	imCMS3	imCMS4	
CMS1	39	5	26	5	3	13	67	13	8	47
CMS2	115	3	88	8	16	3	77	7	14	
CMS3	63	0	43	14	6	0	68	22	10	
CMS4	48	1	10	1	36	2	21	2	75	

Model 2	n slides	Count				Percentage				Macro average
		imCMS1	imCMS2	imCMS3	imCMS4	imCMS1	imCMS2	imCMS3	imCMS4	
CMS1	39	18	13	5	3	46	33	13	8	47
CMS2	115	23	76	10	6	20	66	9	5	
CMS3	63	11	39	13	0	17	62	21	0	
CMS4	48	1	21	0	26	2	44	0	54	

Model 3	n slides	Count				Percentage				Macro average
		imCMS1	imCMS2	imCMS3	imCMS4	imCMS1	imCMS2	imCMS3	imCMS4	
CMS1	39	5	26	7	1	13	67	18	3	42
CMS2	115	2	104	7	2	2	90	6	2	
CMS3	63	2	53	7	1	3	84	11	2	
CMS4	48	1	20	2	25	2	42	4	52	

Model 4	n slides	Count				Percentage				Macro average
		imCMS1	imCMS2	imCMS3	imCMS4	imCMS1	imCMS2	imCMS3	imCMS4	
CMS1	39	24	13	1	1	62	33	3	3	43
CMS2	115	29	69	6	11	25	60	5	10	
CMS3	63	20	23	15	5	32	37	24	8	
CMS4	48	12	22	1	13	25	46	2	27	

Model 5	n slides	Count				Percentage				Macro average
		imCMS1	imCMS2	imCMS3	imCMS4	imCMS1	imCMS2	imCMS3	imCMS4	
CMS1	39	2	35	2	0	5	90	5	0	38
CMS2	115	2	108	4	1	2	94	3	1	
CMS3	63	0	51	11	1	0	81	17	2	
CMS4	48	2	25	4	17	4	52	8	35	

Ensemble	n slides	Count				Percentage				Macro average
		imCMS1	imCMS2	imCMS3	imCMS4	imCMS1	imCMS2	imCMS3	imCMS4	
CMS1	39	12	22	4	1	31	56	10	3	49
CMS2	115	3	103	6	3	3	90	5	3	
CMS3	63	2	49	11	1	3	78	17	2	
CMS4	48	1	18	1	28	2	38	2	58	

GRAMPIAN 3X (random forest)

n slides = 265, n patients = 144

Model1 / Fold	n slides	Count				Percentage				Macro average
		imCMS1	imCMS2	imCMS3	imCMS4	imCMS1	imCMS2	imCMS3	imCMS4	
CMS1	39	11	10	16	2	28	26	41	5	58
CMS2	115	5	82	18	10	4	71	16	9	
CMS3	63	0	19	41	3	0	30	65	5	
CMS4	48	0	10	6	32	0	21	13	67	

Model 2	n slides	Count				Percentage				Macro average
		imCMS1	imCMS2	imCMS3	imCMS4	imCMS1	imCMS2	imCMS3	imCMS4	
CMS1	39	16	9	11	3	41	23	28	8	59
CMS2	115	15	73	25	2	13	63	22	2	
CMS3	63	2	24	37	0	3	38	59	0	
CMS4	48	1	11	2	34	2	23	4	71	

Model 3	n slides	Count				Percentage				Macro average
		imCMS1	imCMS2	imCMS3	imCMS4	imCMS1	imCMS2	imCMS3	imCMS4	
CMS1	39	14	8	14	3	36	21	36	8	61
CMS2	115	6	82	18	9	5	71	16	8	
CMS3	63	5	15	42	1	8	24	67	2	
CMS4	48	2	9	4	33	4	19	8	69	

Model 4	n slides	Count				Percentage				Macro average
		imCMS1	imCMS2	imCMS3	imCMS4	imCMS1	imCMS2	imCMS3	imCMS4	
CMS1	39	17	11	11	0	44	28	28	0	51
CMS2	115	22	59	15	19	19	51	13	17	
CMS3	63	5	17	34	7	8	27	54	11	
CMS4	48	2	15	5	26	4	31	10	54	

Model 5	n slides	Count				Percentage				Macro average
		imCMS1	imCMS2	imCMS3	imCMS4	imCMS1	imCMS2	imCMS3	imCMS4	
CMS1	39	16	15	8	0	41	38	21	0	64
CMS2	115	7	89	18	1	6	77	16	1	
CMS3	63	0	17	45	1	0	27	71	2	
CMS4	48	0	9	7	32	0	19	15	67	

Ensemble	n slides	Count				Percentage				Macro average
		imCMS1	imCMS2	imCMS3	imCMS4	imCMS1	imCMS2	imCMS3	imCMS4	
CMS1	39	19	10	10	0	49	26	26	0	67
CMS2	115	4	84	20	7	3	73	17	6	
CMS3	63	2	12	47	2	3	19	75	3	
CMS4	48	1	9	3	35	2	19	6	73	

GRAMPIAN 12X (majority vote)

n slides = 265, n patients = 144

Model 1	n slides	Count				Percentage				Macro average
		imCMS1	imCMS2	imCMS3	imCMS4	imCMS1	imCMS2	imCMS3	imCMS4	
CMS1	39	8	21	10	0	21	54	26	0	48
CMS2	115	1	99	14	1	1	86	12	1	
CMS3	63	0	36	27	0	0	57	43	0	
CMS4	48	2	25	1	20	4	52	2	42	

Model 2	n slides	Count				Percentage				Macro average
		imCMS1	imCMS2	imCMS3	imCMS4	imCMS1	imCMS2	imCMS3	imCMS4	
CMS1	39	15	1	23	0	38	3	59	0	38
CMS2	115	27	39	49	0	23	34	43	0	
CMS3	63	6	22	35	0	10	35	56	0	
CMS4	48	0	3	34	11	0	6	71	23	

Model 3	n slides	Count				Percentage				Macro average
		imCMS1	imCMS2	imCMS3	imCMS4	imCMS1	imCMS2	imCMS3	imCMS4	
CMS1	39	4	32	3	0	10	82	8	0	41
CMS2	115	3	101	8	3	3	88	7	3	
CMS3	63	0	51	12	0	0	81	19	0	
CMS4	48	2	23	1	22	4	48	2	46	

Model 4	n slides	Count				Percentage				Macro average
		imCMS1	imCMS2	imCMS3	imCMS4	imCMS1	imCMS2	imCMS3	imCMS4	
CMS1	39	29	5	5	0	74	13	13	0	48
CMS2	115	26	54	35	0	23	47	30	0	
CMS3	63	9	13	41	0	14	21	65	0	
CMS4	48	20	19	6	3	42	40	13	6	

Model 5	n slides	Count				Percentage				Macro average
		imCMS1	imCMS2	imCMS3	imCMS4	imCMS1	imCMS2	imCMS3	imCMS4	
CMS1	39	2	28	5	4	5	72	13	10	48
CMS2	115	0	104	7	4	0	90	6	3	
CMS3	63	0	54	9	0	0	86	14	0	
CMS4	48	0	9	0	39	0	19	0	81	

Ensemble	n slides	Count				Percentage				Macro average
		imCMS1	imCMS2	imCMS3	imCMS4	imCMS1	imCMS2	imCMS3	imCMS4	
CMS1	39	11	19	9	0	28	49	23	0	49
CMS2	115	5	96	13	1	4	83	11	1	
CMS3	63	0	38	25	0	0	60	40	0	
CMS4	48	2	21	3	22	4	44	6	46	

GRAMPIAN 12X (random forest)

n slides = 265, n patients = 144

Model 1	n slides	Count				Percentage				Macro average
		imCMS1	imCMS2	imCMS3	imCMS4	imCMS1	imCMS2	imCMS3	imCMS4	
CMS1	39	16	12	9	2	41	31	23	5	63
CMS2	115	3	87	17	8	3	76	15	7	
CMS3	63	0	20	41	2	0	32	65	3	
CMS4	48	1	12	1	34	2	25	2	71	

Model 2	n slides	Count				Percentage				Macro average
		imCMS1	imCMS2	imCMS3	imCMS4	imCMS1	imCMS2	imCMS3	imCMS4	
CMS1	39	18	10	8	3	46	26	21	8	59
CMS2	115	10	85	4	16	9	74	3	14	
CMS3	63	2	37	20	4	3	59	32	6	
CMS4	48	1	5	2	40	2	10	4	83	

Model 3	n slides	Count				Percentage				Macro average
		imCMS1	imCMS2	imCMS3	imCMS4	imCMS1	imCMS2	imCMS3	imCMS4	
CMS1	39	18	8	11	2	46	21	28	5	62
CMS2	115	14	70	19	12	12	61	17	10	
CMS3	63	1	17	43	2	2	27	68	3	
CMS4	48	0	11	3	34	0	23	6	71	

Model 4	n slides	Count				Percentage				Macro average
		imCMS1	imCMS2	imCMS3	imCMS4	imCMS1	imCMS2	imCMS3	imCMS4	
CMS1	39	8	13	10	8	21	33	26	21	44
CMS2	111	2	54	25	30	2	49	23	27	
CMS3	63	3	15	36	9	5	24	57	14	
CMS4	48	3	11	11	23	6	23	23	48	

Model 5	n slides	Count				Percentage				Macro average
		imCMS1	imCMS2	imCMS3	imCMS4	imCMS1	imCMS2	imCMS3	imCMS4	
CMS1	39	13	16	10	0	33	41	26	0	59
CMS2	115	7	80	26	2	6	70	23	2	
CMS3	63	5	17	41	0	8	27	65	0	
CMS4	48	5	8	2	33	10	17	4	69	

Ensemble	n slides	Count				Percentage				Macro average
		imCMS1	imCMS2	imCMS3	imCMS4	imCMS1	imCMS2	imCMS3	imCMS4	
CMS1	39	20	6	11	2	51	15	28	5	67
CMS2	115	9	78	16	12	8	68	14	10	
CMS3	63	0	20	43	0	0	32	68	0	
CMS4	48	1	5	3	39	2	10	6	81	
