# Supplementary material for: Image-based consensus molecular subtype (imCMS) classification of colorectal cancer using deep learning
Source: Gut. 2020 Jul 20;70(3):544–54. doi: 10.1136/gutjnl-2019-319866 (PMC7873419; doi:10.1136/gutjnl-2019-319866)
Supplement: Supplementary data [file gutjnl-2019-319866supp015.pdf]

Table S04

60% of CMS classified samples in FOCUS cohort was used to train the CMS classifier, 20% for validation, and 20% for test

		FOCUS									
		Model 1		Model 2		Model 3		Model 4		Model 5	
		Training									
CMS classifier	CMS	*n slides = 307*	*n patients = 166*	*n slides = 306*	*n patients = 167*	*n slides = 308*	*n patients = 166*	*n slides = 301*	*n patients = 165*	*n slides = 301*	*n patients = 165*
	CMS1	57	31	59	31	59	31	57	31	58	31
	CMS2	131	71	135	72	135	71	130	71	131	70
	CMS3	41	22	37	22	36	22	38	21	37	22
	CMS4	78	42	75	42	78	42	76	42	75	42
		Validation									
	CMS	*n slides = 96*	*n patients = 53*	*n slides = 98*	*n patients =53*	*n slides = 99*	*n patients = 55*	*n slides = 104*	*n patients = 57*	*n slides = 104*	*n patients = 57*
	CMS1	20	10	18	10	20	11	22	11	22	11
	CMS2	42	23	41	22	41	23	44	24	42	25
	CMS3	9	6	12	7	14	7	12	8	12	7
	CMS4	25	14	27	14	24	14	26	14	28	14
		Test									
	CMS	*n slides = 103*	*n patients = 57*	*n slides = 106*	*n patients = 58*	*n slides = 99*	*n patients = 55*	*n slides = 102*	*n patients =54*	*n slides = 100*	*n patients = 54*
	CMS1	21	11	21	11	19	10	19	10	18	10
	CMS2	45	24	46	26	42	24	46	24	43	22
	CMS3	12	8	13	7	12	7	11	6	14	8
	CMS4	25	14	26	14	26	14	26	14	25	14

100% of CMS classified samples in TCGA and GRAMPIAN were used to test the 5 models

		TCGA		GRAMPAIN	
CMS classifier	CMS	*n slides = 431*	*n patients = 430*	n slides = 265	n patients = 144
	CMS1	73	73	39	20
	CMS2	189	189	115	63
	CMS3	59	58	63	35
	CMS4	110	110	48	26

60% of CMS classified samples in FOCUS cohort was used to train the CMS classifier, 20% for validation, and 20% for test

		FOCUS									
		Model 1		Model 2		Model 3		Model 4		Model 5	
	Training										
CMS classifier	CMS	*n slides = 307*	*n patients = 166*	*n slides = 306*	*n patients = 167*	*n slides = 308*	*n patients = 166*	*n slides = 301*	*n patients = 165*	*n slides = 301*	*n patients = 165*
	CMS1	57	31	59	31	59	31	57	31	58	31
	CMS2	131	71	135	72	135	71	130	71	131	70
	CMS3	41	22	37	22	36	22	38	21	37	22
	CMS4	78	42	75	42	78	42	76	42	75	42
	Validation										
	CMS	*n slides = 96*	*n patients = 53*	*n slides = 98*	*n patients =53*	*n slides = 99*	*n patients = 55*	*n slides = 104*	*n patients = 57*	*n slides = 104*	*n patients = 57*
	CMS1	20	10	18	10	20	11	22	11	22	11
	CMS2	42	23	41	22	41	23	44	24	42	25
	CMS3	9	6	12	7	14	7	12	8	12	7
	CMS4	25	14	27	14	24	14	26	14	28	14
	Test										
	CMS	*n slides = 103*	*n patients = 57*	*n slides = 106*	*n patients = 58*	*n slides = 99*	*n patients = 55*	*n slides = 102*	*n patients =54*	*n slides = 100*	*n patients = 54*
	CMS1	21	11	21	11	19	10	19	10	18	10
	CMS2	45	24	46	26	42	24	46	24	43	22
	CMS3	12	8	13	7	12	7	11	6	14	8
	CMS4	25	14	26	14	26	14	26	14	25	14

All CMS classified samples in the FOCUS cohort, 30% of TCGA cohort, and 20% of the GRAMPIAN cohort were used to train the domain classifier in each model

		FOCUS		TCGA		GRAMPIAN	
Domain classifier	CMS	*n slides = 510*	*n patients = 278*	*n slides = 126*	*n patients = 126*	*n slides = 57*	*n patients = 34*
	CMS1	98	52	23	23	8	4
	CMS2	222	120	53	53	27	16
	CMS3	62	36	17	17	13	8
	CMS4	128	70	33	33	9	6

100% of CMS classified samples in TCGA and GRAMPIAN were used to test the 5 models

		TCGA		GRAMPAIN	
CMS classifier	CMS	*n slides = 431*	*n patients = 430*	n slides = 265	n patients = 144
	CMS1	73	73	39	20
	CMS2	189	189	115	63
	CMS3	59	58	63	35
	CMS4	110	110	48	26
