# Supplementary material for: Image-based consensus molecular subtype (imCMS) classification of colorectal cancer using deep learning
Source: Gut. 2020 Jul 20;70(3):544–54. doi: 10.1136/gutjnl-2019-319866 (PMC7873419; doi:10.1136/gutjnl-2019-319866)
Supplement: Supplementary data [file gutjnl-2019-319866supp016.pdf]

Table S05

Prediction	**FOCUS** $n\ tiles = 203{,}876$ $n\ slides = 510$	**TCGA** $n\ tiles = 104{,}614$ $n\ slides = 431$	**GRAMPIAN** $n\ tiles = 189{,}418$ $n\ slides = 265$
imCMS1	19%	19%	5%
imCMS2	36%	40%	46%
imCMS3	13%	20%	30%
imCMS4	32%	21%	19%

No unclassifed slides used
