# Supplementary material for: Image-based consensus molecular subtype (imCMS) classification of colorectal cancer using deep learning
Source: Gut. 2020 Jul 20;70(3):544–54. doi: 10.1136/gutjnl-2019-319866 (PMC7873419; doi:10.1136/gutjnl-2019-319866)
Supplement: Supplementary data [file gutjnl-2019-319866supp017.pdf]

Table S06

GRAMPIAN 12X

Trial 2: 20% of the the data was selected at random to train a random forest model to find an optimal decision cutoff

20% Training (n slides = 59, n patients = 34)									
**CMS**	**n slides**	**Count**				**Percentage**			
		**imCMS1**	**imCMS2**	**imCMS3**	**imCMS4**	**imCMS1**	**imCMS2**	**imCMS3**	**imCMS4**
**CMS1**	9	6	2	1	0	67	22	11	0
**CMS2**	29	1	23	4	1	3	79	14	3
**CMS3**	12	0	0	12	0	0	0	100	0
**CMS4**	9	0	0	0	9	0	0	0	100

80% Test (n slides = 206, n patients = 110)									
**CMS**	**n slides**	**Count**				**Percentage**			
		**imCMS1**	**imCMS2**	**imCMS3**	**imCMS4**	**imCMS1**	**imCMS2**	**imCMS3**	**imCMS4**
**CMS1**	30	22	5	3	0	73	17	10	0
**CMS2**	86	15	50	16	5	17	58	19	6
**CMS3**	51	2	7	39	3	4	14	76	6
**CMS4**	39	10	1	2	26	26	3	5	67

Overall (n slides = 265, n patients = 144)									
**CMS**	**n slides**	**Count**				**Percentage**			
		**imCMS1**	**imCMS2**	**imCMS3**	**imCMS4**	**imCMS1**	**imCMS2**	**imCMS3**	**imCMS4**
**CMS1**	39	28	7	4	0	72	18	10	0
**CMS2**	115	16	73	20	6	14	63	17	5
**CMS3**	63	2	7	51	3	3	11	81	5
**CMS4**	48	10	1	2	35	21	2	4	73

GRAMPIAN 12X

Trial 2: 20% of the the data was selected at random to train a random forest model to find an optimal decision cutoff

20% Training (n slides = 57, n patients = 29)									
**CMS**	**n slides**	**Count**				**Percentage**			
		**imCMS1**	**imCMS2**	**imCMS3**	**imCMS4**	**imCMS1**	**imCMS2**	**imCMS3**	**imCMS4**
**CMS1**	12	7	3	2	0	58	25	17	0
**CMS2**	24	1	21	2	0	4	88	8	0
**CMS3**	9	0	3	6	0	0	33	67	0
**CMS4**	8	0	0	0	8	0	0	0	100

80% Test (n slides = 208, n patients = 115)									
**CMS**	**n slides**	**Count**				**Percentage**			
		**imCMS1**	**imCMS2**	**imCMS3**	**imCMS4**	**imCMS1**	**imCMS2**	**imCMS3**	**imCMS4**
**CMS1**	27	19	8	0	0	70	30	0	0
**CMS2**	91	9	59	17	6	10	65	19	7
**CMS3**	54	3	5	43	3	6	9	80	6
**CMS4**	40	9	1	1	29	23	3	3	73

Overall (n slides = 265, n patients = 144)									
**CMS**	**n slides**	**Count**				**Percentage**			
		**imCMS1**	**imCMS2**	**imCMS3**	**imCMS4**	**imCMS1**	**imCMS2**	**imCMS3**	**imCMS4**
**CMS1**	39	26	11	2	0	67	28	5	0
**CMS2**	115	10	80	19	6	9	70	17	5
**CMS3**	63	3	8	49	3	5	13	78	5
**CMS4**	48	9	1	1	37	19	2	2	77

GRAMPIAN 12X

Trial 3: 20% of the the data was selected at random to train a random forest model to find an optimal decision cutoff

20% Training (n slides = 57, n patients = 29)									
**CMS**	**n slides**	**Count**				**Percentage**			
		**imCMS1**	**imCMS2**	**imCMS3**	**imCMS4**	**imCMS1**	**imCMS2**	**imCMS3**	**imCMS4**
**CMS1**	8	8	0	0	0	100	0	0	0
**CMS2**	18	2	11	2	3	11	61	11	17
**CMS3**	21	1	3	17	0	5	14	81	0
**CMS4**	8	0	0	0	8	0	0	0	100

80% Test (n slides = 208, n patients = 115)									
**CMS**	**n slides**	**Count**				**Percentage**			
		**imCMS1**	**imCMS2**	**imCMS3**	**imCMS4**	**imCMS1**	**imCMS2**	**imCMS3**	**imCMS4**
**CMS1**	31	22	6	2	1	71	19	6	3
**CMS2**	97	7	69	16	5	7	71	16	5
**CMS3**	42	2	6	31	3	5	14	74	7
**CMS4**	40	5	1	2	32	13	3	5	80

Overall (n slides = 265, n patients = 144)									
**CMS**	**n slides**	**Count**				**Percentage**			
		**imCMS1**	**imCMS2**	**imCMS3**	**imCMS4**	**imCMS1**	**imCMS2**	**imCMS3**	**imCMS4**
**CMS1**	39	30	6	2	1	77	15	5	3
**CMS2**	115	9	80	18	8	8	70	16	7
**CMS3**	63	3	9	48	3	5	14	76	5
**CMS4**	48	5	1	2	40	10	2	4	83
