# Supplementary material for: Image-based consensus molecular subtype (imCMS) classification of colorectal cancer using deep learning
Source: Gut. 2020 Jul 20;70(3):544–54. doi: 10.1136/gutjnl-2019-319866 (PMC7873419; doi:10.1136/gutjnl-2019-319866)
Supplement: Supplementary data [file gutjnl-2019-319866supp018.pdf]

Table S07

TCGA 3X

	**30% used for domain adversarial training** (n slides = 126, n patients = 126)		**70% unseen** (n slides = 305, n patients = 304)		**Combined** (n slides = 431, n patients = 430)	
	Model w/o DA training	Model w/ DA training	Model w/o DA training	Model w/ DA training	Model w/o DA training	Model w/ DA training
**imCMS1**	0.78 (0.66,0.92)	0.84 (0.75,0.96)	0.84 (0.78,0.93)	0.86 (0.81,0.94)	0.82 (0.77,0.89)	0.85 (0.8,0.9)
**imCMS2**	0.83 (0.76,0.91)	0.86 (0.8,0.94)	0.85 (0.81,0.9)	0.9 (0.87,0.95)	0.84 (0.81,0.88)	0.89 (0.85,0.92)
**imCMS3**	0.81 (0.69,0.95)	0.81 (0.7,0.97)	0.72 (0.62,0.84)	0.77 (0.68,0.88)	0.75 (0.67,0.83)	0.78 (0.71,0.85)
**imCMS4**	0.85 (0.75,0.92)	0.81 (0.7,0.91)	0.84 (0.79,0.89)	0.83 (0.77,0.89)	0.84 (0.79,0.88)	0.83 (0.78,0.87)
**macro avg**	0.82 (0.76,0.88)	0.83 (0.77,0.9)	0.81 (0.77,0.85)	0.84 (0.81,0.88)	0.81 (0.78,0.84)	0.84 (0.8,0.87)

GRAMPIAN 12x

	**20% used for domain adversarial training** (n slides = 57, n patients = 34)		**80% unseen** (n slides = 208, n patients = 110)		**Combined** (n slides = 265, n patients = 144)	
	Model w/o DA training	Model w/ DA training	Model w/o DA training	Model w/ DA training	Model w/o DA training	Model w/ DA training
**imCMS1**	0.81 (0.65,1.13)	0.91 (0.84,1.07)	0.82 (0.74,0.91)	0.86 (0.79,0.97)	0.82 (0.75,0.92)	0.87 (0.81,0.94)
**imCMS2**	0.68 (0.53,0.87)	0.72 (0.58,0.88)	0.78 (0.7,0.85)	0.81 (0.75,0.9)	0.75 (0.71,0.82)	0.79 (0.74,0.84)
**imCMS3**	0.75 (0.58,0.96)	0.68 (0.51,0.85)	0.8 (0.73,0.9)	0.83 (0.77,0.91)	0.79 (0.73,0.88)	0.8 (0.75,0.86)
**imCMS4**	0.9 (0.81,1.09)	0.89 (0.79,1.14)	0.95 (0.92,0.99)	0.94 (0.9,0.97)	0.94 (0.91,0.99)	0.93 (0.89,0.98)
**macro avg**	0.79 (0.7,0.9)	0.80 (0.71,0.89)	0.84 (0.79,0.88)	0.86 (0.82,0.91)	0.83 (0.79,0.86)	0.85 (0.82,0.89)
