# Supplementary material for: Image-based consensus molecular subtype (imCMS) classification of colorectal cancer using deep learning
Source: Gut. 2020 Jul 20;70(3):544–54. doi: 10.1136/gutjnl-2019-319866 (PMC7873419; doi:10.1136/gutjnl-2019-319866)
Supplement: Supplementary data [file gutjnl-2019-319866supp019.pdf]

Table S08

FOCUS 3X Adversarial Trianing (majority vote)

n slides = 510, n patients = 278

Model1 / Fold	n slides	Count				Percentage				Macro average
		imCMS1	imCMS2	imCMS3	imCMS4	imCMS1	imCMS2	imCMS3	imCMS4	
**CMS1**	20	12	1	0	7	60	5	0	35	73
**CMS2**	40	2	32	1	5	5	80	3	13	
**CMS3**	13	0	4	9	0	0	31	69	0	
**CMS4**	24	3	1	0	20	13	4	0	83	

Model2 / Fold	n slides	Count				Percentage				Macro average
		imCMS1	imCMS2	imCMS3	imCMS4	imCMS1	imCMS2	imCMS3	imCMS4	
**CMS1**	19	9	1	4	5	47	5	21	26	73
**CMS2**	47	0	41	0	6	0	87	0	13	
**CMS3**	14	0	2	12	0	0	14	86	0	
**CMS4**	27	6	2	0	19	22	7	0	70	

Model3 / Fold	n slides	Count				Percentage				Macro average
		imCMS1	imCMS2	imCMS3	imCMS4	imCMS1	imCMS2	imCMS3	imCMS4	
**CMS1**	19	13	0	0	6	68	0	0	32	70
**CMS2**	46	0	39	2	5	0	85	4	11	
**CMS3**	11	1	3	6	1	9	27	55	9	
**CMS4**	25	0	6	1	18	0	24	4	72	

Model4 / Fold	n slides	Count				Percentage				Macro average
		imCMS1	imCMS2	imCMS3	imCMS4	imCMS1	imCMS2	imCMS3	imCMS4	
**CMS1**	20	9	3	0	8	45	15	0	40	72
**CMS2**	44	2	33	2	7	5	75	5	16	
**CMS3**	11	0	1	10	0	0	9	91	0	
**CMS4**	26	2	2	2	20	8	8	8	77	

Model5 / Fold	n slides	Count				Percentage				Macro average
		imCMS1	imCMS2	imCMS3	imCMS4	imCMS1	imCMS2	imCMS3	imCMS4	
**CMS1**	20	10	4	3	3	50	20	15	15	64
**CMS2**	45	1	34	2	8	2	76	4	18	
**CMS3**	13	0	4	7	2	0	31	54	15	
**CMS4**	26	2	4	0	20	8	15	0	77	

Overall	n slides	Count				Percentage				Macro average
		imCMS1	imCMS2	imCMS3	imCMS4	imCMS1	imCMS2	imCMS3	imCMS4	
**CMS1**	98	53	9	7	29	54	9	7	30	70
**CMS2**	222	5	179	7	31	2	81	3	14	
**CMS3**	62	1	14	44	3	2	23	71	5	
**CMS4**	128	13	15	3	97	10	12	2	76	

TCGA 3X - Adversarial Training (majority vote)

n slides = 431, n patients = 430

Model 1	n slides	Count				Percentage				Macro average
		imCMS1	imCMS2	imCMS3	imCMS4	imCMS1	imCMS2	imCMS3	imCMS4	
CMS1	73	37	8	21	7	51	11	29	10	63
CMS2	189	7	152	17	13	4	80	9	7	
CMS3	59	4	10	37	8	7	17	63	14	
CMS4	110	11	25	10	64	10	23	9	58	

Model 2	n slides	Count				Percentage				Macro average
		imCMS1	imCMS2	imCMS3	imCMS4	imCMS1	imCMS2	imCMS3	imCMS4	
CMS1	73	43	8	16	6	59	11	22	8	61
CMS2	189	4	158	12	15	2	84	6	8	
CMS3	59	9	17	26	7	15	29	44	12	
CMS4	110	6	32	11	61	5	29	10	55	

Model 3	n slides	Count				Percentage				Macro average
		imCMS1	imCMS2	imCMS3	imCMS4	imCMS1	imCMS2	imCMS3	imCMS4	
CMS1	73	36	9	20	8	49	12	27	11	59
CMS2	189	8	149	12	20	4	79	6	11	
CMS3	59	4	15	32	8	7	25	54	14	
CMS4	110	12	25	13	60	11	23	12	55	

Model 4	n slides	Count				Percentage				Macro average
		imCMS1	imCMS2	imCMS3	imCMS4	imCMS1	imCMS2	imCMS3	imCMS4	
CMS1	73	43	1	24	5	59	1	33	7	60
CMS2	189	8	118	49	14	4	62	26	7	
CMS3	59	4	4	43	8	7	7	73	14	
CMS4	110	19	25	16	50	17	23	15	45	

Model 5	n slides	Count				Percentage				Macro average
		imCMS1	imCMS2	imCMS3	imCMS4	imCMS1	imCMS2	imCMS3	imCMS4	
CMS1	73	43	1	24	5	59	1	33	7	61
CMS2	189	8	118	49	14	4	62	26	7	
CMS3	59	4	4	43	8	7	7	73	14	
CMS4	110	12	29	13	56	11	26	12	51	

Overall	n slides	Count				Percentage				Macro average
		imCMS1	imCMS2	imCMS3	imCMS4	imCMS1	imCMS2	imCMS3	imCMS4	
CMS1	73	42	5	21	5	58	7	29	7	64
CMS2	189	3	154	18	14	2	81	10	7	
CMS3	59	5	9	38	7	8	15	64	12	
CMS4	110	10	30	11	59	9	27	10	54	

GRAMPIAN 12X - Adversarial Training (majority vote)

n slides = 265, n patients = 144

**Model 1**	**n slides**	**Count**				**Percentage**				**Macro average**
		**imCMS1**	**imCMS2**	**imCMS3**	**imCMS4**	**imCMS1**	**imCMS2**	**imCMS3**	**imCMS4**	
**CMS1**	39	5	17	10	7	13	44	26	18	58
**CMS2**	115	1	98	5	11	1	85	4	10	
**CMS3**	63	0	28	32	3	0	44	51	5	
**CMS4**	48	2	6	1	39	4	13	2	81	

**Model 2**	**n slides**	**Count**				**Percentage**				**Macro average**
		**imCMS1**	**imCMS2**	**imCMS3**	**imCMS4**	**imCMS1**	**imCMS2**	**imCMS3**	**imCMS4**	
**CMS1**	39	2	14	17	6	5	36	44	15	46
**CMS2**	115	0	90	18	7	0	78	16	6	
**CMS3**	63	0	31	32	0	0	49	51	0	
**CMS4**	48	0	21	3	24	0	44	6	50	

**Model 3**	**n slides**	**Count**				**Percentage**				**Macro average**
		**imCMS1**	**imCMS2**	**imCMS3**	**imCMS4**	**imCMS1**	**imCMS2**	**imCMS3**	**imCMS4**	
**CMS1**	39	1	29	2	7	3	74	5	18	46
**CMS2**	115	0	97	8	10	0	84	7	9	
**CMS3**	63	0	43	18	2	0	68	29	3	
**CMS4**	48	0	12	3	33	0	25	6	69	

**Model 4**	**n slides**	**Count**				**Percentage**				**Macro average**
		**imCMS1**	**imCMS2**	**imCMS3**	**imCMS4**	**imCMS1**	**imCMS2**	**imCMS3**	**imCMS4**	
**CMS1**	39	5	24	9	1	13	62	23	3	49
**CMS2**	115	2	98	13	2	2	85	11	2	
**CMS3**	63	1	36	23	3	2	57	37	5	
**CMS4**	48	0	18	0	30	0	38	0	63	

**Model 5**	**n slides**	**Count**				**Percentage**				**Macro average**
		**imCMS1**	**imCMS2**	**imCMS3**	**imCMS4**	**imCMS1**	**imCMS2**	**imCMS3**	**imCMS4**	
**CMS1**	39	3	34	1	1	8	87	3	3	43
**CMS2**	115	0	110	4	1	0	96	3	1	
**CMS3**	63	0	47	16	0	0	75	25	0	
**CMS4**	48	0	27	0	21	0	56	0	44	

**Overall**	**n slides**	**Count**				**Percentage**				**Macro average**
		**imCMS1**	**imCMS2**	**imCMS3**	**imCMS4**	**imCMS1**	**imCMS2**	**imCMS3**	**imCMS4**	
**CMS1**	39	3	24	8	4	8	62	21	10	49
**CMS2**	115	0	103	6	6	0	90	5	5	
**CMS3**	63	0	41	21	1	0	65	33	2	
**CMS4**	48	0	16	1	31	0	33	2	65	

GRAMPIAN 12X - Adversarial Training (random forest)

n slides = 265, n patients = 144

**Model 1**	**n slides**	**Count**				**Percentage**				**Macro average**
		**imCMS1**	**imCMS2**	**imCMS3**	**imCMS4**	**imCMS1**	**imCMS2**	**imCMS3**	**imCMS4**	
**CMS1**	39	14	10	13	2	36	26	33	5	62
**CMS2**	115	6	61	41	7	5	53	36	6	
**CMS3**	63	0	9	51	3	0	14	81	5	
**CMS4**	48	6	2	3	37	13	4	6	77	

**Model 2**	**n slides**	**Count**				**Percentage**				**Macro average**
		**imCMS1**	**imCMS2**	**imCMS3**	**imCMS4**	**imCMS1**	**imCMS2**	**imCMS3**	**imCMS4**	
**CMS1**	39	21	11	6	1	54	28	15	3	58
**CMS2**	115	7	79	19	10	6	69	17	9	
**CMS3**	63	3	28	28	4	5	44	44	6	
**CMS4**	48	10	5	2	31	21	10	4	65	

**Model 3**	**n slides**	**Count**				**Percentage**				**Macro average**
		**imCMS1**	**imCMS2**	**imCMS3**	**imCMS4**	**imCMS1**	**imCMS2**	**imCMS3**	**imCMS4**	
**CMS1**	39	13	21	5	0	33	54	13	0	62
**CMS2**	115	7	90	13	5	6	78	11	4	
**CMS3**	63	4	16	41	2	6	25	65	3	
**CMS4**	48	5	5	3	35	10	10	6	73	

**Model 4**	**n slides**	**Count**				**Percentage**				**Macro average**
		**imCMS1**	**imCMS2**	**imCMS3**	**imCMS4**	**imCMS1**	**imCMS2**	**imCMS3**	**imCMS4**	
**CMS1**	39	19	10	7	3	49	26	18	8	61
**CMS2**	115	10	84	14	7	9	73	12	6	
**CMS3**	63	6	13	43	1	10	21	68	2	
**CMS4**	48	7	12	3	26	15	25	6	54	

**Model 5**	**n slides**	**Count**				**Percentage**				**Macro average**
		**imCMS1**	**imCMS2**	**imCMS3**	**imCMS4**	**imCMS1**	**imCMS2**	**imCMS3**	**imCMS4**	
**CMS1**	39	13	15	10	1	33	38	26	3	67
**CMS2**	115	1	90	17	7	1	78	15	6	
**CMS3**	63	0	14	46	3	0	22	73	5	
**CMS4**	48	0	6	2	40	0	13	4	83	

**Overall**	**n slides**	**Count**				**Percentage**				**Macro average**
		**imCMS1**	**imCMS2**	**imCMS3**	**imCMS4**	**imCMS1**	**imCMS2**	**imCMS3**	**imCMS4**	
**CMS1**	39	23	11	4	1	59	28	10	3	72
**CMS2**	115	7	91	14	3	6	79	12	3	
**CMS3**	63	1	14	45	3	2	22	71	5	
**CMS4**	48	7	3	1	37	15	6	2	77	
