# Supplementary material for: Image-based consensus molecular subtype (imCMS) classification of colorectal cancer using deep learning
Source: Gut. 2020 Jul 20;70(3):544–54. doi: 10.1136/gutjnl-2019-319866 (PMC7873419; doi:10.1136/gutjnl-2019-319866)
Supplement: Supplementary data [file gutjnl-2019-319866supp020.pdf]

Table S09

Concordance of the imCMS predicion between pairs of slides from the same patient

no unclassified samples used

FOCUS 3X		Slide 2									Cohen's Kappa
		Count					Percentage				
		n pairs	imCMS1	imCMS2	imCMS3	imCMS4	imCMS1	imCMS2	imCMS3	imCMS4	
Slide 1	imCMS1	33	27	2	1	3	82	6	3	9	0.85 (0.79, 0.91)
	imCMS2	102	1	93	3	5	1	91	3	5	
	imCMS3	26	0	1	24	1	0	4	92	4	
	imCMS4	71	3	3	1	64	4	4	1	90	

GRAMPIAN 12X		Slide 2									Cohen's Kappa
		Count					Percentage				
		n pairs	imCMS1	imCMS2	imCMS3	imCMS4	imCMS1	imCMS2	imCMS3	imCMS4	
Slide 1	imCMS1	19	11	4	4	0	58	21	21	0	0.71 (0.61, 0.74)
	imCMS2	59	4	48	6	1	7	81	10	2	
	imCMS3	21	0	1	20	0	0	5	95	0	
	imCMS4	22	2	0	2	18	9	0	9	82	
