# Supplementary material for: Image-based consensus molecular subtype (imCMS) classification of colorectal cancer using deep learning
Source: Gut. 2020 Jul 20;70(3):544–54. doi: 10.1136/gutjnl-2019-319866 (PMC7873419; doi:10.1136/gutjnl-2019-319866)
Supplement: Supplementary data [file gutjnl-2019-319866supp021.pdf]

Table S10

FOCUS H&E slide A		Overall				CMS1			
		Classified (n=278)	Unclassified (n=84)			Classified (n=52)	Unclassified (n=13)		
**Major molecular features**		Statistics	Statistics	Odds ratio	P value	Statistics	Statistics	Odds ratio (95% CI)	P value
CIMP cluster		*n=238*	*n=75*		*0.353*	*n=41*	*n=11*		*1.000*
	CIMP.High	21%	28%			54%	45%		
	CIMP.Low	24%	33%			27%	45%		
	CIMP.Negative	54%	39%			20%	9%		
Microsatellite status		*n=258*	*n=81*	*0*	*0.353*	*n=47*	*n=13*	0	*0.353*
	Stable (MSS)	96%	100%	(0.000,1.249)		79%	100%	(0,1.504)	
	Instable (MSI)	4%	0%			21%	0%		
**Selected mutations**									
APC mut status		*n=258*	*n=81*	1.772	*0.480*	*n=47*	*n=13*	7.263	*0.353*
	Wild-type	16%	10%	(0.775,4.575)		38%	8%	(0.924,335.217)	
	Mutated	84%	90%			62%	92%		
BRAF mut status		*n=258*	*n=81*	0.748	*1.000*	*n=47*	*n=13*	0.846	*1.000*
	Wild-type	89%	91%	(0.265,1.839)		57%	62%	(0.188,3.47)	
	Mutated	11%	9%			43%	38%		
KRAS mut status		*n=258*	*n=81*	1.91	*0.229*	*n=47*	*n=13*	0.863	*1.000*
	Wild-type	54%	38%	(1.116,3.308)		66%	69%	(0.168,3.724)	
	Mutated	46%	62%			34%	31%		
TP53 mut status		*n=258*	*n=81*	0.936	*1.000*	*n=47*	*n=13*	1.389	*1.000*
	Wild-type	29%	31%	(0.529,1.685)		38%	31%	(0.325,7.105)	
	Mutated	71%	69%			62%	69%		

FOCUS H&E slide A		CMS2				CMS3				CMS4			
		Classified (n=120)	Unclassified (n=26)			Classified (n=36)	Unclassified (n=15)			Classified (n=70)	Unclassified (n=30)		
**Major molecular features**		Statistics	Statistics	Odds ratio (95% CI)	P value	Statistics	Statistics	Odds ratio (95% CI)	P value	Statistics	Statistics	Odds ratio (95% CI)	P value
CIMP cluster		*n=108*	*n=25*		*0.353*	*n=28*	*n=13*		*1.000*	*n=61*	*n=26*		*0.462*
	CIMP.High	6%	20%			46%	38%			15%	23%		
	CIMP.Low	27%	28%			18%	31%			21%	35%		
	CIMP.Negative	67%	52%			36%	31%			64%	42%		
Microsatellite status		*n=116*	*n=25*	0	*1.000*	*n=30*	*n=13*	0	*1.000*	*n=65*	*n=30*	0	*1.000*
	Stable (MSS)	100%	100%	(0,Inf)		100%	100%	(0,Inf)		98%	100%	(0,84.39)	
	Instable (MSI)	0%	0%			0%	0%			2%	0%	NA	
**Selected mutations**													
APC mut status		*n=116*	*n=25*	0.341	*0.353*	*n=30*	*n=13*	1.325	*1.000*	*n=65*	*n=30*	3.799	*0.353*
	Wild-type	6%	16%	(0.078,1.73)		10%	8%	(0.095,75.72)		22%	7%	(0.783,36.808)	
	Mutated	94%	84%			90%	92%			78%	93%		
BRAF mut status		*n=116*	*n=25*	0	*1.000*	*n=30*	*n=13*	0.755	*1.000*	*n=65*	*n=30*	0.342	*0.913*
	Wild-type	100%	100%	(0,Inf)		90%	92%	(0.013,10.566)		91%	97%	(0.007,3.028)	
	Mutated	0%	0%			10%	8%			9%	3%		
KRAS mut status		*n=116*	*n=25*	1.795	*0.479*	*n=30*	*n=13*	0.822	*1.000*	*n=65*	*n=30*	3.346	*0.229*
	Wild-type	59%	44%	(0.69,4.785)		27%	31%	(0.163,4.699)		51%	23%	(1.181,10.571)	
	Mutated	41%	56%			73%	69%			49%	77%		
TP53 mut status		*n=116*	*n=25*	0.473	*0.462*	*n=30*	*n=13*	1.511	*1.000*	*n=65*	*n=30*	1.455	*1.000*
	Wild-type	18%	32%	(0.165,1.439)		57%	46%	(0.34,6.945)		31%	23%	(0.497,4.687)	
	Mutated	82%	68%			43%	54%			69%	77%		

FOCUS H&E slide B		**Overall**				**CMS1**				**CMS2**				**CMS3**				**CMS4**			
		**Classified (n=265)**	**Unclassified (n=84)**			**Classified (n=48)**	**Unclassified (n=17)**			**Classified (n=118)**	**Unclassified (n=25)**			**Classified (n=31)**	**Unclassified (n=13)**			**Classified (n=68)**	**Unclassified (n=29)**		
**Major molecular features**		**Statistics**	**Statistics**	**Odds ratio (95% CI)**	**P value**	**Statistics**	**Statistics**	**Odds ratio (95% CI)**	**P value**	**Statistics**	**Statistics**	**Odds ratio (95% CI)**	**P value**	**Statistics**	**Statistics**	**Odds ratio (95% CI)**	**P value**	**Statistics**	**Statistics**	**Odds ratio (95% CI)**	**P value**
CIMP cluster		*n=238*	*n=75*		*0.305*	*n=41*	*n=11*		*0.662*	*n=108*	*n=25*		*0.221*	*n=28*	*n=12*		*1.000*	*n=61*	*n=27*		*0.521*
	CIMP.High	21%	28%			54%	36%			6%	24%			46%	42%			15%	22%		
	CIMP.Low	24%	33%			27%	55%			27%	28%			18%	25%			21%	33%		
	CIMP.Negative	54%	39%			20%	9%			67%	48%			36%	33%			64%	44%		
Microsatellite status		*n=258*	*n=81*	0	*0.305*	*n=47*	*n=12*	0	*0.349*	*n=116*	*n=26*	0	*1.000*	*n=30*	*n=12*	0	*1.000*	*n=65*	*n=31*	0	*1.000*
	Stable (MSS)	96%	100%	(0,1.249)		79%	100%	(0,1.645)		100%	100%	(0,Inf)		100%	100%	(0,Inf)		98%	100%	(0,81.671)	
	Instable (MSI)	4%	0%			21%	0%			0%	0%			0%	0%			2%	0%		
**Selected mutations**																					
APC mut status		*n=258*	*n=81*	1.772	*0.520*	*n=47*	*n=12*	Inf	*0.221*	*n=116*	*n=26*	0.273	*0.267*	*n=30*	*n=12*	1.217	*1.000*	*n=65*	*n=31*	3.934	*0.305*
	Wild-type	16%	10%	(0.775,4.575)		38%	0%	(1.471,Inf)		6%	19%	(0.067,1.199)		10%	8%	(0.086,69.894)		22%	6%	(0.813,38.108)	
	Mutated	84%	90%			62%	100%			94%	81%			90%	92%			78%	94%		
BRAF mut status		*n=258*	*n=81*	0.748	*1.000*	*n=47*	*n=12*	0.679	*1.000*	*n=116*	*n=26*	Inf	*0.499*	*n=30*	*n=12*	0.822	*1.000*	*n=65*	*n=31*	0.331	*0.845*
	Wild-type	89%	91%	(0.265,1.839)		57%	67%	(0.131,2.982)		100%	96%	(0.114,Inf)		90%	92%	(0.014,11.596)		91%	97%	(0.007,2.922)	
	Mutated	11%	9%			43%	33%			0%	4%			10%	8%			9%	3%		
KRAS mut status		*n=258*	*n=81*	1.91	*0.221*	*n=47*	*n=12*	1.376	*1.000*	*n=116*	*n=26*	1.413	*0.904*	*n=30*	*n=12*	1.089	*1.000*	*n=65*	*n=31*	2.932	*0.221*
	Wild-type	54%	38%	(1.116,3.308)		66%	58%	(0.295,6.011)		59%	50%	(0.55,3.638)		27%	25%	(0.196,7.821)		51%	26%	(1.071,8.746)	
	Mutated	46%	62%			34%	42%			41%	50%			73%	75%			49%	74%		
TP53 mut status		*n=258*	*n=81*	0.936	*1.000*	*n=47*	*n=12*	1.237	*1.000*	*n=116*	*n=26*	0.5	*0.499*	*n=30*	*n=12*	1.299	*1.000*	*n=65*	*n=31*	1.517	*0.887*
	Wild-type	29%	31%	(0.529,1.685)		38%	33%	(0.28,6.448)		18%	31%	(0.177,1.512)		57%	50%	(0.275,6.175)		31%	23%	(0.52,4.872)	
	Mutated	71%	69%			62%	67%			82%	69%			43%	50%			69%	77%		

TCGA H&E slide A		Overall				CMS1				CMS2				CMS3				CMS4			
		**Classified (n=430)**	**Unclassified (n=33)**			**Classified (n=73)**	**Unclassified (n=1)**			**Classified (n=189)**	**Unclassified (n=21)**			**Classified (n=58)**	**Unclassified (n=5)**			**Classified (n=110)**	**Unclassified (n=6)**		
**Major molecular features**		**Statistics**	**Statistics**	**Odds ratio (95% CI)**	**P value**	**Statistics**	**Statistics**	**Odds ratio (95% CI)**	**P value**	**Statistics**	**Statistics**	**Odds ratio (95% CI)**	**P value**	**Statistics**	**Statistics**	**Odds ratio (95% CI)**	**P value**	**Statistics**	**Statistics**	**Odds ratio (95% CI)**	**P value**
CIMP cluster		*n=337*	*n=20*		*1.000*	*n=67*	*n=0*			*n=142*	*n=10*		*1.000*	*n=46*	*n=5*		*0.830*	*n=82*	*n=5*		*0.188*
	CIMP.High	17%	10%			67%				2%	0%			20%	20%			0%	20%		
	CIMP.Low	20%	20%			16%				15%	10%			41%	20%			17%	40%		
	CIMP.Negative	64%	70%			16%				82%	90%			39%	60%			83%	40%		
Microsatellite status		*n=402*	*n=32*	0	*0.144*	*n=67*	*n=1*	0	*0.758*	*n=174*	*n=21*	0	*1.000*	*n=55*	*n=5*	0	*1.000*	*n=106*	*n=5*	0	*1.000*
	Stable (MSS)	82%	100%	(0,0.588)		15%	100%	(0,7.526)		99%	100%	(0,321.524)		84%	100%	(0,6.587)		96%	100%	(0,38.731)	
	Instable (MSI)	18%	0%			85%	0%			1%	0%			16%	0%			4%	0%		
**Selected mutations**																					
APC mut status		*n=363*	*n=29*	2.084	*0.758*	*n=60*	*n=1*	Inf	*0.760*	*n=152*	*n=18*	2.713	*1.000*	*n=49*	*n=4*	0.449	*0.585*	*n=102*	*n=6*	0.835	*1.000*
	Wild-type	30%	17%	(0.753,7.175)		73%	0%	(0.066,Inf)		14%	6%	(0.383,119.188)		31%	50%	(0.03,6.731)		29%	33%	(0.113,9.695)	
	Mutated	70%	83%			27%	100%			86%	94%			69%	50%			71%	67%		
BRAF mut status		*n=363*	*n=29*	0	*0.433*	*n=60*	*n=1*	0	*1.000*	*n=152*	*n=18*	0	*1.000*	*n=49*	*n=4*	0	*1.000*	*n=102*	*n=6*	0	*1.000*
	Wild-type	89%	100%	(0,1.119)		43%	100%	(0,30.971)		100%	100%	(0,Inf)		96%	100%	(0,72.542)		95%	100%	(0,21.874)	
	Mutated	11%	0%			57%	0%			0%	0%			4%	0%			5%	0%		
KRAS mut status		*n=363*	*n=29*	2.427	*0.302*	*n=60*	*n=1*	Inf	*0.760*	*n=152*	*n=18*	1.972	*0.758*	*n=49*	*n=4*	0.871	*1.000*	*n=102*	*n=6*	7.315	*0.758*
	Wild-type	60%	38%	(1.05,5.863)		73%	0%	(0.066,Inf)		66%	50%	(0.65,5.994)		22%	25%	(0.062,49.684)		60%	17%	(0.778,356.801)	
	Mutated	40%	62%			27%	100%			34%	50%			78%	75%			40%	83%		
TP53 mut status		*n=363*	*n=29*	0.897	*1.000*	*n=60*	*n=1*	Inf	*0.902*	*n=152*	*n=18*	0.579	*0.760*	*n=49*	*n=4*	0.633	*1.000*	*n=102*	*n=6*	1	*1.000*
	Wild-type	42%	45%	(0.391,2.092)		65%	0%	(0.045,Inf)		32%	44%	(0.192,1.802)		65%	75%	(0.011,8.593)		33%	33%	(0.135,11.577)	
	Mutated	58%	55%			35%	100%			68%	56%			35%	25%			67%	67%		

TCGA H&E slide B		Overall				CMS1				CMS2				CMS3				CMS4			
		Classified (n=430)	Unclassified (n=33)			Classified (n=73)	Unclassified (n=1)			Classified (n=189)	Unclassified (n=21)			Classified (n=58)	Unclassified (n=5)			Classified (n=110)	Unclassified (n=6)		
**Major molecular features**		Statistics	Statistics	Odds ratio (95% CI)	P value	Statistics	Statistics	Odds ratio (95% CI)	P value	Statistics	Statistics	Odds ratio (95% CI)	P value	Statistics	Statistics	Odds ratio (95% CI)	P value	Statistics	Statistics	Odds ratio (95% CI)	P value
CIMP cluster		*n=337*	*n=20*		*1.000*	*n=67*	*n=0*			*n=142*	*n=10*		*1.000*	*n=46*	*n=5*		*1.000*	*n=82*	*n=5*		*0.188*
	CIMP.High	17%	10%			67%				2%	0%			20%	20%			0%	20%		
	CIMP.Low	20%	20%			16%				15%	10%			41%	20%			17%	40%		
	CIMP.Negative	64%	70%			16%				82%	90%			39%	60%			83%	40%		
Microsatellite status		*n=402*	*n=32*	0	*0.144*	n=67	n=1	0	*0.758*	*n=174*	*n=20*	0	*1.000*	*n=55*	*n=5*	0	*1.000*	*n=106*	*n=6*	0	*1.000*
	Stable (MSS)	82%	100%	(0,0.588)		15%	100%	(0,7.526)		99%	100%	(0,337.516)		84%	100%	(0,6.587)		96%	100%	(0,31.059)	
	Instable (MSI)	18%	0%			85%	0%			1%	0%			16%	0%			4%	0%		
**Selected mutations**																					
APC mut status		*n=363*	*n=29*	2.084	*0.758*	*n=60*	*n=1*	Inf	*0.760*	*n=152*	*n=17*	2.554	*1.000*	*n=49*	*n=4*	0.449	*1.000*	*n=102*	*n=7*	1.041	*1.000*
	Wild-type	30%	17%	(0.753,7.175)		73%	0%	(0.066,Inf)		14%	6%	(0.358,112.565)		31%	50%	(0.03,6.731)		29%	29%	(0.159,11.51)	
	Mutated	70%	83%			27%	100%			86%	94%			69%	50%			71%	71%		
BRAF mut status		*n=363*	*n=29*	0	*0.433*	*n=60*	*n=1*	0	*1.000*	*n=152*	*n=17*	0	*1.000*	*n=49*	*n=4*	0	*1.000*	*n=102*	*n=7*	0	*1.000*
	Wild-type	89%	100%	(0,1.119)		43%	100%	(0,30.971)		100%	100%	(0,Inf)		96%	100%	(0,72.542)		95%	100%	(0,18.175)	
	Mutated	11%	0%			57%	0%			0%	0%			4%	0%			5%	0%		
KRAS mut status		*n=363*	*n=29*	2.427	*0.302*	*n=60*	*n=1*	Inf	*0.760*	*n=152*	*n=17*	2.217	*0.758*	*n=49*	*n=4*	0.871	*1.000*	*n=102*	*n=7*	3.675	*0.758*
	Wild-type	60%	38%	(1.05,5.863)		73%	0%	(0.066,Inf)		66%	47%	(0.712,7.038)		22%	25%	(0.062,49.684)		60%	29%	(0.569,40.347)	
	Mutated	40%	62%			27%	100%			34%	53%			78%	75%			40%	71%		
TP53 mut status		*n=363*	*n=29*	0.897	*1.000*	*n=60*	*n=1*	Inf	*0.902*	*n=152*	*n=17*	0.521	*0.760*	*n=49*	*n=4*	0.633	*1.000*	*n=102*	*n=7*	1.248	*1.000*
	Wild-type	42%	45%	(0.391,2.092)		65%	0%	(0.045,Inf)		32%	47%	(0.167,1.657)		65%	75%	(0.011,8.593)		33%	29%	(0.192,13.745)	
	Mutated	58%	55%			35%	100%			68%	53%			35%	25%			67%	71%		

GRAMPIAN H&E slide A		Overall				CMS1				CMS2				CMS3				CMS4			
		Classified (n=144)	Unclassified (n=79)			Classified (n=20)	Unclassified (n=16)			Classified (n=63)	Unclassified (n=41)			Classified (n=35)	Unclassified (n=8)			Classified (n=26)	Unclassified (n=14)		
**Major molecular features**		Statistics	Statistics	Odds ratio (95% CI)	P value	Statistics	Statistics	Odds ratio (95% CI)	P value	Statistics	Statistics	Odds ratio (95% CI)	P value	Statistics	Statistics	Odds ratio (95% CI)	P value	Statistics	Statistics	Odds ratio (95% CI)	P value
CIMP cluster		*n=86*	*n=32*		*0.26*	*n=11*	*n=5*		*0.647*	*n=40*	*n=20*		*0.134*	*n=24*	*n=5*		*0.847*	*n=11*	*n=2*		*0.295*
	CIMP.High	17%	19%			45%	40%			5%	10%			33%	40%			0%	0%		
	CIMP.Low	37%	22%			36%	20%			45%	20%			38%	20%			9%	50%		
	CIMP.Negative	45%	59%			18%	40%			50%	70%			29%	40%			91%	50%		
Microsatellite status		*n=121*	*n=60*	0	*0.552*	*n=19*	*n=14*	0	*0.244*	*n=51*	*n=30*	0	*1*	*n=30*	*n=7*	0	*1*	*n=21*	*n=9*	0	*1*
	Stable (MSS)	98%	100%	(0,4.886)		84%	100%	(0,3.22)		100%	100%	(0,Inf)		100%	100%	(0,Inf)		100%	100%	(0,Inf)	
	Instable (MSI)	2%	0%			16%	0%			0%	0%			0%	0%			0%	0%		
**Selected mutations**																					
APC mut status		*n=121*	*n=60*	2.084	*0.392*	*n=19*	*n=14*	Inf	*0.013*	*n=51*	*n=30*	0.584	*1*	*n=30*	*n=7*	0.675	*1*	*n=21*	*n=9*	0.414	*0.517*
	Wild-type	10%	5%	(0.533,11.971)		37%	0%	(1.31,Inf)		2%	3%	(0.007,47.083)		10%	14%	(0.044,40.764)		5%	11%	(0.005,35.29)	
	Mutated	90%	95%			63%	100%			98%	97%			90%	86%			95%	89%		
BRAF mut status		*n=121*	*n=60*	0	*0.552*	*n=19*	*n=14*	0	*0.244*	*n=51*	*n=30*	0	*1*	*n=30*	*n=7*	0	*1*	*n=21*	*n=9*	0	*1*
	Wild-type	98%	100%	(0,4.886)		84%	100%	(0,3.22)		100%	100%	(0,Inf)		100%	100%	(0,Inf)		100%	100%	(0,Inf)	
	Mutated	2%	0%			16%	0%			0%	0%			0%	0%			0%	0%		
KRAS mut status		*n=121*	*n=60*	1.203	*0.636*	*n=19*	*n=14*	0.368	*0.257*	*n=51*	*n=30*	1.467	*0.485*	*n=30*	*n=7*	2.925	*0.649*	*n=21*	*n=9*	2.579	*0.427*
	Wild-type	48%	43%	(0.617,2.36)		21%	43%	(0.057,2.085)		63%	53%	(0.533,4.048)		33%	14%	(0.288,151.455)		57%	33%	(0.411,20.449)	
	Mutated	52%	57%			79%	57%			37%	47%			67%	86%			43%	67%		
TP53 mut status		*n=121*	*n=60*	1.604	*0.309*	*n=19*	*n=14*	Inf	*0.057*	*n=51*	*n=30*	0.798	*0.753*	*n=30*	*n=7*	Inf	*0.306*	*n=21*	*n=9*	0.635	*0.666*
	Wild-type	20%	13%	(0.64,4.433)		26%	0%	(0.744,Inf)		14%	17%	(0.194,3.542)		23%	0%	(0.333,Inf)		24%	33%	(0.087,5.376)	
	Mutated	80%	87%			74%	100%			86%	83%			77%	100%			76%	67%		

GRAMPIAN H&E slide B		Overall				CMS1			
		Classified (n=144)	Unclassified (n=79)			Classified (n=14)	Unclassified (n=17)		
**Major molecular features**		**Statistics**	**Statistics**	**Odds ratio (95% CI)**	**P value**	**Statistics**	**Statistics**	**Odds ratio (95% CI)**	**P value**
CIMP cluster		*n=86*	*n=32*		*0.26*	*n=11*	*n=5*		*0.647*
	CIMP.High	17%	19%			45%	40%		
	CIMP.Low	37%	22%			36%	20%		
	CIMP.Negative	45%	59%			18%	40%		
Microsatellite status		*n=121*	*n=60*	0	*0.552*	*n=19*	*n=12*	0	*0.265*
	Stable (MSS)	98%	100%	(0,4.886)		84%	100%	(0,3.791)	
	Instable (MSI)	2%	0%			16%	0%		
**Selected mutations**									
APC mut status		*n=121*	*n=60*	2.084	*0.392*	*n=19*	*n=12*	Inf	*0.026*
	Wild-type	10%	5%	(0.533,11.971)		37%	0%	(1.106,Inf)	
	Mutated	90%	95%			63%	100%		
BRAF mut status		*n=121*	*n=60*	0	*0.552*	*n=19*	*n=12*	0	*0.265*
	Wild-type	98%	100%	(0,4.886)		84%	100%	(0,3.791)	
	Mutated	2%	0%			16%	0%		
KRAS mut status		*n=121*	*n=60*	1.203	*0.636*	*n=19*	*n=12*	0.28	*0.127*
	Wild-type	48%	43%	(0.617,2.36)		21%	50%	(0.041,1.667)	
	Mutated	52%	57%			79%	50%		
TP53 mut status		*n=121*	*n=60*	1.604	*0.309*	*n=19*	*n=12*	3.78	*0.363*
	Wild-type	20%	13%	(0.64,4.433)		26%	8%	(0.346,202.168)	
	Mutated	80%	87%			74%	92%		

GRAMPIAN H&E slide B		CMS2				CMS3			
		Classified (n=53)	Unclassified (n=41)			Classified (n=26)	Unclassified (n=9)		
**Major molecular features**		**Statistics**	**Statistics**	**Odds ratio (95% CI)**	**P value**	**Statistics**	**Statistics**	**Odds ratio (95% CI)**	**P value**
CIMP cluster		*n=40*	*n=20*		*0.315*	*n=24*	*n=5*		*0.321*
	CIMP.High	5%	10%			33%	40%		
	CIMP.Low	45%	25%			38%	0%		
	CIMP.Negative	50%	65%			29%	60%		
Microsatellite status		*n=51*	*n=32*	0	*1*	*n=30*	*n=8*	0	*1*
	Stable (MSS)	100%	100%	(0,Inf)		100%	100%	(0,Inf)	
	Instable (MSI)	0%	0%			0%	0%		
**Selected mutations**									
APC mut status		*n=51*	*n=32*	0.624	*1*	*n=30*	*n=8*	0.346	*0.279*
	Wild-type	2%	3%	(0.008,50.222)		10%	25%	(0.031,4.981)	
	Mutated	98%	97%			90%	75%		
BRAF mut status		*n=51*	*n=32*	0	*1*	*n=30*	*n=8*	0	*1*
	Wild-type	100%	100%	(0,Inf)		100%	100%	(0,Inf)	
	Mutated	0%	0%			0%	0%		
KRAS mut status		*n=51*	*n=32*	1.674	*0.265*	*n=30*	*n=8*	1.485	*1*
	Wild-type	63%	50%	(0.625,4.531)		33%	25%	(0.211,17.637)	
	Mutated	37%	50%			67%	75%		
TP53 mut status		*n=51*	*n=32*	1.112	*1*	*n=30*	*n=8*	Inf	*0.307*
	Wild-type	14%	12%	(0.254,5.67)		23%	0%	(0.39,Inf)	
	Mutated	86%	88%			77%	100%		

GRAMPIAN H&E slide B		CMS4			
		Classified (n=21)	Unclassified (n=12)		
**Major molecular features**		**Statistics**	**Statistics**	**Odds ratio (95% CI)**	**P value**
CIMP cluster		*n=11*	*n=2*		*0.295*
	CIMP.High	0%	0%		
	CIMP.Low	9%	50%		
	CIMP.Negative	91%	50%		
Microsatellite status		*n=21*	*n=8*	0	*1*
	Stable (MSS)	100%	100%	(0,Inf)	
	Instable (MSI)	0%	0%		
**Selected mutations**					
APC mut status		*n=21*	*n=8*	Inf	*1*
	Wild-type	5%	0%	(0.01,Inf)	
	Mutated	95%	100%		
BRAF mut status		*n=21*	*n=8*	0	*1*
	Wild-type	100%	100%	(0,Inf)	
	Mutated	0%	0%		
KRAS mut status		*n=21*	*n=8*	3.812	*0.215*
	Wild-type	57%	25%	(0.518,47.338)	
	Mutated	43%	75%		
TP53 mut status		*n=21*	*n=8*	0.534	*0.646*
	Wild-type	24%	38%	(0.068,4.669)	
	Mutated	76%	62%		
