# Supplementary material for: Image-based consensus molecular subtype (imCMS) classification of colorectal cancer using deep learning
Source: Gut. 2020 Jul 20;70(3):544–54. doi: 10.1136/gutjnl-2019-319866 (PMC7873419; doi:10.1136/gutjnl-2019-319866)
Supplement: Supplementary data [file gutjnl-2019-319866supp022.pdf]

Table S11

FOCUS 3x

n patients = 278	OS Univariate surivival analysis			
**Variable**	**HR**	**95%CI Low**	**95%CI High**	**p-value**
**CMS1 vs. CMS2**	3.26	2.29	4.65	5 x 10-9
**CMS3 vs. CMS2**	1.18	0.78	1.76	0.435
**CMS4 vs. CMS2**	1.42	1.04	1.95	0.029
**imCMS1 vs. imCMS2 (Slide A)**	3.07	2.11	4.48	5 x 10-9
**imCMS3 vs. imCMS2 (Slide A)**	1.26	0.82	1.93	0.286
**imCMS4 vs. imCMS2 (Slide A)**	1.42	1.05	1.91	0.023
**imCMS1 vs. imCMS2 (Slide B)**	4.45	3	6.59	1 x 10-13
**imCMS3 vs. imCMS2 (Slide B)**	1.22	0.81	1.85	0.338
**imCMS4 vs. imCMS2 (Slide B)**	1.42	1.05	1.91	0.023

n patients = 267	OS Multivariable surivival analysis (adjusted by gender, age, stage)			
**Variable**	**HR**	**95%CI Low**	**95%CI High**	**p-value**
**CMS1 vs. CMS2**	2.32	1.61	3.34	6 x 10-6
**CMS3 vs. CMS2**	1.04	0.68	1.58	0.867
**CMS4 vs. CMS2**	1.38	1	1.92	0.05
**Age**	1	0.98	1.01	0.677
**Gender**	1.14	0.87	1.5	0.343
**Stage**	2.37	1.9	2.95	2 x 10-14
**imCMS1 vs. imCMS2 (Slide A)**	2.55	1.73	3.78	3 x 10-6
**imCMS3 vs. imCMS2 (Slide A)**	1.15	0.74	1.77	0.537
**imCMS4 vs. imCMS2 (Slide A)**	1.38	1.02	1.89	0.039
**Age**	1	0.98	1.01	0.782
**Gender**	1.09	0.83	1.43	0.551
**Stage**	2.5	2	3.12	6 x 10-16
**imCMS1 vs. imCMS2 (Slide B)**	3.34	2.21	5.05	1 x 10-8
**imCMS3 vs. imCMS2 (Slide B)**	1.19	0.78	1.84	0.418
**imCMS4 vs. imCMS2 (Slide B)**	1.38	1.02	1.88	0.038
**Age**	1	0.99	1.02	0.916
**Gender**	1.11	0.84	1.46	0.452
**Stage**	2.45	1.95	3.07	7 x 10-15

OS: overall survival

TCGA 3x

**n patients=395**	**OS Univariate surivival analysis**			
**Variable**	**HR**	**95% CI Low**	**95% CI High**	**p-value**
**CMS1 vs. CMS2**	1.35	0.76	2.42	0.308
**CMS3 vs. CMS2**	0.42	0.17	0.99	0.048
**CMS4 vs. CMS2**	1.26	0.74	2.15	0.402
**imCMS1 vs. imCMS2**	1.88	1.07	3.3	0.027
**imCMS3 vs. imCMS2**	0.45	0.21	0.97	0.043
**imCMS4 vs. imCMS2**	1.18	0.67	2.08	0.571

**n patients=380**	**OS Multivariate surivival analysis (adjusted by age, gender, stage)**			
**Variable**	**HR**	**95% CI Low**	**95% CI High**	**p-value**
**CMS1 vs. CMS2**	1.41	0.75	2.67	0.285
**CMS3 vs. CMS2**	0.6	0.24	1.48	0.267
**CMS4 vs. CMS2**	1.04	0.59	1.82	0.894
**Age**	1.04	1.02	1.06	0.001
**Gender**	1.08	0.68	1.73	0.735
**Stage**	2.15	1.63	2.83	6 x 10-8
**imCMS1 vs. imCMS2**	1.78	0.95	3.32	0.07
**imCMS3 vs. imCMS2**	0.57	0.25	1.28	0.172
**imCMS4 vs. imCMS2**	1.03	0.57	1.88	0.91
**Age**	1.04	1.02	1.06	0.001
**Gender**	1.03	0.65	1.65	0.892
**Stage**	2.13	1.61	2.81	9 x 10-8

OS: overall survival

**N patients=395**	**PFI Univariate surivival analysis**			
**Variable**	**HR**	**95% CI Low**	**95% CI High**	**p-value**
**CMS1 vs. CMS2**	1.37	0.79	2.36	0.26
**CMS3 vs. CMS2**	0.59	0.29	1.22	0.155
**CMS4 vs. CMS2**	1.68	1.06	2.65	0.028
**imCMS1 vs. imCMS2**	1.36	0.79	2.35	0.265
**imCMS3 vs. imCMS2**	0.37	0.18	0.75	0.006
**imCMS4 vs. imCMS2**	1.37	0.86	2.19	0.19

**n patients=380**	**PFI Multivariate surivival analysis (adjusted by age, gender, stage)**			
**Variable**	**HR**	**95% CI Low**	**95% CI High**	**p-value**
**CMS1 vs. CMS2**	2	1.12	3.58	0.019
**CMS3 vs. CMS2**	1	0.47	2.11	0.998
**CMS4 vs. CMS2**	1.5	0.93	2.4	0.094
**Age**	1	0.98	1.02	0.876
**Gender**	0.83	0.55	1.25	0.376
**Stage**	2.32	1.82	2.96	1 x 10-11
**imCMS1 vs. imCMS2**	1.6	0.89	2.87	0.115
**imCMS3 vs. imCMS2**	0.52	0.25	1.08	0.081
**imCMS4 vs. imCMS2**	1.22	0.75	1.99	0.417
**Age**	1	0.99	1.02	0.797
**Gender**	0.84	0.55	1.27	0.4
**Stage**	2.19	1.72	2.78	1 x 10-10

PFI: progression-free interval

GRAMPIAN 12x

n patients=83	RFS Univariate surivival analysis			
**Variable**	**HR**	**95%CI Low**	**95%CI High**	**p-value**
**CMS1 vs. CMS2**	3.57	0.5	25.39	0.203
**CMS3 vs. CMS2**	2.14	0.36	12.83	0.403
**CMS4 vs. CMS2**	5.75	1.05	31.43	0.044
**imCMS1 vs. imCMS2 (Slide A)**	1.43	0.13	15.75	0.771
**imCMS3 vs. imCMS2 (Slide A)**	3.18	0.58	17.35	0.182
**imCMS4 vs. imCMS2 (Slide A)**	5.75	1.05	31.41	0.044
**imCMS1 vs. imCMS2 (Slide B)**	0	0	Inf	0.998
**imCMS3 vs. imCMS2 (Slide B)**	2.82	0.55	14.53	0.216
**imCMS4 vs. imCMS2 (Slide B)**	6.34	1.16	34.67	0.033

n patients=60	RFS Multivariate surivival analysis (adjusted by age, gender, stage)			
**Variable**	**HR**	**95%CI Low**	**95%CI High**	**p-value**
**CMS1 vs. CMS2**	2.42	0.32	18.08	0.388
**CMS3 vs. CMS2**	1.8	0.23	14.38	0.579
**CMS4 vs. CMS2**	5.99	0.92	38.87	0.061
**Age**	0.92	0.86	0.99	0.031
**Gender**	4.79	0.92	25.12	0.064
**Stage**	18.47	3.39	100.64	0.001
**imCMS1 vs. imCMS2 (Slide A)**	1.36	0.12	15.61	0.804
**imCMS3 vs. imCMS2 (Slide A)**	3.18	0.41	24.4	0.267
**imCMS4 vs. imCMS2 (Slide A)**	6.63	1	44.12	0.05
**Age**	0.92	0.86	0.98	0.016
**Gender**	3.96	0.75	20.83	0.105
**Stage**	15.78	3.07	81.15	0.001
**imCMS1 vs. imCMS2 (Slide B)**	0	0	Inf	0.998
**imCMS3 vs. imCMS2 (Slide B)**	2.55	0.44	14.82	0.299
**imCMS4 vs. imCMS2 (Slide B)**	7.23	1.12	46.58	0.037
**Age**	0.93	0.87	1	0.043
**Gender**	3.52	0.68	18.25	0.134
**Stage**	12.73	2.71	59.79	0.001

RFS: relapse-free survival
